# Effect of Ethanol on Differential Protein Production and Expression of Potential Virulence Functions in the Opportunistic Pathogen *Acinetobacter baumannii*

**DOI:** 10.1371/journal.pone.0051936

**Published:** 2012-12-20

**Authors:** Chika C. Nwugo, Brock A. Arivett, Daniel L. Zimbler, Jennifer A. Gaddy, Ashley M. Richards, Luis A. Actis

**Affiliations:** Department of Microbiology, Miami University, Oxford, Ohio, United States of America; The Scripps Research Institute and Sorrento Therapeutics, Inc., United States of America

## Abstract

*Acinetobacter baumannii* persists in the medical environment and causes severe human nosocomial infections. Previous studies showed that low-level ethanol exposure increases the virulence of *A. baumannii* ATCC 17978. To better understand the mechanisms involved in this response, 2-D gel electrophoresis combined with mass spectrometry was used to investigate differential protein production in bacteria cultured in the presence or absence of ethanol. This approach showed that the presence of ethanol significantly induces and represses the production of 22 and 12 proteins, respectively. Although over 25% of the ethanol-induced proteins were stress-response related, the overall bacterial viability was uncompromised when cultured under these conditions. Production of proteins involved in lipid and carbohydrate anabolism was increased in the presence of ethanol, a response that correlates with increased carbohydrate biofilm content, enhanced biofilm formation on abiotic surfaces and decrease bacterial motility on semi-solid surfaces. The presence of ethanol also induced the acidification of bacterial cultures and the production of indole-3-acetic acid (IAA), a ubiquitous plant hormone that signals bacterial stress-tolerance and promotes plant-bacteria interactions. These responses could be responsible for the significantly enhanced virulence of *A. baumannii* ATCC 17978 cells cultured in the presence of ethanol when tested with the *Galleria mellonella* experimental infection model. Taken together, these observations provide new insights into the effect of ethanol in bacterial virulence. This alcohol predisposes the human host to infections by *A. baumannii* and could favor the survival and adaptation of this pathogen to medical settings and adverse host environments.

## Introduction

The Gram-negative aerobic coccobacillus *Acinetobacter baumannii* is recognized for its ability to cause severe nosocomial infections including pneumonia, urinary tract infections, wound and burn infections, secondary meningitis and systemic infections [1], [2]. Although this microorganism is generally associated with infections in compromised hospitalized patients, community-acquired *A. baumannii* infections have become an increasing concern [3]. More recently, this bacterium has emerged as a serious threat to wounded military personnel deployed to Iraq and Afghanistan [4], [5], [6]. It has also been reported as the etiological agent of severe cases of necrotizing fasciitis [7], [8], [9] as well as skin and soft tissue infections [10]. These findings reflect the capacity of *A. baumannii* to cause a broadening spectrum of human diseases; a clinical problem that is further compounded by the emergence of multi- and pandrug-resistant strains [11].

*A. baumannii* causes colonization more often than infection and preferentially resides in medical environments and devices [2], [12], [13], [14]. This behavior could be due to its capacity to produce CsuA/BABCD-mediated pili [15] and the Bap protein [16], which are involved in biofilm formation; poly-ß-1-6-N-acetylglucosamine, which is critical for the formation of fully developed biofilms [17]; the outer membrane protein OmpA, which plays a role in bacteria-host interactions and cell apoptosis [18], [19], [20]; lipopolysaccharides, which are involved in host immune responses [21], [22]; a phospholipase D, which is responsible for serum resistance [23]; K1 capsular polysaccharide, which acts as an efficient protectin in experimental animal infections [24]; and to express acinetobactin-mediated iron acquisition functions, which are involved in bacterial persistence, cell damage and killing of infected hosts [25]. In spite of this progress, there is paucity in the understanding of the host and environmental signals that could affect the expression of the aforementioned *A. baumannii* virulence factors. It has been shown that environmental factors, such as light, as well as the presence of free iron, monovalent cations and ethanol in the culture medium affect antibiotic resistance, biofilm formation on abiotic surfaces, secretion of proteins and the virulence phenotype of this pathogen when tested in experimental infection models [15], [25], [26], [27], [28]. The role of ethanol is of particular interest; clinically, it affects the outcome of *A. baumannii* human chronic infections [29], [30] probably because of the up-regulation of Toll-like receptor 2 in epithelial cells, a host response that exacerbates inflammation and predisposition to disease [31]. Epidemiologically and practically, ethanol could play a role in the physiology of this pathogen. Because of the persistence and transmission of *A. baumannii* in medical environments as well as the morbidity and mortality of the infections it causes in humans, strict hygiene practices, including the use of ethanol-based hand rubs, gels and foams, have been implemented in most medical institutions. When properly used, these agents are effective in reducing bacterial loads among healthcare personnel and patients, the nosocomial transmission of pathogens and the incidence rate of hospital infections [32]. However, noncompliance with established protocols, a known drawback of alcohol-based hand hygiene practices [32], and the reduction of effective alcohol concentrations overtime, because of its volatility, could provide an opportunity for *A. baumannii* to persist in an environment where it can adjust to this antimicrobial agent. Accordingly, it was shown that low ethanol concentrations, particularly when added to minimal medium, significantly enhances the growth rate and the final cell density of *A. baumannii* cultures [26]. This study also showed that the addition of ethanol to culture medium promotes the differential secretion of bacterial proteins including the outer membrane protein OmpA, which has been shown to play a critical role in the capacity of *A. baumannii* to form biofilms and cause apoptotic death of human epithelial cells [18], [19], [20]. This finding is in agreement with the observation that co-culture of *A. baumannii* with *Saccharomyces cerevisiae* results in enhanced bacterial growth, a response due to fungal-mediated ethanol production [33]. This study also showed that the presence of low ethanol concentrations enhanced the ability of *A. baumannii* to withstand salt stress and its virulence against the bacterial predator *Caenorhabditis elegans*. Random insertion mutagenesis showed that the enhanced ethanol-mediated virulence response of *A. baumannii* ATCC 17978 against *C. elegans* worms as well as *Dictyostelium discoideum* amoebae relates to genes located in pathogenicity islands, some of which code for novel gene products [34]. More recently, the role of ethanol as a global gene regulator in *A. baumannii* was examined using RNA-sequencing [35]. This genome-wide analysis showed that besides inducing genes involved in its assimilation and utilization as a nutrient, ethanol controls the differential transcription of *A. baumannii* ATCC 17978 genes responsible for stress responses as well as the production of permeases, efflux pump proteins, a secreted phospholipase C, and proteins involved in phosphate and iron transport.

Although transcriptomic analysis is a convenient approach to examine global gene expression, it does not provide information on the modulation of gene expression at the post-transcriptional levels and poor correlations between mRNA transcript levels and those of their corresponding protein products have been reported [36]. Thus, proteomics is not only a proper approach to confirm transcriptomic data, but also a tool that provides a better understanding of differential gene expression in response to extracellular signals. In the case of *A. baumannii*, this global approach has already provided important information on the response of this pathogen to iron, antibiotics and salts [27], [37], [38]. In view of these observations, the work described here reports the effect of ethanol on differential protein production using 2-DE and mass spectrometry analysis of *A. baumannii* ATCC 17978 cultured in the absence or presence of ethanol. This approach showed the ethanol-dependent differential production of 34 proteins, some of which are involved in lipid and carbohydrate metabolism. These findings led to the observation that ethanol enhances biofilm formation, which correlates with a reduction of cell motility on semi-solid media. Furthermore, the presence of ethanol induces a reduction in the pH of the culture medium, a change that correlates with an increased production of indole-3-acetic acid (IAA). This metabolite is recognized as a signaling molecule that plays a role in bacterial survival and resistance to host defenses such as those expressed in plants [39]. Accordingly, growth of *A. baumannii* ATCC 17978 in the presence of ethanol significantly enhanced its virulence when tested using the Greater Wax Moth *Galleria mellonella* as experimental infection model.

## Materials and Methods

### Bacterial Strains and Growth Conditions

Bacteria were routinely grown overnight (12–14 h) at 37°C using Luria-Bertani (LB) agar or broth [40] supplemented with 0%, 1% or 2% ethanol. Liquid cultures were grown in a shaking incubator (200 rpm) at 37°C until late-logarithmic to stationary growth phase as determined by OD_600_. Ethanol concentrations were chosen based on previously reported work [33], [34]. Growth curves using LB and swimming broth (SB) (10 g/l tryptone, 5 g/l NaCl) were done to determine the effect of ethanol on bacterial growth at 37°C for 24 h. Growth curves were determined three times using fresh biological inocula each time. Changes in the pH of culture supernatants, cleared by high-speed centrifugation, in response to the presence of ethanol were measured using a UB-10 pH meter (Denver Instrument, USA) after incubation at 37°C for 24 h. Three replicates were used per treatment using uninoculated cultures as control. Experiments were repeated three times using fresh biological replicates each time.

### Biofilm, Carbohydrate and Cell Motility Assays

Biofilm formation on plastic was determined by crystal violet staining of overnight static cultures as described before [15]. The structure of biofilms was examined by scanning electron microscopy (SEM) of cells that grew attached to polystyrene coverslips cultured statically in unsupplemented LB broth or broth containing 1% or 2% ethanol at 37°C for 24 h in 50-ml conical tubes. The biofilm samples were washed, processed and viewed with a Zeiss Supra Gemini Series 35V scanning electron microscope as previously described [15].

To determine the carbohydrate content of biofilms, culture supernatants were decanted and the biofilms were rinsed with distilled water three times. Biofilm cells were scrapped from the plastic surface and suspended into 0.5 ml of distilled water, using 0.2 ml for protein quantification [41] and 0.2 ml for carbohydrate quantification using the L-cysteine monomeric quantification assay [42]. The carbohydrate/protein content ratio was used to represent the normalized carbohydrate content of the biofilms formed under different treatments. The biofilm and carbohydrate quantification assays were repeated three times with fresh biological samples each time.

Bacterial motility on semi-solid media was examined as described before [28] using swimming agarose alone or supplemented with 1% or 2% ethanol. Motility halos on the surface of the medium around the inoculation site were visually determined after incubation at 37°C for 24 h. Replicate experiments were repeated three times with fresh biological samples each time. Bacteria were lifted from the edge of the growth halos developed under the culture conditions described above onto Formvar-coated gold grids. The samples were negative-stained with 1.5% ammonium molybdate and visualized using a Zeiss EM 10C transmission electron microscope as previously described [15].

### Detection of IAA Production

The production of IAA was assayed colorimetrically using the Salkowski test [43]. Briefly, 1 ml of LB broth overnight cultures was used to inoculate 100 ml of LB or SB. Samples were incubated with agitation at 37°C for 24 h. Clear culture supernatants obtained by centrifugation at 13,500 rpm for 10 min were mixed 1∶1 (vol/vol) with a reagent containing 12 g/l of FeCl_3_ in 7.9 M H_2_SO_4_. After incubation in the dark at 24°C for 30 min, absorbance measurements were taken at OD_535_ for each sample. SB supplemented with 5 mM L-tryptophan was used to determine the effect of this amino acid in the production of IAA. These experiments were done in duplicate three times using fresh biological samples each time.

### Identification of Ethanol-responsive Proteins via Proteomics

Differential protein production was examined using two-dimensional gel electrophoresis (2-DE) and mass spectrometry as described before [38]. Briefly, *A. baumannii* ATCC 17978 cells were cultured overnight in a shaking incubator at 37°C in LB in the absence or the presence of 1% or 2% ethanol. The cell pellets were collected, stored and processed to obtain total cell protein samples as described before [38]. Total cell proteins were fractionated by 2-DE and the stained protein spots were analyzed using the PDQuest software package (version 7.3.0, Bio-Rad, USA). The entire procedure was repeated three times with fresh biological samples each time, representing three independent biological replications. Spots corresponding to proteins differentially produced in response to ethanol were excised from the gels, processed and then analyzed with a Bruker Ultraflex III MALDI-TOF/TOF Mass Spectrometer (Bruker, USA) as described before [38]. The mass spectrometry data was collected and analyzed as described for the study of the differential production of *A. baumannii* iron-regulated proteins [38].

### *G. mellonella* Virulence Assays

These assays were conducted as described for testing the role of the acinetobactin-mediated iron acquisition functions in the virulence of *A. baumannii* ATCC 19606^T^ [25]. Briefly, ATCC 17978 cells previously grown in LB supplemented with 0%, 1% or 2% ethanol were collected by centrifugation and resuspended in phosphate-buffered saline solution (PBS) by passing the suspension through a 26_G_⅜ needle to disassociate cell aggregates. Ten final-instar *G. mellonella* larvae (Vanderhorst, Inc., St. Mary’s, OH) weighing 250–350 mg were randomly selected and injected with 10^5 ^CFUs/larva (±0.5 log). After injection, the larvae were incubated at 37°C in the dark. Death was assessed at 24-h intervals over 6 days. Larvae were considered dead and removed if they displayed no response to probing. Non-injected larvae and larvae injected with sterile PBS were used as negative controls. The results from any experimental batch with more than two deaths in either control groups were excluded. Three independent *G. mellonella* killing assays, using a total of 30 animals for the control and experimental groups, were performed and the resulting survival curves were plotted using the Kaplan-Meier method [44].

### Statistical Analyses

For the proteomic experiments, pair-wise comparisons to determine significant differences in mean spot volumes between treatments were performed on standardized log_10_ values of protein spot volumes using the Student’s *t*-test (*P*<0.05) as provided by the PDQuest software package. The statistical significance of the differences in the number of *G. mellonella* larvae surviving at different time points after infection with bacteria cultured in the presence or absence of ethanol was determined using the log-rank test (SAS Institute Inc., Cary, NC). For all other analytical assays, ANOVA (SigmaPlot version 11) was applied and the Fisher’s Least Significant Difference (FLSD) test was used to separate means at *P*<0.05.

## Results and Discussion

### Validation of Ethanol-induced Virulence Response in *Galleria mellonella*

**Figure 1:**
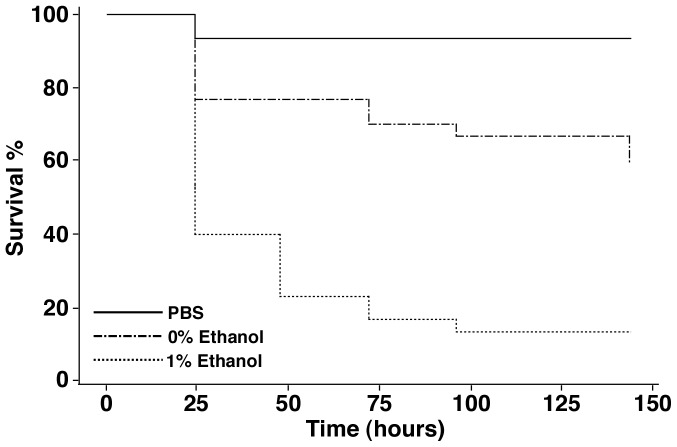
Effect of ethanol on the killing of *G. mellonella* infected with *A. baumannii* ATCC 17978. Larvae were injected with 10^5^ CFUs of *A. baumannii* ATCC 17978 cells grown in LB broth supplemented with 0% or 1% ethanol. Caterpillars injected with comparable volumes of sterile PBS were used as negative controls. After injection, the larvae (n = 30 per group) were incubated at 37°C and death was assessed at 24-h intervals over 144 h.

Previous studies on effects of ethanol on the virulence of *A. baumannii* ATCC 17978 were performed using *D. discoideum* and *C. elegans* as hosts [33], [34]. While these two experimental models provided valuable initial insights into the potential expression of *A. baumannii* functions involved in its interaction with complex microorganisms, these models may not reflect all the interactions between this pathogen and the human host. These considerations prompted us to test the role of ethanol in the virulence of *A. baumannii* ATCC 17978 using *G. mellonella*, an invertebrate host capable of mounting complex defense responses similar to those described in the human host [45]. We recently showed that this experimental infection model produces results similar to those collected with mice when testing the role of the acinetobactin-mediated iron acquisition system in the virulence of the *A. baumannii* ATCC 19606^T^ strain [25]. Infection of *G. mellonella* with ATCC 17978 cells grown overnight in LB broth containing 0% ethanol significantly reduced the survival probability of the larvae by 40% at 144 h post infection when compared with animals injected with sterile PBS (Fig. 1). The death rate of caterpillars injected with ATCC 17978 cells cultured in LB broth containing 1% ethanol was 83%, a virulence change greater than 2-fold when compared with the results obtained when caterpillars were injected with bacteria cultured in unsupplemented LB. These results not only agree with those previously obtained using different invertebrate hosts [33], [34], but also demonstrate the relevance of an alternative virulence model that could be used to study the role ethanol plays in the pathophysiology of *A. baumannii*. These observations also validate the experimental conditions we used to collect the data reported and discussed in the following sections.

### Detection of *A. baumannii* Proteins Differentially Produced in Response to Ethanol

**Figure 2:**
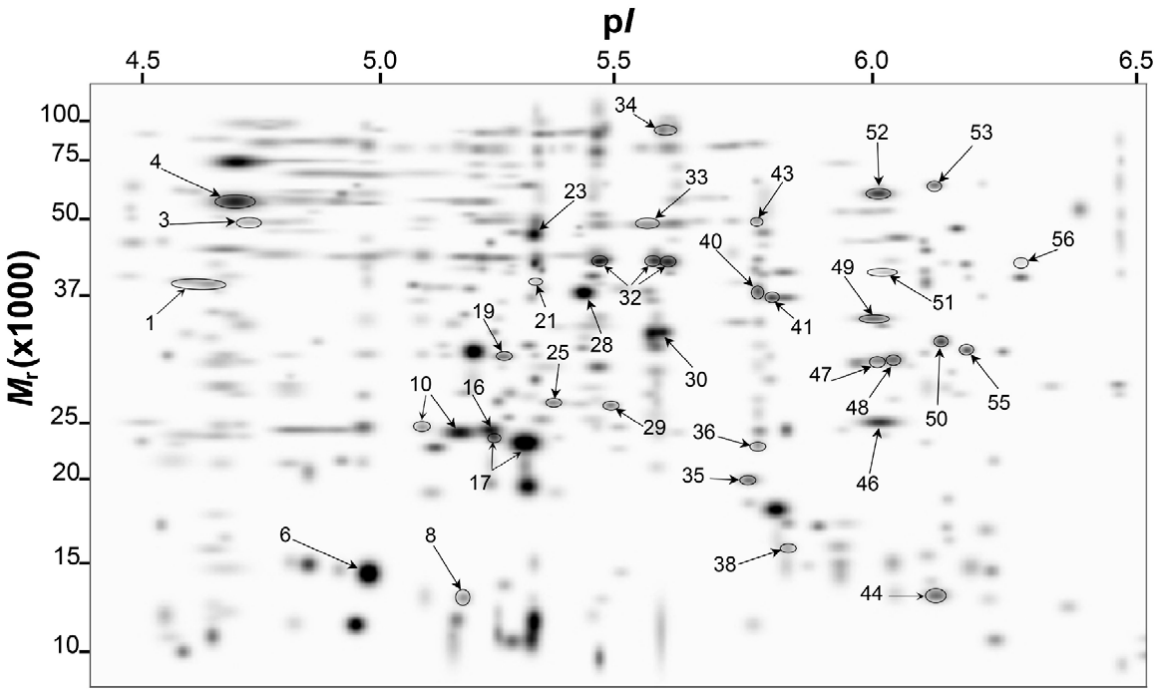
2D-polyacrylamide gel electrophoresis of *A. baumannii* ATCC 17978 whole-cell proteins differentially produced in response to ethanol. Representative PDQuest-generated master gel image showing the general pattern of matched protein spots isolated from bacteria grown in LB broth containing 0%, 1%, or 2% ethanol. Differentially expressed spots are numbered. *M*_r_, relative molecular weight; p*I*, isoelectric point. Although spots numbers range from 1 to 56, the spot numbering is not serialized and missing numbers represent proteins the production of which was not affected by the presence of 1% or 2% ethanol and were used for purposes different from those of this study.

**Figure 3:**
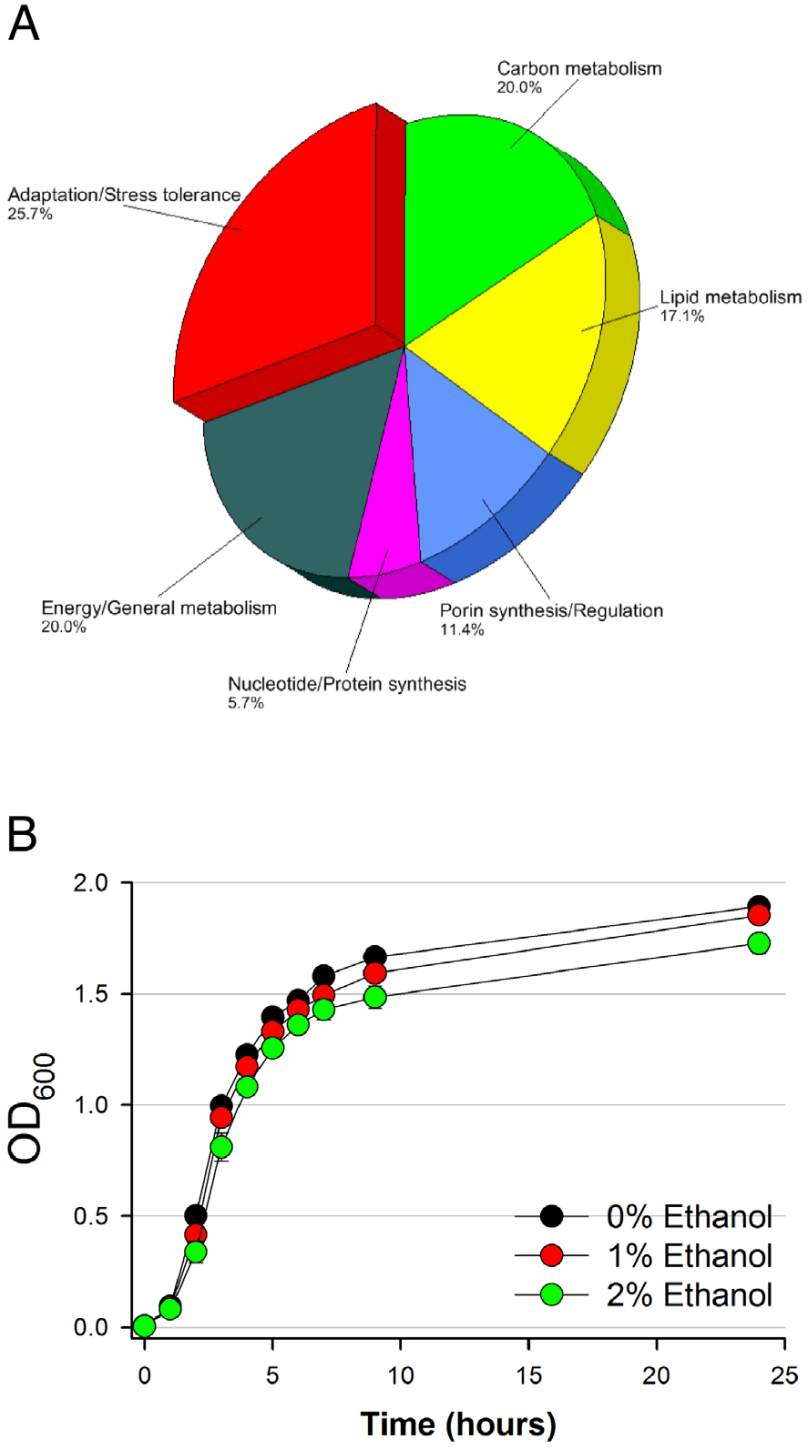
Functional categories of differentially produced proteins and growth curves of *A. baumannii* ATCC 17978 cells in response to ethanol. (A) Functional category distribution of differentially produced proteins by *A. baumannii* ATCC 17978 cells grown in LB supplemented with 0%, 1% or 2% ethanol. (B) Growth curves of *A. baumannii* ATCC 17978 cells cultured in LB supplemented with 0%, 1% or 2% ethanol. Error bars represent 1 standard error.

Resolution of *A. baumannii* ATCC 17978 whole-cell lysate proteins using 2-DE with a pH range between 4 and 7 resulted in the detection of over 600 spots per gel and at least 400 reproducible spots within replicate gels per treatment (Table S1). The master gel image generated with the Bio-Rad PDQuest software showing the general spot pattern detected across replicate gels per treatment highlights 39 identified protein spots differentially produced in *A. baumannii* ATCC 17978 cells in response to the presence of 1% or 2% ethanol in the culture medium (Fig. 2). A close-up view of the profiles of some selected spots from representative gels, which highlight variations in spot volumes in response to ethanol, is shown in panels A-O of Fig. S1. Differentially produced protein spots were further analyzed by mass spectrometry. It is important to mention that in certain instances more than one spot matched to a given protein (example spots 1, 10, 17, and 28), an outcome that could be due to a combination of factors including multimerism/protein isoforms, maturation state, and/or post-translational modifications as previously reported [38]. Thus, the 39 protein spots that were differentially produced in response to ethanol (Fig. 2) matched to 34 unique proteins/peptides (Tables 1, 2, 3 and 4). The predicted physiological functions of these proteins were grouped into the following categories: (a) adaptation/stress tolerance, (b) carbon metabolism, (c) lipid metabolism, (d) porin synthesis/regulation, (e) nucleotide/protein synthesis, and (f) energy production and general metabolism (Fig. 3A). The largest functional category was proteins involved in adaptation/stress tolerance (25.7%), followed by carbon metabolism and general metabolism proteins each accounting for 20%, respectively (Fig. 3A). In spite of identifying an increased production of proteins involved in adaptation/stress responses, we observed a significant but slight reduction in bacterial growth in the presence of 2% ethanol when compared with bacterial growth in the presence of 0% or 1% ethanol (Fig. 3B). This observation indicates that the biological fitness of bacteria grown overnight at 37°C in LB broth in the presence of ethanol was not significantly compromised. It is important to note that these culture conditions resemble those used to determine the global differential transcription of *A. baumannii* ATCC 17978 genes in response to ethanol as shown in Table S2 in the report by Camarena *et al.* [35]. Therefore, our proteomics data can be compared with the cognate transcriptomic information already published.

**Table 1:** Differential production of adaptation/stress tolerance-related proteins in response to ethanol.

Spot^a^	Protein name; strain^b^	Gene^b^	NCBI Accession #	Theoretical^c^		S^d^	M^e^	P^f^
				*M*_r_	p*I*			
4	Chaperonin GroEL, SDF	*groEL*	gi|169632653	56886	4.92	456	38	61
10	Superoxide dismutase, 17978	*sodB*	gi|126642383	24207	5.88	93	8	31
16	Glutathione peroxidase, 17978		gi|126640260	20138	4.88	123	11	49
17	Alkyl hydroperoxide reductase C22 subunit, 17978		gi|126641253	18202	4.96	104	10	56
25	Putative antioxidant protein, 17978		gi|126642887	18779	6.32	114	9	65
35	Peptidyl-prolyl cis-trans isomerase precursor (PPIase) (rotamase), SDF		gi|169633288	18533	5.40	116	10	75
36	ATP-dependent Clp protease proteolytic subunit, SDF	*clpP*	gi|169634426	22525	5.26	73	8	29
46	Putative protease, 17978		gi|193078237	21010	5.61	67	5	31

a The spot numbers correspond to the numbers given in Figures 2 and S1. Histograms displaying the average spot volumes for each protein differentially produced in response to ethanol are presented in Appendix S1.

b Protein function, name and gene were determined by http:/www.ncbi.nlm.nih.gov/BLAST/; *A. baumannii* strain where gene was reported, 17978 (ATCC 17978).

c Theoretical relative molecular weight (*M*_r_) and isoelectric point (p*I*) were calculated by http:/www.expasy.org/. Observed *M*_r_ and p*I* can be extrapolated from Figure 2.

d Mascot score of protein hit.

e Number of matched peptide masses. The sequences of matched peptides per spot are provided in Appendix S2.

f Percent sequence coverage.

**Table 2:** Differential production of carbon and lipid metabolism-related proteins in response to ethanol.

Spot^a^	Protein name; strain^b^	Gene^b^	NCBI Accession #	Theoretical^c^		S^d^	M^e^	P^f^
				*M*_r_	p*I*			
*Carbon metabolism*								
30	Malate dehydrogenase, 17978	*mdh*	gi|126643040	32400	5.00	166	19	56
33	NAD-dependent aldehyde dehydrogenase, 17978		gi|193077956	51319	5.29	173	10	26
34	Aconitate hydratase 1, 17978	*acnA*	gi|193076354	100032	5.25	126	20	22
41	Fructose-1,6-bisphosphate aldolase, 17978	*fda*	gi|162286736	37331	5.43	146	18	54
43	Fumarate hydratase, 17978	*fumA*	gi|193076292	54948	5.44	134	20	48
49	Putative UTP-glucose-1-phosphate uridylyltransferase, 17978		gi|193075977	31765	5.56	93	12	49
53	Succinate dehydrogenase flavoprotein subunit, 17978	*sdh*	gi|126642744	64049	6.07	208	24	44
*Lipid metabolism*								
40	Ketol-acid reductoisomerase, SDF	*ilvC*	gi|169634382	36830	5.39	134	12	42
47	NADH-dependent enoyl-ACP reductase, 17978	*fabI*	gi|126640605	28626	5.61	109	13	43
48	Putative enoyl-CoA hydratase II, 17978		gi|193077017	28878	5.76	113	13	46
50	Acetyl CoA carboxylase, beta subunit, AYE	*accD*	gi|169794791	32951	5.85	84	10	27
51	Acyl-CoA dehydrogenase, 17978		gi|126641422	36448	6.01	101	12	46
56	Thiolase, 17978		gi|126641390	35542	6.77	138	14	47

Table legends same as shown in Table 1.

**Table 3:** Differential production of porin synthesis, regulation, nucleotide and protein synthesis-related proteins and a protein of unknown function in response to ethanol.

Spot^a^	Protein name; strain^b^	Gene^b^	NCBI Accession #	Theoretical^c^			S^d^	M^e^		P^f^	
				*M*_r_	p*I*						
*Porin synthesis/Regulation*											
1	Outer membrane protein A, 16B	*ompA*	gi|129307154	36939	5.42		94	11		37	
28	Outer membrane protein A, 16B	*ompA*	gi|129307154	36939	5.42		106	13		38	
19	NarL family two-component response regulator, 17978	*narL*	gi|126640806	20352	5.34		104	11		61	
55	Osmolarity response regulator, SDF	*ompR*	gi|169632218	28797	5.91		179	19		65	
*Nucleotide/Protein synthesis*											
32	Elongation factor Tu, 17978	*ef-Tu*	gi|162286753	42865	5.21		87	14		33	
38	Nucleoside diphosphate kinase (NDK) (NDP kinase), SDF	*ndk*	gi|169634408	15453	5.52		97	6		53	
*Unknown function*											
6	Putative 17-kDa surface antigen, ACICU		gi|184157742	12424		4.70	116		7		77

Table legends same as shown in Table 1.

**Table 4:** Differential production of energy and general metabolism-related proteins in response to ethanol.

Spot^a^	Protein name, strain^b^	Gene^b^	NCBI Accession #	Theoretical^c^		S^d^	M^e^	P^f^
				*M*_r_	p*I*			
3	F0F1 ATP synthase subunit alpha, 17978	*atpA*	gi|162286757	55363	5.29	126	20	32
8	Membrane-bound ATP synthase, F1 sector, epsilon-subunit, AYE		gi|169797643	14530	4.83	90	8	62
21	Phenylacetate-CoA oxygenase/reductase PaaK subunit, 17978	*paaK*	gi|193077015	39763	5.03	91	11	31
23	F0F1 ATP synthase subunit beta, 17978		gi|162286755	50243	5.03	201	27	66
29	Putative lactam utilization protein, AYE		gi|169796478	26946	5.10	101	11	48
44	Phenylacetate-CoA oxygenase subunit PaaB, 17978	*paaB*	gi|126641383	11317	6.04	200	10	82
52	Urocanate hydratase, SDF	*hutU*	gi|169634859	61194	5.64	175	19	36

Table legends same as shown in Table 1.

### Analysis of *A. baumannii* Proteins Differentially Produced in Response to Ethanol

#### Adaptation/stress tolerance-related proteins

Our study showed that the presence of ethanol resulted in the increased production of eight proteins associated with adaptation/stress responses (Table 1). This group includes antioxidant proteins such as a superoxide dismutase (spot 10, which relate to annotated gene A1S_2343), a glutathione peroxidase (spot 16, which relates to annotated gene A1S_0159), an alkyl hydroperoxide reductase (spot 17, which relate to annotated gene A1S_1205) and a putative antioxidant protein (spot 25, which relates to annotated gene A1S_2863). We also observed an ethanol-induced accumulation of proteases (spots 36 and 46, which relate to annotated genes A1S_0476 and A1S_2785, respectively) and proteins with predicted chaperone/protein folding functions such as GroEL (spot 4, which relates to annotated gene A1S_2664) and a peptidyl-prolyl cis-trans isomerase (spot 35, which relates to annotated gene A1S_2109). Interestingly, only the genes coding for GroEL (spot 4), the alkyl hydroperoxide reductase (spot 17) and a putative protease (spot 46) were also found to be regulated in response to ethanol at the transcriptional level [35].

The overproduction of proteins related to stress response and chaperone/protein folding functions is in accordance with the observation that the presence of ethanol in the environment causes severe perturbations in protein stability/folding and elicits a strong heat-shock stress response including a cell-envelope response [46], [47]. The effects of these perturbations were recently examined in more detail using a global approach, which showed that the ability of *Escherichia coli* to tolerate ethanol depends on the proper differential expression of genes coding for a network of interrelated stress response pathways that allow bacteria to adapt and persist under such condition [48].

#### Carbon metabolism-related proteins

The presence of ethanol both enhances and reduces the production of proteins related to carbon/sugar metabolism (Table 2). It promotes the increased production of enzymes involved in carbohydrate metabolism including tri-carboxylic acid (TCA)-cycle enzymes, such as malate dehydrogenase (spot 30, which relates to annotated gene A1S_3025), fumarate hydratase (spot 43, which relates to annotated gene A1S_0480) and succinate dehydrogenase (spot 53, which relates to annotated gene A1S_2713), all of which would be necessary for the utilization of ethanol as a carbon source by *A. baumannii* as previously reported [35]. Interestingly, the production of the glycolytic enzyme fructose-1, 6-bisphophatase aldolase (spot 41, which relates to annotated gene A1S_1544) was also enhanced. Our analysis also showed that a NAD-dependent aldehyde dehydrogenase (spot 33, which relates to annotated gene A1S_2452) is overproduced in response to ethanol, which is different from that coded for by A1S_2102 and proposed to be involved in ethanol utilization as a carbon source [35]. With the exception of fumarate hydratase, the genes coding for these proteins overproduced in response to ethanol were also found to be up-regulated at the transcriptional level [35]. Such a response is in accordance with the fact that *A. baumannii* uses ethanol as a carbon source when growing under nutrient-limiting conditions [35]. However, during bacterial ethanol assimilation, ethanol dehydrogenase catalyzes the oxidation of ethanol to aldehyde, which is converted into acetate by aldehyde dehydrogenase [49], [50]. Aldehyde is toxic and therefore it might be critical for *A. baumannii* cells to sustain an increased production of aldehyde dehydrogenase in stationary phase cells cultured in the presence of ethanol. NAD-dependent aldehyde dehydrogenase is a multi-functional enzyme [51], which is proposed to play a dual role in the later discussed ethanol-induced production of IAA. Furthermore, ethanol dehydrogenase catalyzes the conversion of ethanol to acetaldehyde and could be involved in the first step in ethanol catabolism by *A. baumannii* ATCC 17978 cells. However, in contrast to the transcriptomic study [35], which showed an ethanol-mediated up-regulation of ethanol dehydrogenase gene (A1S_2098); our proteomic analysis did not identify an increase in the production of this enzyme. This discrepancy could be due to technical limitations of the approach we used to examine the global protein response to ethanol or the possibility that ethanol depletion after bacterial growth to stationary phase might have resulted in a modulation of ethanol dehydrogenase protein production below the 2-DE detection level.

Table 2 also shows that the production of aconitate hydratase (spot 34, which relates to annotated gene A1S_0558) is reduced in the presence of 1% but not 2% ethanol. Interestingly, the transcription of this gene was enhanced by the presence of this alcohol in the culture medium [35], an observation that may reflect differences in the transcriptional and post-transcriptional regulation of this particular gene. Regarding the differential production of this protein, Smith *et al.* [33] suggested that the threshold concentration of ethanol required for the ethanol-induced virulence response in *A. baumannii* is ∼1% and exposure to higher ethanol concentrations can trigger a transition from an ethanol-mediated hyper-virulent response to an ethanol-mediated toxicity state as detected when adding 2% ethanol to the medium. We therefore suggest that in the post-transcriptional response phase to ethanol, aconitate hydratase might be an initial responder in *A. baumannii* during the transition from a hyper-virulent state to a toxicity state. We also observed a significant reduction in the production of a putative UTP-glucose-1-phosphate uridylyltransferase (spot 49, which relates to annotated gene A1S_0062). This enzyme, which is also known as GalU, plays a role in galactose utilization and the synthesis of glycogen as well as the carbohydrate moieties of glycolipids, glycoproteins, and proteoglycans. Furthermore, this enzyme is involved in the production of lipopolysaccharide and capsule, cell components shown to play a role in the virulence of Gram-negative bacterial pathogens including *Klebsiella pneumoniae* and *Shigella flexneri* [52], [53]. Although our virulence study shows that the presence of ethanol increases the virulence of *A. baumannii* cells (Fig. 1), the specific effect(s) of a reduced production of the GalU ortholog in the physiology of this pathogen remains to be investigated.

Taken together, all these observations indicate that *A. baumannii* could tolerate the presence of ethanol in medical settings by reprogramming its metabolic pathways, a response that results in intracellular degradation and assimilation of this alcohol in a process that resembles that described in *E. coli* [48].

#### Lipid metabolism-related proteins

Unlike carbohydrate metabolism, the effect of ethanol on lipid metabolism-related proteins was more coordinated. On one hand, we identified two lipid anabolism-related proteins that were overproduced in response to ethanol (Table 2): an NADH-dependent enoyl-ACP reductase (spot 47, which relates to annotated gene A1S_0534) and the beta subunit of acetyl CoA carboxylase (spot 50, which relates to annotated gene A1S_2869). A ketol-acid reductoisomerase (spot 40, which relates to annotated gene A1S_0545) was also overproduced in response to ethanol. This protein, also known as IlvC, is involved in amino acid synthesis as well as in the synthesis of CoA, which is needed for numerous biosynthetic reactions including those involved in lipid production. The overproduction of the ketol-acid reductoisomerase and the acetyl CoA carboxylase beta subunit matches the ethanol-increased transcription of the cognate genes [35].

On the other hand, we identified three lipid catabolism-related proteins that were underproduced in response to ethanol (Table 2): a putative enoyl-CoA hydratase II (spot 48, which relates to annotated gene A1S_1342), an acyl-CoA dehydrogenase (spot 51, which relates to annotated gene A1S_1376) and a thiolase (spot 56, which relates to annotated gene A1S_1344). Interestingly, the putative enoyl-CoA hydratase II and thiolase enzymes are coded for by genes that may be part of the same operon and the transcription of which are up-regulated by the presence of ethanol [35]. This is another example in which ethanol could have a different effect on the transcriptional and posttranscriptional regulation of gene expression in *A. baumannii* by mechanisms that remain to be characterized.

Taken together, our observations indicate that the presence of ethanol in the medium promotes an overall lipid biosynthesis response, which may reflect changes in membrane composition in response to ethanol [48] or the production of extracellular products that could play a role in biofilm formation as described later. Both responses could have a relevant role in the virulence of *A. baumannii* and its capacity to cause infections in humans.

#### Porin synthesis- and regulation-related proteins

The presence of ethanol induced the overproduction of the response regulator OmpR (Table 3, spot 55, which relates to annotated gene A1S_3229), a response that matches the transcriptional response detected by transcriptomic analysis [35], but reduced the production of a NarL ortholog (Table 3, spot 19, which relates to annotated gene A1S_0748). NarL is a response regulator that belongs to the OmpR protein family. In *E. coli*, this transcriptional regulator, which is part of the NarL/NarX two-component regulatory system, activates the production of enzymes involved in anaerobic respiration [54]. Thus, underproduction of NarL might explain the overproduction of aerobic TCA-cycle associated enzymes in cells cultured in the presence of ethanol. Rabin and Stewart also suggested that NarL-mediated gene regulation follows the same model as OmpR-mediated gene regulation [54]. OmpR, which is part of the EnvZ/OmpR two-component regulatory system, activates *ompC* but represses *ompF* [55], [56]. OmpF is a non-specialized porin that allows the passage of slightly larger molecules, such as antibiotics and bile substances, compared to OmpC, which allows the passage of smaller molecules [57]. Interestingly, the *A. baumannii* OmpA protein is an ortholog of the *Pseudomonas aeruginosa* OprF, a “slow general porin” that allows the passage of even larger substances than OmpF [57]. Based on these observations and our data showing that ethanol reduces the production of OmpA (Table 3, spots 1 and 28, which relate to annotated gene A1S_2840), we speculate that the underproduction of OmpA in cells cultured in the presence of ethanol might be due to the induction of OmpR. This response could be a mechanism by which this bacterium regulates the in-flow of potentially toxic substances, such as ethanol and/or host response effectors. However, this response requires further investigation because Camarena *et al.* [35] showed an increase, although not significant, in the level of OmpA transcripts in response to ethanol and our preliminary immunoblot studies showed no significant differences in the amount of OmpA produced by *A. baumannii* ATCC 17978 cells cultured in LB containing 0%, 1% or 2% ethanol (data not shown).

#### Nucleotide and protein synthesis-related proteins

The presence of ethanol induced the over production of an ortholog of Ndk (Table 3, spot 38, which relates to annotated gene A1S_0498), an enzyme that is involved in the synthesis of nucleoside triphosphates other than ATP. In contrast, only 1% ethanol induced a significant underproduction of the elongation factor Tu (EF-Tu) (Table 3, spot 32, which relates to annotated gene A1S_0279). While the role of the differential production of Ndk in response to ethanol is not clear at the moment, the changes in EF-Tu are interesting considering the fact that this protein plays a biological function unrelated to polypeptide biosynthesis. A recent report describes the presence of EF-Tu on the surface of *A. baumannii* cells and outer membrane vesicles and shows its interaction with the host extracellular matrix protein fibronectin [58]. Equally interesting is the observation that fibronectin is a key host product that allows the adhesion of *A. baumannii* to A549 human alveolar epithelial cells [59]. Although this report shows the involvement of OmpA, a TonB-dependent copper receptor and a 34-kDa outer membrane protein, it does not describe the binding of fibronectin to EF-Tu, a discrepancy that could be due to the use of different strains in the studies described in these two reports. It is possible to speculate that the interaction of *A. baumannii* with A549 human alveolar epithelial cells is mediated by different bacterial surface proteins, the production of which is dependent on environmental stimuli that promote such interactions. This possibility also applies to the putative 17-kDa surface antigen overproduced by the presence of ethanol in the medium (Table 3, spot 6, which relates to annotated gene A1S_1383), which could play a role in the interaction of *A. baumannii* with abiotic and/or biotic surfaces found in medical settings and/or host components.

#### Energy production and general metabolism-related proteins

The presence of ethanol promoted the increased production of proteins related to ATP synthesis (Table 4), including the alpha and beta subunits of the F0F1 ATP synthase (spots 3 and 23, which relate to annotated genes A1S_0153 and A1S_0155, respectively) and the epsilon subunit of a membrane-bound ATP synthase (spot 8, which relates to annotated gene A1S_0156). Interestingly, all three proteins are coded for by genes that belong to the same operon where genes A1S_0153 and A1S_0155 were found to be transcriptionally overexpressed in response to ethanol [35]. The enhanced production of this type of proteins may reflect the energy requirements needed for cell adaptation to the stress condition imposed by the presence of ethanol in the medium. In contrast, ethanol significantly reduced the production of a putative lactam utilization protein (spot 29, which relates to annotated gene A1S_1267), and an urocanate hydratase (spot 52, which relates to annotated gene A1S_3407), whose gene is transcriptionally upregulated by ethanol [35]. Urocanate hydratase is associated with amino acid metabolism while the putative lactam utilization protein is predicted to be involved in lactam/carbohydrate metabolism. Interestingly, the phenylacetate degradation-related proteins PaaK (spot 21, which relates to annotated gene A1S_1340) and PaaB (spot 44, which relates to annotated gene A1S_1337) were underproduced and overproduced, respectively, in the presence of ethanol in the medium. Both proteins are coded for by genes that belong to an operon in which all components were found to be up-regulated in the presence of ethanol by global transcriptional analysis [35], highlighting once more significant differences in ethanol-mediated gene regulation responses in *A. baumannii* at the transcriptional and post-transcriptional. PaaK catalyzes the first reaction in the aerobic phenylacetate catabolic pathway, which ultimately results in the production of acetyl-CoA [60]. The ethanol assimilation process is considerably acetogenic; thus, it will be expected that other innate acetogenic processes, such as the degradation of phenylacetate, would be suppressed via a negative feedback mechanism that might contribute to a down-regulation of PaaK production.

### The Effect of Ethanol on Biofilm Formation and Motility on Semi-solid Surfaces

**Figure 4:**
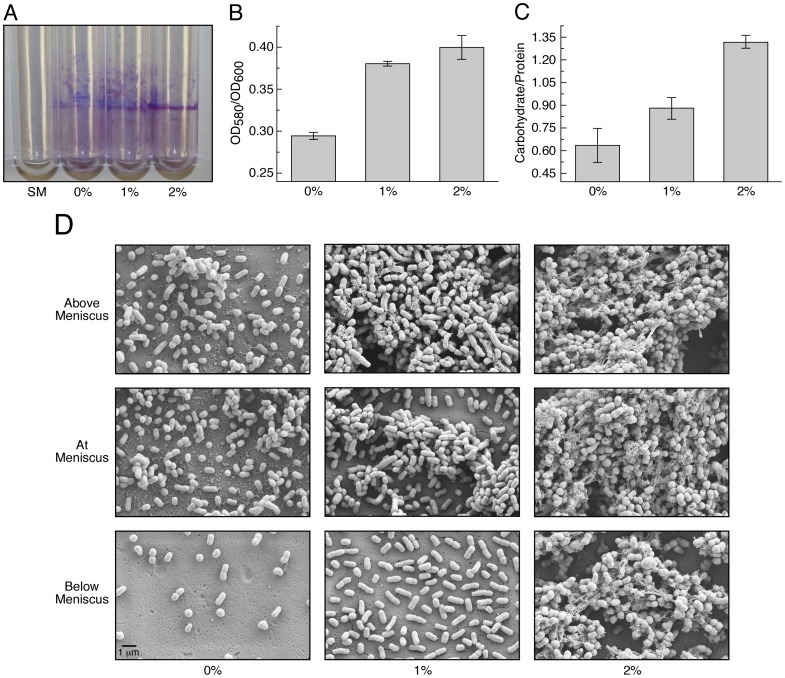
Effects of ethanol on biofilm formation and carbohydrate production. Biofilms formed by *A. baumannii* ATCC 17978 cells cultured in LB supplemented with 0%, 1% or 2% ethanol were visualized by crystal violet staining (A) and further quantified by colorimetry, after elution of the retained stain, in relation to the total biomass of each tested sample (B). A tube incubated with sterile medium (SM) was used as a negative control. (C) Carbohydrate content of biofilms formed on plastic by *A. baumannii* ATCC 17978 cells statically cultured in LB broth containing 0%, 1% or 2% ethanol determined with the L-cysteine monomeric carbohydrate assay. The carbohydrate concentration of biofilms was normalized to the protein content of each analyzed sample. Error bars shown in panels B and C represent 1 standard error. (D) Scanning electron microscopy of biofilms formed on plastic above, at and below the meniscus of LB broth static cultures containing 0%, 1% or 2% ethanol incubated overnight statically at 37°C. All micrographs were taken at a X25,000 magnification. The bar shown in the left bottom panel represents 1 µm.

Considering the importance of carbohydrates and lipids in biofilm formation [61] and the observation of ethanol inducing the production of proteins related to the metabolism of these cell compounds prompted us to investigate the effect of ethanol on biofilm formation on plastic. *A. baumannii* ATCC 17978 cells grown in the presence of 1% or 2% ethanol in LB broth produced more biofilms on polystyrene tubes than cells grown in the absence of ethanol (Fig. 4A and B). This finding seems to contradict the previous observation that ethanol has no effect on biofilm formation [34], a disagreement that could be due to technical differences between our study and that conducted previously.

Increase in biofilm formation was paralleled by a significant increase in the carbohydrate content of the biofilms formed on the plastic surfaces by cells cultured in the presence of 1% and 2% ethanol when compared to those formed in the absence of ethanol (Fig. 4C). SEM analysis confirmed the enhancing effects of ethanol on biofilm formation on plastic, particularly at and above the meniscus at the liquid-air interface (Fig. 4D). It is interesting to note that the biofilm structures formed by cells cultured in 2% ethanol were not only more complex even below the meniscus, but also included the enhanced formation of extracellular structures (see micrographs on the right column of Fig. 4D). These structures could represent ethanol-mediated overproduction of exopolymer that collapsed because of the dehydration step required for proper sample preparation. Alternatively, these structures could represent pili interconnecting bacteria differentially overproduced in response to the presence of ethanol in the medium.

**Figure 5:**
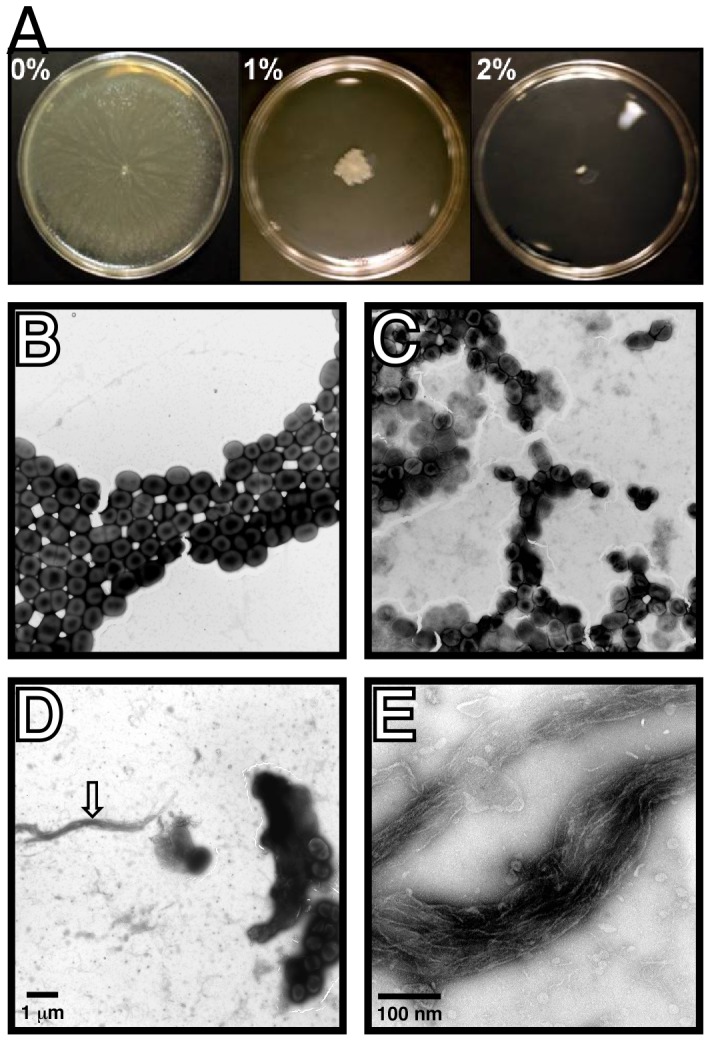
Effect of ethanol on bacterial motility and the production of extracellular structures. (A) Swimming plates supplemented with 0%, 1%, or 2% ethanol were inoculated with *A. baumannii* ATCC 17978 cells grown overnight on LB agar. (B-E) TEM of *A. baumannii* ATCC 17978 cells lifted from the edge of growth halos developed on swimming plates supplemented with 0% (B), 1% (C) or 2% ethanol (D and E) incubated 24 h at 37°C. The vertical arrow in panel D identifies the filamentous structure shown in panel E at higher magnification. Micrographs shown in B–D were taken at a X5,000 magnification, while the micrograph shown in E was taken at a X50,000 magnification. The bars shown in panels D and E represent 1 µm and 100 nm, respectively.

Biofilm initiation and development depends on a fine balance between motile and sessile lifestyles, a response that is not surprising since non-motile cells have a higher opportunity to attach to and form biofilm structures on solid substrates [62]. Accordingly, we observed that *A. baumannii* ATCC 17978 cells grown on swimming agarose plates in the absence of ethanol covered the entire plate after incubation at 37°C for 24 h (Fig. 5A). In contrast, the presence of 1% and 2% ethanol drastically reduced and almost abolished motility on 0.3% agarose plates, respectively, without significantly reducing the growth of bacteria cultured in swimming broth in the absence or presence of ethanol (data not shown). The reduction in cell motility could be due to an increased production of extracellular polymers or surface appendages as suggested by the SEM results described above. Such a possibility is supported by the data collected with TEM, which show that addition of ethanol to the medium results in an increased presence of electron-dense material surrounding the cells and the presence of extracellular structures that are not seen in the micrographs of cells grown on swimming agarose without ethanol (compare panels B-D in Fig. 5). Interestingly, some of the extracellular structures detected when bacteria were cultured in the presence of 2% ethanol are filamentous in nature (panels D and E of Fig. 5). Whether these structures represent dehydrated exopolysaccharides or bundled pili overproduced in response to ethanol is a possibility that remains to be examined.

Taken together, all these observations as well as those published before [35] demonstrate the profound effect of ethanol not only in differential gene expression and protein production, but also in the expression of cellular functions that could be playing a role in the virulence of *A. baumannii*.

### The Effect of Ethanol on pH and Indole-3-acetic Acid Production

**Figure 6:**
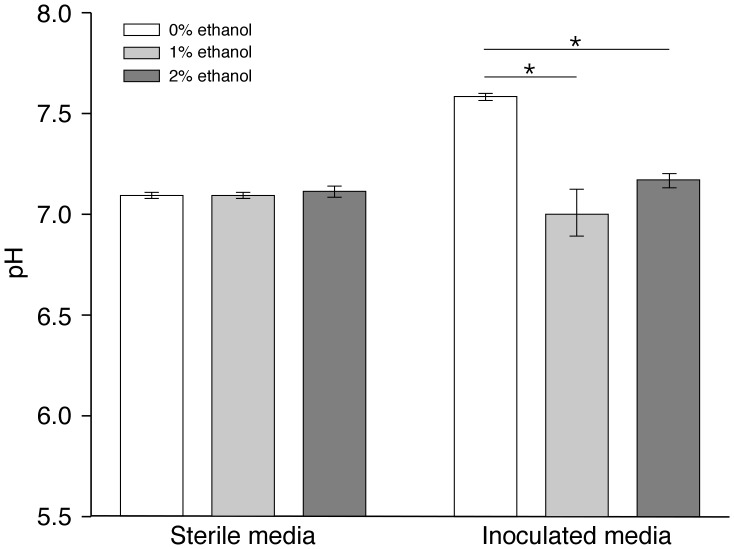
Effect of ethanol on the pH of *A. baumannii* ATCC 17978 cultures. Sterile and inoculated LB broth cultures supplemented with 0%, 1%, or 2% ethanol were incubated in an orbital shaker overnight at 37°C. The pH of the sterile samples and cultured supernatants obtained after centrifugation at 10,000 rpm for 10 min was determined immediately. Error bars represent 1 standard error. Horizontal bars with the asterisk (*) symbol identify samples with statistically significant differences (*P*<0.05) in pH values.

*A. baumannii* ATCC 17978 responds to the presence of ethanol in the culture medium by inducing the expression of genes coding for enzymes catalyzing the conversion of ethanol to acetate [35], a byproduct that could alter the pH of the culture medium if accumulated during the degradation of this alcohol. Interestingly, acetate oxidation is a bottleneck step in the metabolism of ethanol because the activity of enzymes involved in these processes are sensitive to environmental conditions that promote acetate accumulation in the medium [63]. Based on these considerations, we investigated a possible effect of ethanol on the pH of liquid cultures. Incubation of *A. baumannii* ATCC 17978 in LB broth supplemented with 1% or 2% ethanol resulted in a pH reduction from 7.5 to 7.0 of the culture supernatant of cells cultured overnight in the absence and presence of ethanol, respectively (Fig. 6). Although not shown, a similar trend in pH reduction was observed with samples examined at a particular cell density during the growth curve before reaching stationary phase. These observations suggest that *A. baumannii* secretes acidifying agents into the culture medium when grown in the presence of ethanol. These agents could alter global gene expression and protein production simply because of changes in pH, an environmental signal that has profound effects on bacterial physiology.

**Figure 7:**
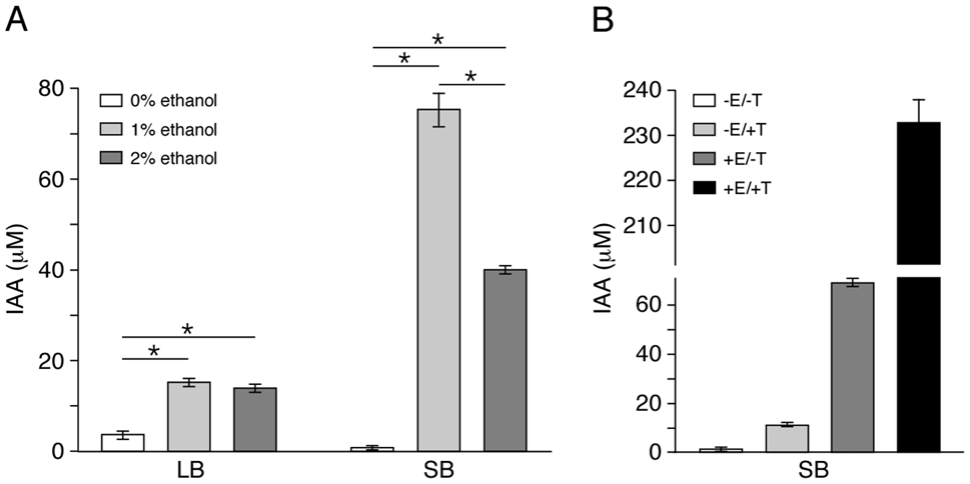
Effect of ethanol and/or tryptophan on IAA production. (A) The concentration of IAA in LB or SB *A. baumannii* ATCC 17978 cultures supplemented with 0%, 1% or 2% ethanol was determined after 24 h incubation at 37°C. (B) The effect of 5 mM L-tryptophan and/or 1% ethanol supplementation on the production of IAA bacteria cultured in SB after 24 h incubation 37°C. The plus (+) and minus (−) signs denote the addition or not of ethanol or tryptophan. IAA concentration values were normalized to an OD_600_ of 1.0 of each tested sample. Error bars represent 1 standard error. Horizontal bars with the asterisk (*) symbol identify samples with statistically significant differences (*P*<0.05) in IAA concentration values. All differences in IAA concentrations shown in panel B are statistically significant (*P*<0.05).

The metabolic bottleneck that results in the accumulation of acetate when *A. baumannii* is cultured in the presence of ethanol could also lead to the production of other derivatives that significantly affect the physiology of bacteria. Among these is indole-3-acetic acid (IAA), an acetic acid derivative that has been shown to differentially regulate gene expression, induce resistance to stress and increase the production of biofilms, lipopolysaccharides and exopolymeric substances in bacteria [64], [65], [66], [67]. Consequently, our observations of ethanol-mediated overproduction of stress-response related proteins (Table 1) and enhanced formation of biofilms (Fig. 4) led us to investigate the possibility of an ethanol-induced production/secretion of IAA. Our analysis revealed that *A. baumannii* ATCC 17978 cells produce more IAA when grown in the presence of 1% or 2% ethanol when compared to unsupplemented medium (Fig. 7A). This response was detected in cells grown in either LB or SB, although the response by cells cultured in SB was more evident than that detected with bacteria cultured in LB broth (Fig. 7A). The higher IAA production by cells cultured in the presence of 1% ethanol is in agreement with the threshold concentration needed to induce differential gene expression and virulence response in *A. baumannii* [34], [35].

Indole-3-acetic acid biosynthesis in bacteria generally occurs via tryptophan-dependent degradation pathway(s), although tryptophan-independent pathways seem to be active in different bacteria [39], [68]. Thus, we investigated whether *A. baumannii* ATCC 17978 cells produce IAA in a tryptophan-dependent and/or tryptophan-independent manner when cultured in SB in the presence or absence of 1% ethanol. The addition of tryptophan to SB containing no ethanol results in an 18-fold increase in IAA production (compare samples labeled -E/−T and -E/+T) (Fig. 7B), an observation that supports the expression of a tryptophan-dependent pathway already described in bacteria. On the other hand, the presence of 1% ethanol in otherwise unsupplemented SB resulted in a 114-fold increase in IAA production (compare samples labeled −E/−T and +E/−T), which was further increased by more than 3-fold by the presence of tryptophan in the ethanol-supplemented SB medium (compare samples labeled +E/−T and +E/+T) (Fig. 7B). These responses strongly indicate that *A. baumannii* ATCC 17978 expresses tryptophan-dependent as well as tryptophan-independent IAA biosynthetic pathways, both of which are influenced by the presence of ethanol in the medium. This response is not unique to the strain ATCC 17978 but rather common among *A. baumannii* clinical isolates; ten different strains showed the production of IAA in the presence of ethanol although the amount produced by these isolates varied among them (Table 5).

**Table 5:** Ethanol-induced IAA production in different *A. baumannii* clinical strains.

Strains	Relevant Characteristics	Isolation	Growth^a^	IAA (µM)^b^	Source/Reference
17978	First sequenced strain	Meningitis	0.94±0.02	77.35±4.66	ATCC
19606	Type strain	Urine	1.17±0.01	25.84±1.82	ATCC
RUH00875	Ref. strain, EU clone I	Urine	0.70±0.02	62.87±4.34	[74]
LUH08809	EU clone I	Wound	0.71±0.02	35.47±3.74	[75]
RUH00134	Ref. strain, EU clone II	Urine	0.75±0.03	88.12±6.26	[74]
LUH13000	Clinical isolate, EU clone II	Sputum	0.96±0.04	37.17±6.58	[74]
LUH05875	Ref. strain, EU clone III	Blood	1.05±0.04	92.17±6.87	[76]
LUH07672	EU clone III	Throat	1.32±0.03	91.51±10.17	[75]
AYE	Multidrug resistant	Blood	1.17±0.02	72.39±4.34	ATCC
A118	Naturally competent	Blood	1.30±0.03	71.13±5.24	[77]

a OD_600_ (mean ± S. E.) after incubation in a rotating shaker (200 rpm) for 24 h at 37°C. All strains were cultured in SB containing 1% ethanol.

b Calculated as the concentration of IAA (mean ± S. E.) in cleared culture supernatants normalized to an OD_600_ of 1.0.

### Proposed Indole Acetic Acid Biosynthetic Pathway

**Figure 8:**
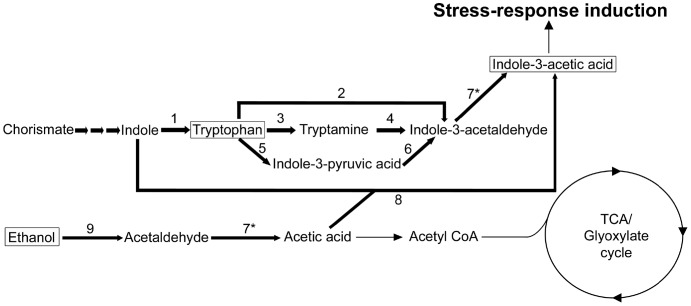
Proposed pathways for ethanol-induced IAA production in *A. baumannii*. The pathways were developed taking into account information previously published [35], [39], [68] and experimental data collected in this work. Numbers next to arrows refer to the enzymes listed in Table 6. The asterisk (*) symbol denotes a suggested dual role for aldehyde dehydrogenase in the conversion of indole-3-acetaldehyde to indole-3-acetic acid and the conversion of acetaldehyde to acetic acid as previously demonstrated [51].

Considering our experimental data showing that *A. baumannii* differentially produces IAA in response to ethanol via tryptophan-dependent and tryptophan-independent processes, the cognate bacterial biosynthetic pathways already proposed [39], [68], the transcriptomic data and the ethanol-assimilation pathway proposed by Camarena *et al.* [35], we screened the *A. baumannii* genome using bioinformatic tools for potential genes and gene products that could be involved in IAA production via tryptophan-dependent and tryptophan-independent pathways that were not identified by Camarena *et al.* [35] or us using transcriptomics and proteomics, respectively. Using this multifaceted approach, we identified nine *A. baumannii* genes and gene products (Table 6) that could be involved in IAA production as proposed in the pathways shown in Fig. 8. The upper part of the pathway includes the tryptophan-dependent IAA biosynthetic steps, which involve the enzymes tryptophan synthase (enzyme 1), tryptophan side-chain oxidase (enzyme 2), tryptophan decarboxylase (enzyme 3), amine oxidase (enzyme 4), tryptophan aminotransferase (enzyme 5), indole-3-pyruvate decarboxylase (enzyme 6) and aldehyde dehydrogenase (enzyme 7), which catalyzes the final step in the production of IAA from indole-3-acetaldehyde. While the enhanced production of IAA by *A. baumannii* cells cultured in the presence of exogenous tryptophan is in accordance with the response detected with other bacteria, particularly with those associated with plants where this amino acid plays a key role in bacteria-plant interactions [39], the role of ethanol in the upregulation of this pathway is practically unknown.

**Table 6:** Putative genes in proposed ethanol-induced IAA biosynthesis pathway in *A. baumannii* ATCC 17978.

Enzyme^a^	Accession number	Putative function	Ortholog	Organism	Identity/Similarity	Reference^b^
1	YP_001085883.1	Tryptophan synthase subunit β	CAL26229.1	*Bacillus amyloliquefaciens*	58%/76%	[68]
2	ABO11754.2	Tryptophan side-chain oxidase	YP_258540.1	*Pseudomonas fluorescens*	55%/70%	[78]
3	YP_001085473.1	Tryptophan decarboxylase	CAB81456.1	*Arabidopsis thaliana*	28%/47%	[79]
4	ABO12281.2	Amine oxidase	YP_001335129.1	*Klebsiella pneumoniae*	63%/77%	[80]
5	YP_001083734.1	Tryptophan aminotransferase	YP_003613824.1	*Enterobacter cloacae*	31%/50%	[81]
6	YP_001085470.1	Indole-3-pyruvate decarboxylase	AAG00523.2	*Enterobacter cloacae*	38%/54%	[82]
7	ABO12870.2	Aldehyde dehydrogenase	XP_758655.1	*Ustilago maydis*	39%/59%	[51]
8	YP_001085148.1	IAA acetyltransferase	CAL26203.1	*Bacillus amyloliquefaciens*	33%/49%	[68]
9	YP_001085127.1	Alcohol dehydrogenase	N/A^c^	*A. baumannii* ATCC 17978	N/A^c^	[35]

a Enzyme numbers correspond to numbers shown on Figure 8.

b References to homologous genes that have been demonstrated to be involved in ethanol assimilation, tryptophan catabolism and/or IAA biosynthesis.

c Not applicable.

The pathways shown in Fig. 8 also include a process in which indole is converted into IAA independently of tryptophan metabolism. Although this predicted process has been detected in other bacteria [39], [68], the full enzymatic mechanism catalyzing this process remains to be elucidated. However, based on our data we propose that *A. baumannii* ethanol degradation by an alcohol dehydrogenase (enzyme 9) results in the production of acetaldehyde, which is further metabolized into acetic acid by an aldehyde dehydrogenase (enzyme 7) that is overproduced in response to the presence of exogenous ethanol (Table 2). Our proposed pathway suggests a dual function for the aldehyde dehydrogenase as shown in our model (Fig. 8). Such a possibility is supported by the observation that a NAD-dependent aldehyde dehydrogenase catalyzes the conversion of indole-3-acetaldehyde to indole-3-acetic acid as well as acetaldehyde into acetic acid in *Ustilago maydis* [51]. Additionally, Idris *et al.* [68] showed that the production of IAA in *Bacillus amyloliquefaciens* by a tryptophan-independent pathway depends on an acetyltransferase-catalyzed reaction. Thus, the formation of IAA from indole via a tryptophan-independent pathway could be explained by the accumulation of acetate in response to ethanol degradation and the catalytic activity of a putative IAA acetyltransferase (enzyme 8). Such a process could explain the enhanced production of IAA by cells cultured in SB in the presence of 1% ethanol but in the absence of exogenous tryptophan. This process should also require increased availability of indole by cellular mechanisms that remain to be elucidated. However, considering the fact that this compound is formed from chorismate, which is also a precursor for the iron-regulated production of siderophore components, such as the dihydroxybenzoic acid moiety present in the *A. baumannii* siderophore acinetobactin [69], it is possible to speculate that iron-limiting conditions could favor the formation of IAA via a tryptophan-independent pathway. Furthermore, the transcriptomic analysis of ATCC 17978 cells cultured in the presence of ethanol showed an increased expression of genes coding for iron acquisition functions [35].

### Concluding Remarks

Comparative analysis of *A. baumannii* ATCC 17978 products for which data was collected using transcriptomics and proteomics shows that there are 13 direct and six inverse correlations between the transcription of genes and the production of their cognate proteins in response to ethanol. Direct correlations were observed in all three genes and cognate proteins involved in stress tolerance and four out of five genes and gene products involved in carbon metabolism. Interestingly, while all four lipid metabolism genes were transcriptionally upregulated by ethanol, only the production of enoyl-CoA hydratase II and thiolase were underproduced in response to ethanol although both lipid anabolic proteins are coded for by polycistronic operons. A direct correlation was also observed in the ethanol-mediated differential transcription and translation of the genes coding for the OmpR regulator, the alpha and beta subunits of the ATP synthase and the PaaB subunit of the phenylacetate-CoA oxygenase. In contrast, ethanol enhances the transcription but reduces the translation of the genes coding for OmpA, the Paak subunit of the phenylacetate-CoA oxygenase/reductase subunit and the urocanate hydratase. Furthermore, ethanol has different effects on the transcription and translation of genes that belong to polycistronic operons, as it is in the case of the *paaK* and *paaB* genes. Taken together, all these observations indicate that ethanol controls gene expression at the transcriptional and post-translational levels by mechanisms that overall remain to be elucidated in *A. baumannii*. The transcriptomic and proteomic data also indicate that ethanol regulates a wide range of traits associated with central metabolism as well as cellular functions that play a role in the interaction of this pathogen with the surrounding environment and its virulence when tested with different experimental infection models. Our results together with the experimental data published by Camarena *et al.* [35] and clinical observations previously reported [3], [29], [30] have provided a better understanding of the role ethanol, a chemical substance widely consumed by humans and used in medical settings worldwide, plays in the biology of *A. baumannii* and the pathogenesis of the infections it causes in humans. From the standpoint of the human host, it seems that there is a potential correlation between alcoholism and susceptibility to *Acinetobacter* infections [3], [29], [30]. It is also possible that potential interactions between *A. baumannii* and fungi normally found in the human host, such as *C. albicans*, could provide localized environmental niches in which ethanol production could induce bacterial stress and virulence responses. Although this is an attractive possibility supported by published experimental data obtained using *in vitro* conditions [33], such association in the human host remains to be confirmed. From the stand point of the bacterial pathogen and the different environments it encounters in medical settings, it is possible to speculate that although alcohol-based disinfectants normally used in medical facilities contain high amounts of ethanol, the high volatility of this alcohol and the persistence of *A. baumannii* in medical equipment and the human host as biofilms could explain some of our observations as well as those made by other investigators. Its high volatility would significantly reduce the effective disinfectant levels and biofilms most likely promote a protective environment in which *A. baumannii* cells would have the opportunity to mount cellular responses needed not only to resist ethanol but also to persist and cause infection.

Considering the fact that all *A. baumannii* tested strains produce IAA in response to ethanol, we propose that the ethanol-dependent enhanced virulence detected using different infection experimental models is due, at least in part, to the production of this compound. Thus, the production of IAA in response to stimuli found in the human host could promote important host-bacteria interactions involved in the pathogenesis of human infections similar to those described for plant-bacteria processes. These processes include adaptation to stress conditions and the expression of bacterial functions that promote communication with the plant host and the interaction with its components and the products it secretes [39]. Furthermore, our preliminary observations and those recently published [70] indicate that *A. baumannii* is able to efficiently utilize IAA as a carbon source. Thus, the presence of exogenous IAA could not only promote bacterial growth but also favor stress and virulence responses. Interestingly, human body fluids contain significant amounts of IAA and some derivatives, such as 5-hydroxyindole-3-acetic acid (5-HIAA) [71], [72], [73], which could promote the growth and virulence of *A. baumannii* and the subsequent pathogenesis of the infections it causes in the human host.

## Supporting Information

Figure S1 **Close-up view of some differentially produced protein spots in representative 2-DE gels from total-cell proteins obtained from** ***Acinetobacter baumannii*** **ATCC 17978 cells grown in LB broth containing 0%, 1%, or 2% ethanol is shown in panels A–O.** A total amount of 200 µg of protein was loaded on a pH 4–7 IpG strip and protein spots were visualized by staining with Coomassie Brilliant Blue after electrophoresis in polyacrylamide under denaturing conditions.
(TIF)

Table S1 **2-DE separation parameters of whole-cell lysate proteins of** ***Acinetobacter baumannii*** **ATCC 17978 cells grown in culture medium unsupplemented (0%) or supplemented with (1% or 2%) ethanol.** Data represents Means ± SD.
(DOC)

Appendix S1 **Histograms showing average spot volumes of the 35 identified** ***Acinetobacter baumannii*** **ATCC 17978 total cell proteins that were differentially produced in response to low-level ethanol treatments.** Bars with the same letter are not significantly different at P<0.05.
(PDF)

Appendix S2 **List of the 35 identified** ***Acinetobacter baumannii*** **ATCC 17978 total cell proteins that were differentially produced in response to low-level ethanol treatments showing the Mascot match results and the sequences of matched peptides.**
(PDF)
