# Supplementary figures and images for: Effect of Ethanol on Differential Protein Production and Expression of Potential Virulence Functions in the Opportunistic Pathogen Acinetobacter baumannii
Source: PLoS One. 2012 Dec 20;7(12):e51936. doi: 10.1371/journal.pone.0051936 (PMC3527336; doi:10.1371/journal.pone.0051936)

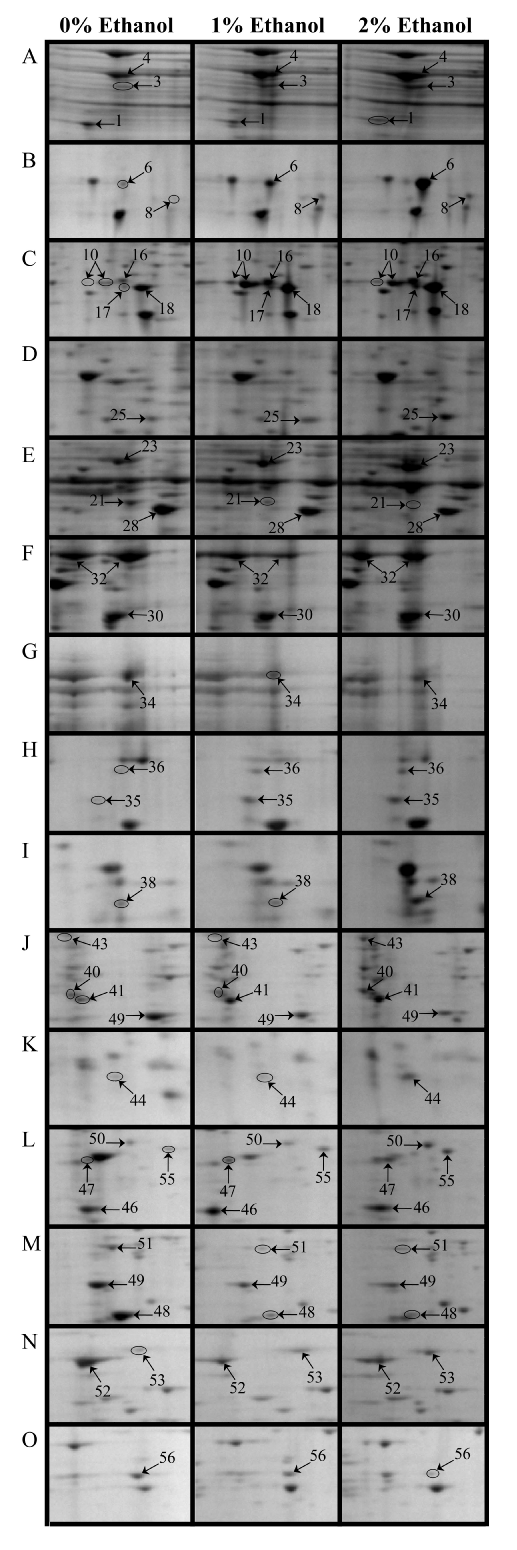

Supplement: Figure S1 — Close-up view of some differentially produced protein spots in representative 2-DE gels from total-cell proteins obtained from Acinetobacter baumannii ATCC 17978 cells grown in LB broth containing 0%, 1%, or 2% ethanol is shown in panels A–O. A total amount of 200 µg of protein was loaded on a pH 4–7 IpG strip and protein spots were visualized by staining with Coomassie Brilliant Blue after electrophoresis in polyacrylamide under denaturing conditions. (TIF) [file pone.0051936.s001.tif]
