# Supplementary material for: Effect of Ethanol on Differential Protein Production and Expression of Potential Virulence Functions in the Opportunistic Pathogen Acinetobacter baumannii
Source: PLoS One. 2012 Dec 20;7(12):e51936. doi: 10.1371/journal.pone.0051936 (PMC3527336; doi:10.1371/journal.pone.0051936)
Supplement: Table S1 — 2-DE separation parameters of whole-cell lysate proteins of Acinetobacter baumannii ATCC 17978 cells grown in culture medium unsupplemented (0%) or supplemented with (1% or 2%) ethanol. Data represents Means ± SD. (DOC) [file pone.0051936.s002.doc]

**Table S1.** 2-DE separation parameters of whole-cell lysate proteins of *A. baumannii* ATCC 17978 cells grown in culture medium unsupplemented (0%) or supplemented with (1% or 2%) ethanol. Data represents Means ± SD.

| Parameters | Treatments | | |
| --- | --- | --- | --- |
|  | 0% ethanol | 1% ethanol | 2% ethanol |
| Number of detected spots | 611 ± 26 | 635 ± 23 | 616 ± 31 |
| Number of matched spots in replicate gels | 409 ± 38 | 426 ± 25 | 416 ± 25 |
| Number of matched spots in all gels | 138 | 138 | 138 |
