# Supplementary material for: Effect of Ethanol on Differential Protein Production and Expression of Potential Virulence Functions in the Opportunistic Pathogen Acinetobacter baumannii
Source: PLoS One. 2012 Dec 20;7(12):e51936. doi: 10.1371/journal.pone.0051936 (PMC3527336; doi:10.1371/journal.pone.0051936)
Supplement: Appendix S1 — Histograms showing average spot volumes of the 35 identified Acinetobacter baumannii ATCC 17978 total cell proteins that were differentially produced in response to low-level ethanol treatments. Bars with the same letter are not significantly different at P<0.05. (PDF) [file pone.0051936.s003.pdf]

Appendix S1. Histograms showing average spot volumes of the 35 identified *Acinetobacter baumannii* ATCC 17978 total cell proteins that were differentially produced in response to low-level ethanol treatments. Bars with the same letter are not significantly different at $P < 0.05$.

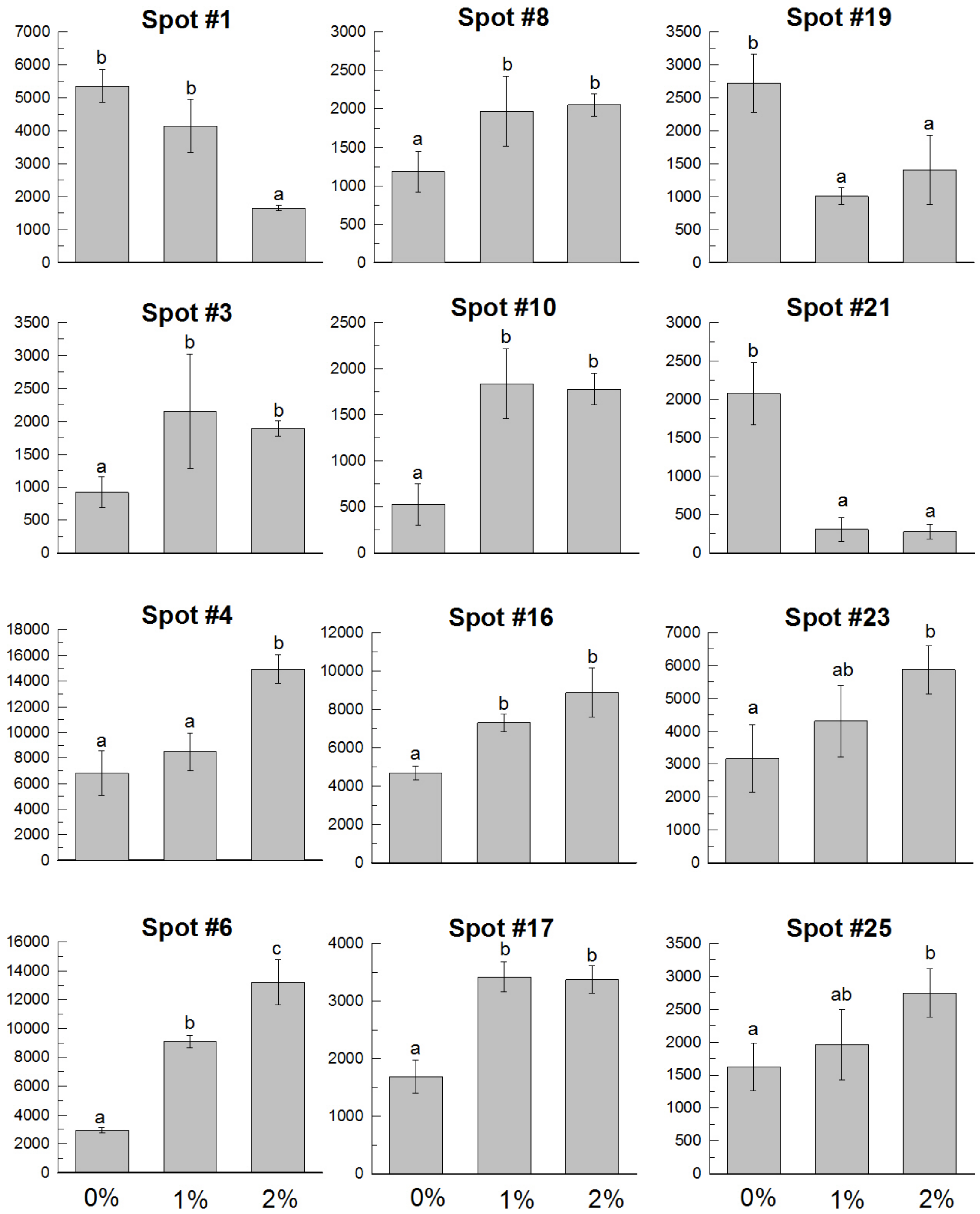
Spot #1
b
b
a
Spot #8
a
b
b
Spot #19
b
a
a
Spot #3
a
b
b
Spot #10
a
b
b
Spot #21
b
a
a
Spot #4
a
a
b
Spot #16
a
b
b
Spot #23
a
ab
b
Spot #6
a
b
c
Spot #17
a
b
b
Spot #25
a
ab
b
0%
1%
2%
0%
1%
2%
0%
1%
2%

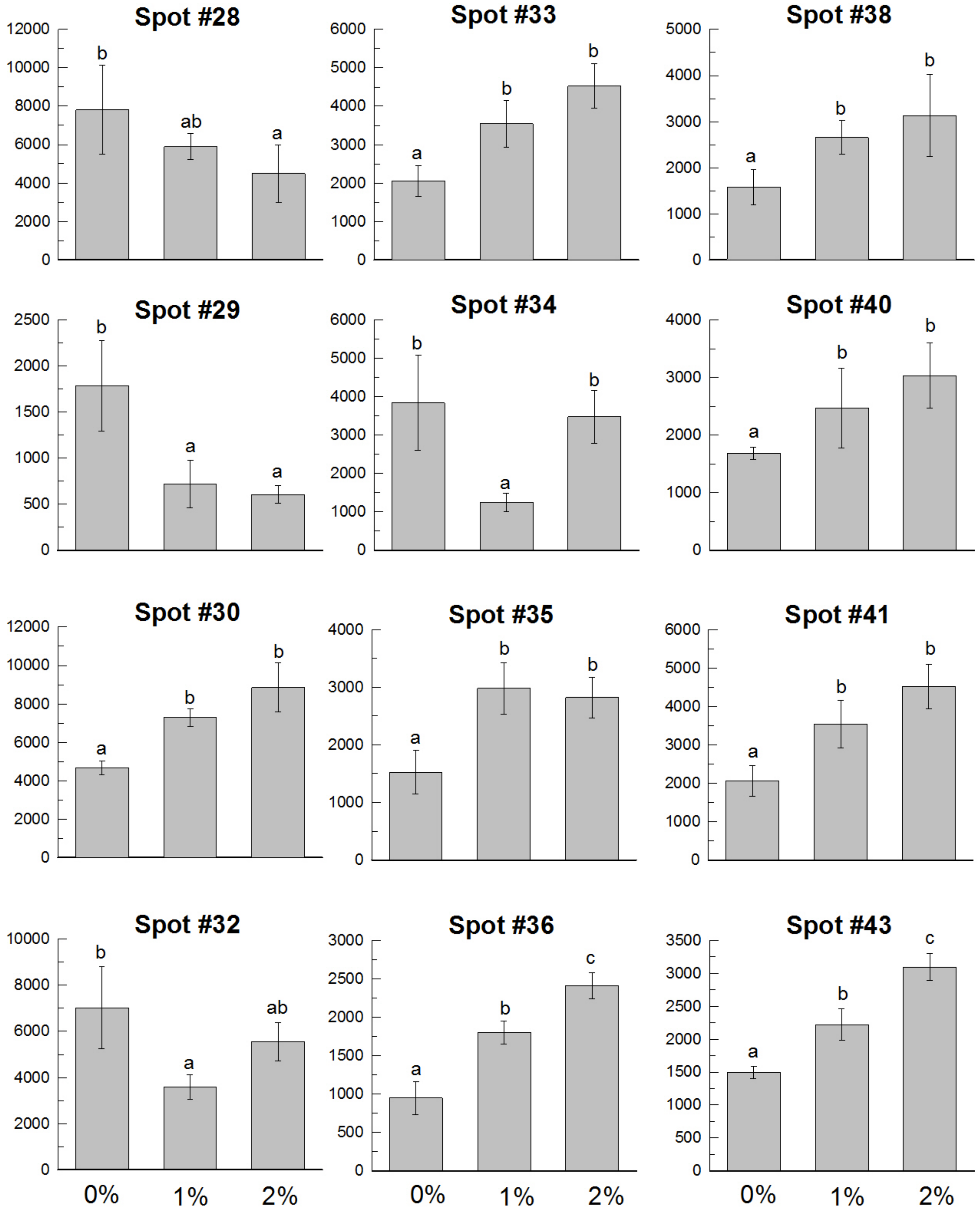
Spot #28
Spot #33
Spot #38
Spot #29
Spot #34
Spot #40
Spot #30
Spot #35
Spot #41
Spot #32
Spot #36
Spot #43
0%
1%
2%

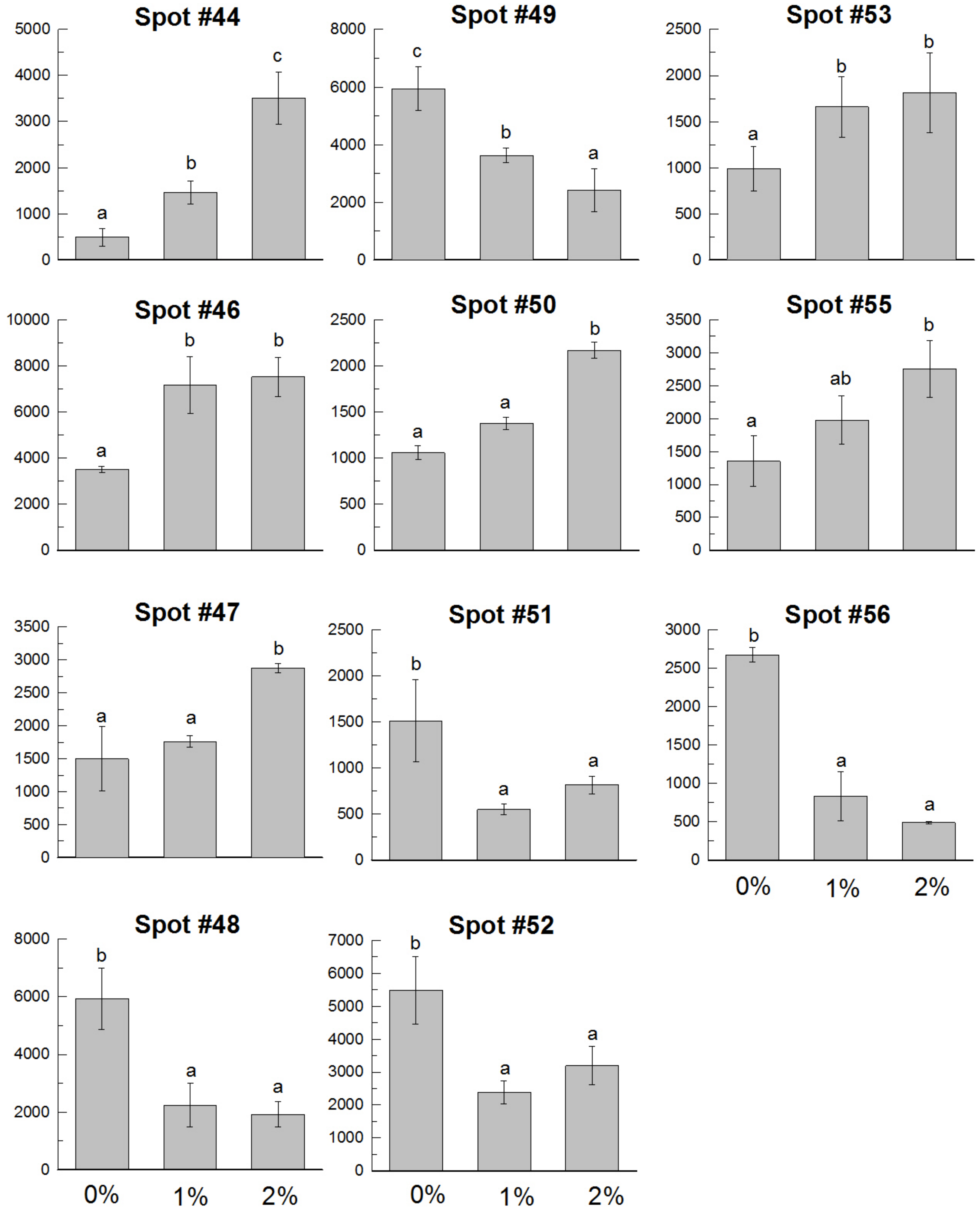

Spot #44
Spot #49
Spot #53
Spot #46
Spot #50
Spot #55
Spot #47
Spot #51
Spot #56
Spot #48
Spot #52
0%
1%
2%
