# Supplementary material for: Effect of Ethanol on Differential Protein Production and Expression of Potential Virulence Functions in the Opportunistic Pathogen Acinetobacter baumannii
Source: PLoS One. 2012 Dec 20;7(12):e51936. doi: 10.1371/journal.pone.0051936 (PMC3527336; doi:10.1371/journal.pone.0051936)
Supplement: Appendix S2 — List of the 35 identified Acinetobacter baumannii ATCC 17978 total cell proteins that were differentially produced in response to low-level ethanol treatments showing the Mascot match results and the sequences of matched peptides. (PDF) [file pone.0051936.s004.pdf]

Appendix S2. List of the 35 identified *Acinetobacter baumannii* ATCC 17978 total cell proteins that were differentially produced in response to low-level ethanol treatments showing the Mascot match results and the sequences of matched peptides.

1.

Match to: **gi|126642864** Score: **249** Expect: **8.4e-19** **Decoy: 71**
outer membrane protein A [Acinetobacter baumannii ATCC 17978]

Nominal mass (M_r): **37342**; Calculated pI value: **5.13**
NCBI BLAST search of gi|126642864 against nr
Unformatted sequence string for pasting into other applications

Taxonomy: Acinetobacter baumannii ATCC 17978

Fixed modifications: Carbamidomethyl (C)
Variable modifications: Oxidation (M)
Cleavage by Trypsin: cuts C-term side of KR unless next residue is P
Number of mass values searched: **17**
Number of mass values matched: **15**
Sequence Coverage: **42%**

Matched peptides shown in **Bold Red**

1 MLVAAPLAAA NAGVTVTPLL LGYTFQDSQH NNGGKDGNLT NGPELQDDLF
51 VGAALGIELT PWLGFEAEYN QVKGDVDGAS AGAEYKQK**QI** **NGNFYVTSDL**
101 **ITK**NYDSK**IK** **PYVLLGAGHY** **KYDFDGVNR**G TR**GTSEEGTL** **GNAGVGAFWR**
151 **LNDALSLR**TE ARATYNADEE FWNYTALAGL NVVLGGHLKP AAPVVEVAPV
201 EPTPVTPQPQ ELTEDLNMEL R**VFFDTNKSN** **IKDQYKPEIA** **K**VAEK**LSEYP**
251 **NATARIEGHT** **DNTGPRKLNE** **R**LSLARANSV K**SALVNEYNV** **DASRLSTQGF**
301 **AWDQPIADNK** TKEGRAMNR**R** **VFATITGSR**T VVVQPGQEAA APAAAQ

Start - End	Observed	Mr(expt)	Mr(calc)	ppm	Miss	Sequence
89 - 103	1712.8613	1711.8540	1711.8781	-14	0	K.QINGNFYVTSDLITK.N
109 - 121	1458.8419	1457.8346	1457.8394	-3	0	K.IKPYVLLGAGHYK.Y
122 - 129	985.4726	984.4653	984.4301	36	0	K.YDFDGVNR.G
133 - 150	1808.8376	1807.8303	1807.8489	-10	0	R.GTSEEGTLGNAGVGAFWR.L
151 - 158	901.5501	900.5428	900.5029	44	0	R.LNDALSLR.T
222 - 228	870.4861	869.4788	869.4283	58	0	R.VFFDTNK.S
229 - 241	1533.8155	1532.8082	1532.8198	-8	1	K.SNIKDQYKPEIAK.V
246 - 255	1121.5855	1120.5782	1120.5513	24	0	K.LSEYPNATAR.I
246 - 266	2299.1893	2298.1820	2298.0989	36	1	K.LSEYPNATARIEGHTDNTGPR.K
256 - 266	1196.5843	1195.5770	1195.5582	16	0	R.IEGHTDNTGPR.K
267 - 271	659.4170	658.4097	658.3762	51	1	R.KLNER.L
282 - 294	1437.6912	1436.6839	1436.6895	-4	0	K.SALVNEYNVDASR.L
295 - 310	1790.8521	1789.8448	1789.8635	-10	0	R.LSTQGFAWDQPIADNK.T
320 - 329	1107.6535	1106.6462	1106.6196	24	1	R.RVFATITGSR.T
321 - 329	951.5635	950.5562	950.5185	40	0	R.VFATITGSR.T

No match to: 1480.8169, 2446.2788

3.

Match to: **gi|162286757** Score: **296** Expect: **1.7e-23** **Decoy: 68**
F0F1 ATP synthase subunit alpha [Acinetobacter baumannii ATCC 17978]

Nominal mass (M_r): **55534**; Calculated pI value: **5.29**
NCBI BLAST search of gi|162286757 against nr
Unformatted sequence string for pasting into other applications

Taxonomy: Acinetobacter baumannii ATCC 17978
Links to retrieve other entries containing this sequence from NCBI Entrez:
gi|213987856 from Acinetobacter baumannii AB307-0294

Fixed modifications: Carbamidomethyl (C)
Variable modifications: Oxidation (M)
Cleavage by Trypsin: cuts C-term side of KR unless next residue is P
Number of mass values searched: **22**
Number of mass values matched: **21**
Sequence Coverage: **34%**

Matched peptides shown in **Bold Red**

1 **MQQLNPSEIS ALIK**QRIGDL DTSATAK**NEG TIVMVSDGIV R**IHGLADAMY
51 GEMIEFDGGL FGMALNLEQD SVGAVVLGNY LSLQEGQKAR CTGR**VLEVPV**
101 **GPELLGR**VVD ALGNPIDGKG PIDAKLTDAV EK**VAPGVIWR** QSVDQPVQTG
151 YK**SVDTMIPV GRGQR**ELIIG DRQTGKTAMA IDAIIAQKNS GIK**CVYVAIG**
201 **QKQSTIANVV R**KLEETGAMA YTTVVAAAAA DPAAMQYLAP YSGCTMGEYF
251 R**DRGEDALII YDDLSK**QAVA YR**QISLLLR**R PPGR**EAYPGD VFYLHSR**LLE
301 RASRVSAEYV EKFTNGAVTG KTGSLTALPI IETQAGDVSA FVPTNVISIT
351 DGQIFLETSL FNAGIRPAVN AGISVSRVGG SAQTKIIKKL SGGIR**TALAQ**
401 **YRELAAFAQF ASDLDEATRK QLEHGQR**VTE LMKQKQYAPY SIADQAVSVY
451 ASNEGYMADV EVK**KIVDFDA ALIAYFR**SEY APLMK**QIDET GDYNKDIEAA**
501 **IK**AGIESFKA TQTY

Start - End	Observed	Mr(expt)	Mr(calc)	ppm	Miss	Sequence	
1 - 14	1587.8209	1586.8136	1586.8338	-13	0	-.MQQLNPSEISALIK.Q	Oxidation (M)
28 - 41	1489.7529	1488.7456	1488.7606	-10	0	K.NEGTIVMVSDGIVR.I	
28 - 41	1505.7494	1504.7421	1504.7556	-9	0	K.NEGTIVMVSDGIVR.I	Oxidation (M)
95 - 107	1377.8048	1376.7975	1376.8028	-4	0	R.VLEVPVGPELLGR.V	
133 - 140	897.5731	896.5658	896.5232	48	0	K.VAPGVIWR.Q	
153 - 162	1074.5877	1073.5804	1073.5539	25	0	K.SVDTMIPVGR.G	
153 - 162	1090.5816	1089.5743	1089.5489	23	0	K.SVDTMIPVGR.G	Oxidation (M)
153 - 165	1415.7209	1414.7136	1414.7351	-15	1	K.SVDTMIPVGRGQR.E	
194 - 202	1037.5709	1036.5636	1036.5376	25	0	K.CVYVAIGQK.Q	
203 - 211	987.5915	986.5842	986.5509	34	0	K.QSTIANVVR.K	
252 - 266	1722.8296	1721.8223	1721.8472	-14	1	R.DRGEDALIIYDDLSK.Q	
273 - 279	842.5858	841.5785	841.5385	48	0	R.QISLLLR.R	
285 - 297	1553.7211	1552.7138	1552.7310	-11	0	R.EAYPGDVFYLHSR.L	

396 - 402	822.4941	821.4868	821.4395	58	0	R.TALAQYR.E
403 - 419	1854.8629	1853.8556	1853.8795	-13	0	R.ELAAFAQFASDLDEATR.K
403 - 420	1982.9616	1981.9543	1981.9745	-10	1	R.ELAAFAQFASDLDEATRK.Q
420 - 427	995.5705	994.5632	994.5308	33	1	R.KQLEHGQR.V
421 - 427	867.4876	866.4803	866.4359	51	0	K.QLEHGQR.V
464 - 477	1641.8750	1640.8677	1640.8926	-15	1	K.KIVDFDAALIAYFR.S
465 - 477	1513.7881	1512.7808	1512.7977	-11	0	K.IVDFDAALIAYFR.S
486 - 502	1922.9066	1921.8993	1921.9269	-14	1	K.QIDETGDYNKDIEAAIK.A

No match to: 1529.7770

4.
Match to: **gi|301347500** Score: **456** Expect: **1.7e-39 Decoy: 74**
chaperonin GroEL [Acinetobacter baumannii AB056]

Nominal mass (M_r): **54465**; Calculated pI value: **5.05**
NCBI BLAST search of gi|301347500 against nr
Unformatted sequence string for pasting into other applications

Taxonomy: Acinetobacter baumannii AB056

Fixed modifications: Carbamidomethyl (C)
Variable modifications: Oxidation (M)
Cleavage by Trypsin: cuts C-term side of KR unless next residue is P
Number of mass values searched: **43**
Number of mass values matched: **38**
Sequence Coverage: **61%**

Matched peptides shown in **Bold Red**

1 MSAKDVK**FGD SAR**SK**MIAGV NVLADAVK**VT LGPKGRNVVI DR**SFGAPHIT**
51 **K**DGVTVAKEI SLK**DKFENMG AQLVR**EVSSK **TNDIAGDGTT TATVLAQAIL**
101 **NEGIKSVTAG MNPMDLKRGI DIAVKTVVEN IR**SIAKPADD FKAIEQVGSI
151 SANSDTTVGK LIAQAMEKVG K**EGVITVEEG SGFEDALDVV EGMQFDRGYI**
201 **SPYFANKQDT LTAELENPFI LLVDKKISNI RELISVLEAV AKTGKPLLII**
251 **AEDVEGEALA TLVVNNMR**GI IKVCAVK**APG FGDRR**KAMLQ DIAILTGATV
301 ISEEVGMSLE QATLQDLGTA HK**ITVSKENT VIVDGAGDAA AIAERVQQIR**
351 **AQIEESTSEY DREK**LQERVA KLAGGVAVIK IGAATEVEMK EKK**DRVDDAL**
401 **HATRAAVEEG VVAGGGVALV RAVNALEGLK GANEDQTAGI NILRRAIEAP**
451 **LRQIVANAGD EPSVVINAVK** NGEGNFGYNA ATGEYGDMLE MGILDPAKVT
501 RSALEHAASV AGLMLTT

Start - End	Observed	Mr(expt)	Mr(calc)	ppm	Miss	Sequence
8 - 13	652.3633	651.3560	651.2976	90	0	K.FGDSAR.S
16 - 28	1300.7296	1299.7223	1299.7220	0	0	K.MIAGVNVLADAVK.V
16 - 28	1316.7310	1315.7237	1315.7170	5	0	K.MIAGVNVLADAVK.V Oxidation (M)
43 - 51	957.5537	956.5464	956.5080	40	0	R.SFGAPHITK.D
64 - 75	1407.7000	1406.6927	1406.6976	-3	1	K.DKFENMGAQLVR.E
64 - 75	1423.6981	1422.6908	1422.6925	-1	1	K.DKFENMGAQLVR.E Oxidation (M)
66 - 75	1164.5972	1163.5899	1163.5757	12	0	K.FENMGAQLVR.E
81 - 105	2487.3043	2486.2970	2486.2864	4	0	K.TNDIAGDGTTTATVLAQAILNEGIK.S
106 - 118	1419.7011	1418.6938	1418.7010	-5	1	K.SVTAGMNPMDLKR.G
106 - 118	1435.6974	1434.6901	1434.6959	-4	1	K.SVTAGMNPMDLKR.G Oxidation (M)
106 - 118	1451.6928	1450.6855	1450.6908	-4	1	K.SVTAGMNPMDLKR.G 2 Oxidation (M)
119 - 125	715.4746	714.4673	714.4276	56	0	R.GIDIAVK.T
126 - 132	830.5355	829.5282	829.4658	75	0	K.TVVENIR.S
172 - 197	2828.3970	2827.3897	2827.2858	37	0	K.EGVITVEEGSGFEDALDVVEGMQFDR.G
172 - 197	2844.4019	2843.3946	2843.2808	40	0	K.EGVITVEEGSGFEDALDVVEGMQFDR.G Oxidation (M)
198 - 207	1159.6106	1158.6033	1158.5709	28	0	R.GYISPYFANK.Q
208 - 225	2059.0556	2058.0483	2058.0885	-20	0	K.QDTLTAELENPFILLVDK.K
208 - 226	2187.1653	2186.1580	2186.1834	-12	1	K.QDTLTAELENPFILLVDKK.I
227 - 231	602.4177	601.4104	601.3547	93	0	K.ISNIR.E
232 - 242	1171.7192	1170.7119	1170.6860	22	0	R.ELISVLEAVAK.T
243 - 268	2766.5853	2765.5780	2765.4997	28	0	K.TGKPLLIIAEDVEGEALATLVVNNMR.G
243 - 268	2782.5805	2781.5732	2781.4946	28	0	K.TGKPLLIIAEDVEGEALATLVVNNMR.G Oxidation (M)
278 - 284	719.4091	718.4018	718.3398	86	0	K.APGFGDR.R
278 - 285	875.5078	874.5005	874.4409	68	1	K.APGFGDRR.K
323 - 345	2300.1844	2299.1771	2299.2019	-11	1	K.ITVSKENTVIVDGAGDAAAIAER.V
328 - 345	1771.8503	1770.8430	1770.8748	-18	0	K.ENTVIVDGAGDAAAIAER.V
346 - 350	643.4499	642.4426	642.3813	95	0	R.VQQIR.A
351 - 364	1684.7383	1683.7310	1683.7587	-16	1	R.AQIEESTSEYDREK.L
394 - 404	1268.6494	1267.6421	1267.6269	12	1	K.DRVDDALHATR.A
396 - 404	997.5440	996.5367	996.4989	38	0	R.VDDALHATR.A
405 - 421	1553.8461	1552.8388	1552.8573	-12	0	R.AAVEEGVVAGGGVALVR.A
422 - 444	2367.2512	2366.2439	2366.2553	-5	1	R.AVNALEGLKGANEDQTAGINILR.R
431 - 444	1471.7386	1470.7313	1470.7426	-8	0	K.GANEDQTAGINILR.R
431 - 445	1627.8347	1626.8274	1626.8438	-10	1	K.GANEDQTAGINILRR.A
445 - 452	925.6114	924.6041	924.5505	58	1	R.RAIEAPLR.Q
446 - 452	769.5204	768.5131	768.4494	83	0	R.AIEAPLR.Q
446 - 470	2574.4480	2573.4407	2573.4177	9	1	R.AIEAPLRQIVANAGDEPSVVINAVK.N
453 - 470	1823.9520	1822.9447	1822.9789	-19	0	R.QIVANAGDEPSVVINAVK.N

No match to: 1054.5975, 1073.6238, 1367.1952, 1978.0093, 2787.3217

6.

Match to: **gi|184157742** Score: **116** Expect: **1.7e-05 Decoy: 52**
putative 17 kDa surface antigen [Acinetobacter baumannii ACICU]

Nominal mass (M_r): **12424**; Calculated pI value: **4.70**
NCBI BLAST search of gi|184157742 against nr
Unformatted sequence string for pasting into other applications

Taxonomy: Acinetobacter baumannii ACICU
Links to retrieve other entries containing this sequence from NCBI Entrez:
gi|294840522 from Acinetobacter baumannii 6014059
gi|183209336 from Acinetobacter baumannii ACICU

Fixed modifications: Carbamidomethyl (C)
Variable modifications: Oxidation (M)
Cleavage by Trypsin: cuts C-term side of KR unless next residue is P
Number of mass values searched: **12**
Number of mass values matched: **7**
Sequence Coverage: **77%**

Matched peptides shown in **Bold Red**

1 M**TDQNTDHAQ LVAGDHNYSD SR**WR**ESYLTR PYYQEAQLTT PDLDYDRDFS**
51 **AAYELGHRAR** SESK**EGTQFE DMEGSLQQKW EELKAESR**LK WEHAKQAIKD
101 AWDDI

Start - End	Observed	Mr(expt)	Mr(calc)	ppm	Miss	Sequence
2 - 22	2344.0417	2343.0344	2343.0112	10	0	M.TDQNTDHAQLVAGDHNYSDSR.W
25 - 47	2837.4646	2836.4573	2836.3191	49	0	R.ESYLTRPYYQEAQLTTPDLDYDR.D
48 - 58	1265.6089	1264.6016	1264.5836	14	0	R.DFSAAYELGHR.A
48 - 60	1492.7302	1491.7229	1491.7218	1	1	R.DFSAAYELGHRAR.S
65 - 79	1726.7367	1725.7294	1725.7516	-13	0	K.EGTQFEDMEGSLQQK.W
65 - 79	1742.7358	1741.7285	1741.7465	-10	0	K.EGTQFEDMEGSLQQK.W Oxidation (M)
80 - 88	1147.6044	1146.5971	1146.5669	26	1	K.WEELKAESR.L

No match to: 911.5594, 1173.0376, 1316.6401, 1471.7472, 1553.8566

8.

Match to: **gi|169797643** Score: **131** Expect: **5.4e-07 Decoy: 83**
F0F1 ATP synthase subunit epsilon [Acinetobacter baumannii AYE]

Nominal mass (M_r): **14587**; Calculated pI value: **4.83**
NCBI BLAST search of gi|169797643 against nr
Unformatted sequence string for pasting into other applications

Taxonomy: Acinetobacter baumannii AYE
Links to retrieve other entries containing this sequence from NCBI Entrez:
gi|184156499 from Acinetobacter baumannii ACICU
gi|226740702 from Acinetobacter baumannii ATCC 17978
gi|260408759 from Acinetobacter baumannii ATCC 19606

Fixed modifications: Carbamidomethyl (C)
Variable modifications: Oxidation (M)
Cleavage by Trypsin: cuts C-term side of KR unless next residue is P
Number of mass values searched: **11**
Number of mass values matched: **8**
Sequence Coverage: **62%**

Matched peptides shown in **Bold Red**

```
  1 MATMQCDVVS VKESIYSGAV TMLIAKGAGG ELGILPGHAP LVTLLQPGPI
 51 RVLLENGTEE IVYVSGGVLE VQPHVVTVLA DTAIRADNLD EAAILEARKN
101 AEQLLANQKS DLDSAAALAA LAETAAQLET IRKIKNRAQ
```

Start - End	Observed	Mr(expt)	Mr(calc)	ppm	Miss	Sequence
13 - 26	1482.7593	1481.7520	1481.7799	-19	0	K.ESIYSGAVTMLIAK.G
13 - 26	1498.7605	1497.7532	1497.7749	-14	0	K.ESIYSGAVTMLIAK.G Oxidation (M)
27 - 51	2433.4008	2432.3935	2432.3904	1	0	K.GAGGELGILPGHAPLVTLLQPGPIR.V
86 - 98	1400.6893	1399.6820	1399.6943	-9	0	R.ADNLDEAAILEAR.K
99 - 109	1256.6953	1255.6880	1255.6884	-0	1	R.KNAEQLLANQK.S
100 - 109	1128.6069	1127.5996	1127.5934	5	0	K.NAEQLLANQK.S
110 - 132	2301.1851	2300.1778	2300.1859	-4	0	K.SDLDSAAALAALAETAAQLETIR.K
110 - 133	2429.3059	2428.2986	2428.2809	7	1	K.SDLDSAAALAALAETAAQLETIRK.I

No match to: 1038.5952, 1175.7592, 1186.6234

10.

Match to: **gi|126642383** Score: **132** Expect: **4.3e-07 Decoy: 62**
superoxide dismutase [Acinetobacter baumannii ATCC 17978]

Nominal mass (M_r): **24207**; Calculated pI value: **5.88**
NCBI BLAST search of gi|126642383 against nr
Unformatted sequence string for pasting into other applications

Taxonomy: Acinetobacter baumannii ATCC 17978

Fixed modifications: Carbamidomethyl (C)
Variable modifications: Oxidation (M)
Cleavage by Trypsin: cuts C-term side of KR unless next residue is P
Number of mass values searched: **11**
Number of mass values matched: **8**
Sequence Coverage: **31%**

Matched peptides shown in **Bold Red**

```
  1 MYNPANARNR NMTTITLPAL PYGYDDLAPH ISKETLEYHH DKHHNTYVVN
 51 LNNLIKGTDL EGKTLEEIIK ATAGDASKAG IFNNAAQVWN HTFYWNSMKP
101 NGGGKPTGAI AAKIDEAFGS YEKFAEEFTA AATTQFGSGW AWLVADEVNG
151 KLSITKTSNA DTPLAHGQIA VLTIDVWEHA YYIDFRNLRP KYIATFLENL
201 VNWDYANAKL AGQPAGVEK
```

Start - End	Observed	Mr(expt)	Mr(calc)	ppm	Miss	Sequence
23 - 31	1171.5400	1170.5327	1170.5305	2	0	K.ETLEYHHDK.H
23 - 45	2831.5548	2830.5475	2830.4150	47	1	K.ETLEYHHDKHHNTYVVNLNNLIK.G
32 - 45	1678.8543	1677.8470	1677.8951	-29	0	K.HHNTYVVNLNNLIK.G
46 - 59	1545.8034	1544.7961	1544.8297	-22	1	K.GTDLEGKTLEEIIK.A
53 - 59	845.5160	844.5087	844.4906	22	0	K.TLEEIIK.A
176 - 180	627.4329	626.4256	626.3864	63	0	R.NLRPK.Y
181 - 198	2145.0317	2144.0244	2144.0578	-16	0	K.YIATFLENLVNWDYANAK.L
199 - 208	969.5535	968.5462	968.5291	18	0	K.LAGQPAGVEK.-

No match to: 1173.5015, 1225.3957, 1563.4804

16.

Match to: **gi|126640260** Score: **252** Expect: **4.3e-19** **Decoy: 70**
glutathione peroxidase [Acinetobacter baumannii ATCC 17978]

Nominal mass (M_r): **20252**; Calculated pI value: **4.88**
NCBI BLAST search of gi|126640260 against nr
Unformatted sequence string for pasting into other applications

Taxonomy: Acinetobacter baumannii ATCC 17978
Links to retrieve other entries containing this sequence from NCBI Entrez:
gi|184156502 from Acinetobacter baumannii ACICU

Fixed modifications: Carbamidomethyl (C)
Variable modifications: Oxidation (M)
Cleavage by Trypsin: cuts C-term side of KR unless next residue is P
Number of mass values searched: **17**
Number of mass values matched: **14**
Sequence Coverage: **62%**

Matched peptides shown in **Bold Red**

```
  1 MTQSVYHIPV KAISGETVDL DQYKGKVLLI VNTASKCGLT PQYEGLEKLY
 51 QAKKDQGLEI LGFPANNFKE QEPGSDEEIQ QFCSLNYDVH FPLFSKISVA
101 GEDKHPLYQA LTTAQPERIG EGPFRERLEG LGIPTNPAPE VLWNFEKFLV
151 NKNGEVVARF APNLTADDEQ IVKAVEAELA K
```

Start - End	Observed	Mr(expt)	Mr(calc)	ppm	Miss	Sequence
1 - 11	1302.6814	1301.6741	1301.6802	-5	0	-.MTQSVYHIPVK.A
2 - 11	1171.6383	1170.6310	1170.6397	-7	0	M.TQSVYHIPVK.A
12 - 24	1438.6601	1437.6528	1437.6987	-32	0	K.AISGETVDLDQYK.G
27 - 36	1057.6645	1056.6572	1056.6543	3	0	K.VLLIVNTASK.C
37 - 48	1394.6258	1393.6185	1393.6548	-26	0	K.CGLTPQYEGLEK.L
54 - 69	1790.8715	1789.8642	1789.9363	-40	1	K.KDQGLEILGFPANNFK.E
55 - 69	1662.7806	1661.7733	1661.8413	-41	0	K.DQGLEILGFPANNFK.E
97 - 118	2424.2273	2423.2200	2423.2445	-10	1	K.ISVAGEDKHPLYQALTTAQPER.I
105 - 118	1624.7864	1623.7791	1623.8369	-36	0	K.HPLYQALTTAQPER.I
119 - 125	775.4450	774.4377	774.4024	46	0	R.IGEGPFR.E
119 - 127	1060.5609	1059.5536	1059.5461	7	1	R.IGEGPFRER.L
153 - 159	744.4404	743.4331	743.3926	55	0	K.NGEVVAR.F
153 - 173	2286.1317	2285.1244	2285.1652	-18	1	K.NGEVVARFAPNLTADDEQIVK.A
160 - 173	1560.7326	1559.7253	1559.7831	-37	0	R.FAPNLTADDEQIVK.A

No match to: 1141.6222, 1193.6139, 1448.7821

17.

Match to: **gi|126641253** Score: **199** Expect: **8.5e-14 Decoy: 68**
alkyl hydroperoxide reductase C22 subunit [Acinetobacter baumannii ATCC 17978]

Nominal mass (M_r): **18316**; Calculated pI value: **4.96**
NCBI BLAST search of gi|126641253 against nr
Unformatted sequence string for pasting into other applications

Taxonomy: Acinetobacter baumannii ATCC 17978

Fixed modifications: Carbamidomethyl (C)
Variable modifications: Oxidation (M)
Cleavage by Trypsin: cuts C-term side of KR unless next residue is P
Number of mass values searched: **14**
Number of mass values matched: **11**
Sequence Coverage: **72%**

Matched peptides shown in **Bold Red**

```
  1 MNETNLKGKW SVVFFYPADF TFVCPTELED LADNYAEFQK LGVEIYGVST
 51 DTHFTHKAWH DTSDAIKKIQ YPLIGDPTWT LSKNFDVLIE SEGLADRGTF
101 VIDPEGKIQI VEINAGGIGR DASELLRKVK AAQYVHSHPG EVCPAKWKEG
151 EATLAPSIDL VGKI
```

Start - End	Observed	Mr(expt)	Mr(calc)	ppm	Miss	Sequence
41 - 57	1903.9031	1902.8958	1902.9476	-27	0	K.LGVEIYGVSTDTHFTHK.A
58 - 67	1143.5468	1142.5395	1142.5356	3	0	K.AWHDTSDAIK.K
58 - 68	1271.6267	1270.6194	1270.6306	-9	1	K.AWHDTSDAIKK.I
68 - 83	1859.9675	1858.9602	1859.0193	-32	1	K.KIQYPLIGDPTWTLSK.N
69 - 83	1731.8778	1730.8705	1730.9243	-31	0	K.IQYPLIGDPTWTLSK.N
84 - 97	1577.7397	1576.7324	1576.7733	-26	0	K.NFDVLIESEGLADR.G
98 - 107	1062.5618	1061.5545	1061.5393	14	0	R.GTFVIDPEGK.I
108 - 120	1339.7506	1338.7433	1338.7619	-14	0	K.IQIVEINAGGIGR.D
121 - 127	803.4590	802.4517	802.4185	41	0	R.DASELLR.K
131 - 146	1750.7822	1749.7749	1749.8257	-29	0	K.AAQYVHSHPGEVCPAK.W
149 - 164	1612.8285	1611.8212	1611.8719	-31	1	K.EGEATLAPSIDLVGKI.-

No match to: 1227.5939, 1361.7275, 1448.7899

19.

Match to: **gi|126640806** Score: **255** Expect: **2.1e-19**
NarL family two-component response regulator [Acinetobacter baumannii ATCC 17978]

Nominal mass (M_r): **27176**; Calculated pI value: **4.98**
NCBI BLAST search of gi|50083946 against nr
Unformatted sequence string for pasting into other applications

Taxonomy: Acinetobacter sp. ADP1
Links to retrieve other entries containing this sequence from NCBI Entrez:
gi|260410557 from Acinetobacter baumannii ATCC 19606

Fixed modifications: Carbamidomethyl (C)
Variable modifications: Oxidation (M)
Cleavage by Trypsin: cuts C-term side of KR unless next residue is P
Number of mass values searched: **17**
Number of mass values matched: **16**
Sequence Coverage: **68%**

Matched peptides shown in **Bold Red**

```
  1 MSQEEKLPKI LIVEDDERLA RLTQEYLIRN GLEVGVETDG NRAIRRIISE
 51 QPDLVVLDVM LPGADGLTVC REVRPHYHQP ILMLTARTED MDQVLGLEMG
101 ADDYVAKPVQ PRVLLARIRA LLRRTDKTVE DEVAQRIEFD DLVIDNGGRS
151 VTLNGELVDF TSAEYDLLWL LASNAGRILS REDIFERLRG IEYDGQDRSI
201 DVRISRIRPK IGDDPENPKR IKTVRSKGYL FVKETNGL
```

Start - End	Observed	Mr(expt)	Mr(calc)	ppm	Miss	Sequence
10 - 18	1101.5886	1100.5813	1100.5713	9	0	K.ILIVEDDER.L
22 - 29	1035.6020	1034.5947	1034.5760	18	0	R.LTQEYLIR.N
30 - 42	1359.6216	1358.6143	1358.6426	-21	0	R.NGLEVGVETDGNR.A
47 - 71	2726.5585	2725.5512	2725.4031	54	0	R.IISEQPDLVVLDVMLPGADGLTVCR.E Oxidation (M)
72 - 87	1961.0900	1960.0827	1960.0465	18	0	R.EVRPHYHQPILMLTAR.T
72 - 87	1977.0953	1976.0880	1976.0414	24	0	R.EVRPHYHQPILMLTAR.T Oxidation (M)
88 - 112	2809.4319	2808.4246	2808.2946	46	0	R.TEDMDQVLGLEMGADDYVAKPVQPR.V 2 Oxidation (M)
120 - 124	628.4748	627.4675	627.4180	79	1	R.ALLRR.T
125 - 136	1390.6381	1389.6308	1389.6736	-31	1	R.TDKTVEDEVAQR.I
137 - 149	1462.6596	1461.6523	1461.7100	-39	0	R.IEFDDLVIDNGGR.S
178 - 187	1277.7003	1276.6930	1276.6775	12	1	R.ILSREDIFER.L
182 - 187	808.4329	807.4256	807.3763	61	0	R.EDIFER.L
190 - 198	1052.4876	1051.4803	1051.4570	22	0	R.GIEYDGQDR.S
211 - 220	1140.5684	1139.5611	1139.5571	4	1	K.IGDDPENPKR.I
226 - 233	941.5789	940.5716	940.5382	36	1	R.SKGYLFVK.E
228 - 233	726.4738	725.4665	725.4112	76	0	K.GYLFVK.E

No match to: 1790.7588

21.

Match to: **gi|193077015** Score: **275** Expect: **2.1e-21** **Decoy: 72**
Phenylacetate-CoA oxygenase/reductase PaaK subunit [Acinetobacter baumannii ATCC 17978]

Nominal mass (M_r): **40276**; Calculated pI value: **5.03**
NCBI BLAST search of gi|193077015 against nr
Unformatted sequence string for pasting into other applications

Taxonomy: Acinetobacter baumannii ATCC 17978

Fixed modifications: Carbamidomethyl (C)
Variable modifications: Oxidation (M)
Cleavage by Trypsin: cuts C-term side of KR unless next residue is P
Number of mass values searched: **18**
Number of mass values matched: **17**
Sequence Coverage: **44%**

Matched peptides shown in **Bold Red**

```
  1 MSQFVPLKVK SITPQTDQAI CIAFDVVPEQ QEQFQFQPGQ HLTIRHLTEA
 51 GEIRRCYSIC SYAGKEDISI AVKKIDQGQF SNWANDHLKV GDVLEVMPPQ
101 GVFFQKAAKM GGQNYLGVAA GSGITPILSI IKQVLFEQPE ANFTLLYGNR
151 SWKQTMFAEQ IMDLKDQFKE RFQLINIFSR EFNDSELMNG RIDAEKLKQL
201 FDYEVLETNF DHVFACGPDE MMNAVENTLP NFGIAKERIH TERFHTGQAR
251 KRSVEADANR KEEKVNIILD GRELIVSVAQ DDESILDAAL RAGADLPYAC
301 KGGVCATCRC KVLSGEVDMF LNYSLEEDEV EKGYVLSCQT LPKGSNVRLS
351 FDE
```

Start - End	Observed	Mr(expt)	Mr(calc)	ppm	Miss	Sequence
1 - 8	965.5137	964.5064	964.5052	1	0	-.MSQFVPLK.V Oxidation (M)
2 - 10	1045.5864	1044.5791	1044.6332	-52	1	M.SQFVPLKVK.S
46 - 54	1025.5587	1024.5514	1024.5301	21	0	R.HLTEAGEIR.R
46 - 55	1181.6498	1180.6425	1180.6312	10	1	R.HLTEAGEIRR.C
56 - 73	2064.0796	2063.0723	2062.9703	49	1	R.CYSICSYAGKEDISIAVK.K
74 - 89	1900.8562	1899.8489	1899.9227	-39	1	K.KIDQGQFSNWANDHLK.V
75 - 89	1772.7630	1771.7557	1771.8278	-41	0	K.IDQGQFSNWANDHLK.V
133 - 150	2139.0284	2138.0211	2138.0796	-27	0	K.QVLFEQPEANFTLLYGNR.S
172 - 180	1137.6455	1136.6382	1136.6342	4	0	R.FQLINIFSR.E
181 - 191	1327.5653	1326.5580	1326.5510	5	0	R.EFNDSELMNGR.I Oxidation (M)
192 - 198	816.4482	815.4409	815.4752	-42	1	R.IDAEKLK.Q
239 - 243	655.3974	654.3901	654.3449	69	0	R.IHTER.F
253 - 261	989.5606	988.5533	988.4937	60	1	R.SVEADANRK.E
265 - 272	899.5622	898.5549	898.5236	35	0	K.VNIILDGR.E
273 - 291	2057.0167	2056.0094	2056.0688	-29	0	R.ELIVSVAQDDESILDAALR.A
292 - 301	1065.5120	1064.5047	1064.4961	8	0	R.AGADLPYACK.G
302 - 309	880.4121	879.4048	879.3691	41	0	K.GGVCATCR.C

No match to: 1090.5588

22.

Match to: **gi|126641049** Score: **242** Expect: **4.3e-18 Decoy: 72**
putative signal peptide [Acinetobacter baumannii ATCC 17978]

Nominal mass (M_r): **52814**; Calculated pI value: **5.05**
NCBI BLAST search of gi|126641049 against nr
Unformatted sequence string for pasting into other applications

Taxonomy: Acinetobacter baumannii ATCC 17978
Links to retrieve other entries containing this sequence from NCBI Entrez:
gi|126386933 from Acinetobacter baumannii ATCC 17978

Fixed modifications: Carbamidomethyl (C)
Variable modifications: Oxidation (M)
Cleavage by Trypsin: cuts C-term side of KR unless next residue is P
Number of mass values searched: **21**
Number of mass values matched: **18**
Sequence Coverage: **46%**

Matched peptides shown in **Bold Red**

```
  1 MFLRKTLSIA LLATASSAVF AQGLVLNNDD LRTDLNWLNQ QGVINISTST
 51 WPLSGDEIQR ALSQAKVTHP AQQKVINSVL NALKADNNTV KVGAFAESDI
101 KNIPQAFGDN QKSQYQGSLE FNAGGENWDA KIRVNAEKDP QIDSGHDVNV
151 EGSYVAGKLW NQWIVAGQIP TWWGPGHDGS LIRGDASRPV YGVTAQRAVQ
201 NAFETKWLSW IGPWQYQAFA GQLDDYKAVP DAKLIGLRLT AQPLPYLELG
251 ASRTIQWGGD GRSESFSSLW DAIKGNDNVY GDTENPSNQL AGFDGRLLLQ
301 PLLNIPVSLY GQYVGEDEAG YLPSKKMYLA GVDYSSSYND MPYQLYAEWA
351 DTRTNGDVKS ISYTHSVYKD GYYQHGFPLG HAMGGDGQMY SVGGDIRFDV
401 MNRLSGRAMV VKVNQSNLAI NKAFPKDDEI KALDLTWTHY IKPDLPLKIN
451 GWVSDSDLEG NDAGASIGVE IPLERKMFGF
```

Start - End	Observed	Mr(expt)	Mr(calc)	ppm	Miss	Sequence
67 - 74	908.5252	907.5179	907.4876	33	0	K.VTHPAQQK.V
75 - 84	1070.6752	1069.6679	1069.6495	17	0	K.VINSVLNALK.A
92 - 101	1036.5560	1035.5487	1035.5237	24	0	K.VGAFAESDIK.N
102 - 112	1231.6210	1230.6137	1230.5993	12	0	K.NIPQAFGDNQK.S
113 - 131	2100.8746	2099.8673	2099.9184	-24	0	K.SQYQGSLEFNAGGENWDAK.I
134 - 158	2628.2890	2627.2817	2627.2463	13	1	R.VNAEKDPQIDSGHDVNVEGSYVAGK.L
184 - 197	1476.7216	1475.7143	1475.7481	-23	0	R.GDASRPVYGVTAQR.A
198 - 206	1007.5604	1006.5531	1006.5083	44	0	R.AVQNAFETK.W
239 - 253	1628.8469	1627.8396	1627.8933	-33	0	R.LTAQPLPYLELGASR.T
254 - 262	989.5093	988.5020	988.4727	30	0	R.TIQWGGDGR.S
263 - 274	1369.6377	1368.6304	1368.6561	-19	0	R.SESFSSLWDAIK.G
275 - 296	2339.9917	2338.9844	2339.0051	-9	0	K.GNDNVYGDTENPSNQLAGFDGR.L
360 - 369	1184.6038	1183.5965	1183.5873	8	0	K.SISYTHSVYK.D
398 - 403	781.3968	780.3895	780.3589	39	0	R.FDVMNR.L

398 - 403	797.3900	796.3827	796.3538	36	0	R.FDVMNR.L Oxidation (M)
423 - 431	1062.5729	1061.5656	1061.5393	25	1	K.AFPKDDEIK.A
449 - 475	2813.4566	2812.4493	2812.3515	35	0	K.INGWVSDSDLEGNDAGASIGVEIPLER.K
476 - 480	645.3530	644.3457	644.2992	72	1	R.KMFGF.- Oxidation (M)

No match to: 1239.6371, 1256.6627, 1286.6409

23.

Match to: **gi|162286755** Score: **370** Expect: **6.8e-31 Decoy: 72**

F0F1 ATP synthase subunit beta [Acinetobacter baumannii ATCC 17978]

Nominal mass (M_r): **50300**; Calculated pI value: **5.03**

NCBI BLAST search of gi|162286755 against nr

Unformatted sequence string for pasting into other applications

Taxonomy: Acinetobacter baumannii ATCC 17978

Links to retrieve other entries containing this sequence from NCBI Entrez:

gi|260557587 from Acinetobacter baumannii ATCC 19606

gi|190358287 from Acinetobacter baumannii ATCC 17978

Fixed modifications: Carbamidomethyl (C)

Variable modifications: Oxidation (M)

Cleavage by Trypsin: cuts C-term side of KR unless next residue is P

Number of mass values searched: **37**

Number of mass values matched: **34**

Sequence Coverage: **71%**

Matched peptides shown in **Bold Red**

1 MSSGR**IIQII** **GAVIDVEFER** TSVPK**IYDAL** **QVDGTETTLE** **VQQQLGDGVV**

51 **RTIAMGSTEG** **LKRGLTVTST** **NAPISVPVGT** **ATLGRIMDVL** **GRPIDEAGPV**

101 **ATEERLPIHR** **QAPSYAEQAA** **STDLLETGIK** **VIDLLCPFAK** GGK**VGLFGGA**

151 **GVGK**TVNMME LINNIAK**AHS** **GLSVFAGVGE** **RTREGNDFYH** **EMK**DSNVLDK

201 **VAMVYGQMNE** **PPGNR**LRVAL **TGLTMAEYFR** DEKDENGK**GR** **DVLLFVDNIY**

251 **RYTLAGTEVS** **ALLGRMPSAV** **GYQPTLAEEM** **GVLQER**ITST KSGSITSIQA

301 VYVPADDLTD PSPATTFAHL DATVVLSR**DI** **ASSGIYPAID** **PLDSTSRQLD**

351 **PLVVGQEHYE** **IARAVQNVLQ** RYKELK**DIIA** **ILGMDELAEE** **DKLVVYR**ARK

401 IQR**FFSQPFH** **VAEVFTGAPG** **KLVPLKETIR** GFKGLLAGEY DHIPEQAFYM

451 VGGIDEVIAK AEKL

Start - End	Observed	Mr(expt)	Mr(calc)	ppm	Miss	Sequence
6 - 20	1714.9147	1713.9074	1713.9665	-34	0	R.IIQIIGAVIDVEFER.T
26 - 51	2847.5025	2846.4952	2846.4298	23	0	K.IYDALQVDGTETTLEVQQQLGDGVVR.T
52 - 63	1263.6660	1262.6587	1262.6653	-5	1	R.TIAMGSTEGLKR.G
52 - 63	1279.6591	1278.6518	1278.6602	-7	1	R.TIAMGSTEGLKR.G Oxidation (M)

64 - 85	2112.1003	2111.0930	2111.1587	-31	0	R.GLTVTSTNAPISVPVGTATLGR.I	
86 - 105	2168.0430	2167.0357	2167.0943	-27	0	R.IMDVLGRPIDEAGPVATEER.L	
86 - 105	2184.0423	2183.0350	2183.0892	-25	0	R.IMDVLGRPIDEAGPVATEER.L	Oxidation (M)
106 - 110	635.4604	634.4531	634.3915	97	0	R.LPIHR.Q	
111 - 130	2092.9708	2091.9635	2092.0324	-33	0	R.QAPSYAEQAASTDLLETGIK.V	
131 - 140	1175.6540	1174.6467	1174.6420	4	0	K.VIDLLCPFAK.G	
144 - 154	961.5812	960.5739	960.5393	36	0	K.VGLFGGAGVGK.T	
168 - 181	1386.6885	1385.6812	1385.7052	-17	0	K.AHSGLSVFAGVGER.T	
182 - 193	1526.6274	1525.6201	1525.6620	-27	1	R.TREGNDFYHEMK.D	
182 - 193	1542.6199	1541.6126	1541.6569	-29	1	R.TREGNDFYHEMK.D	Oxidation (M)
184 - 193	1269.5114	1268.5041	1268.5132	-7	0	R.EGNDFYHEMK.D	
201 - 215	1662.7151	1661.7078	1661.7654	-35	0	K.VAMVYGQMNEPPGNR.L	
201 - 215	1678.7100	1677.7027	1677.7603	-34	0	K.VAMVYGQMNEPPGNR.L	Oxidation (M)
201 - 215	1694.7012	1693.6939	1693.7552	-36	0	K.VAMVYGQMNEPPGNR.L	2 Oxidation (M)
218 - 230	1471.7251	1470.7178	1470.7541	-25	0	R.VALTGLTMAEYFR.D	
218 - 230	1487.7197	1486.7124	1486.7490	-25	0	R.VALTGLTMAEYFR.D	Oxidation (M)
239 - 251	1579.8087	1578.8014	1578.8518	-32	1	K.GRDVLLFVDNIYR.Y	
241 - 251	1366.7133	1365.7060	1365.7292	-17	0	R.DVLLFVDNIYR.Y	
252 - 265	1450.7573	1449.7500	1449.7827	-23	0	R.YTLAGTEVSALLGR.M	
266 - 286	2306.0699	2305.0626	2305.1082	-20	0	R.MPSAVGYQPTLAEEMGVLQER.I	
266 - 286	2322.0645	2321.0572	2321.1031	-20	0	R.MPSAVGYQPTLAEEMGVLQER.I	Oxidation (M)
266 - 286	2338.0631	2337.0558	2337.0981	-18	0	R.MPSAVGYQPTLAEEMGVLQER.I	2 Oxidation (M)
329 - 347	1977.9091	1976.9018	1976.9691	-34	0	R.DIASSGIYPAIDPLDSTSR.Q	
348 - 363	1866.9046	1865.8973	1865.9635	-35	0	R.QLDPLVVGQEHYEIAR.A	
364 - 371	927.5754	926.5681	926.5298	41	0	R.AVQNVLQR.Y	
377 - 397	2405.2278	2404.2205	2404.2559	-15	1	K.DIIAILGMDELAEEDKLVVYR.A	
377 - 397	2421.2230	2420.2157	2420.2508	-15	1	K.DIIAILGMDELAEEDKLVVYR.A	Oxidation (M)
393 - 397	649.4636	648.4563	648.3959	93	0	K.LVVYR.A	
404 - 421	1965.9117	1964.9044	1964.9785	-38	0	R.FFSQPFHVAEVFTGAPGK.L	
422 - 430	1068.6985	1067.6912	1067.6703	20	1	K.LVPLKETIR.G	

No match to: 1431.1599, 1849.8769, 2127.1738

25.

Match to: **gi|126642887** Score: **178** Expect: **1.1e-11 Decoy: 68**
putative antioxidant protein [Acinetobacter baumannii ATCC 17978]

Nominal mass (M_r): **18779**; Calculated pI value: **6.32**
NCBI BLAST search of gi|126642887 against nr
Unformatted sequence string for pasting into other applications

Taxonomy: Acinetobacter baumannii ATCC 17978

Fixed modifications: Carbamidomethyl (C)
Variable modifications: Oxidation (M)
Cleavage by Trypsin: cuts C-term side of KR unless next residue is P
Number of mass values searched: **14**
Number of mass values matched: **10**
Sequence Coverage: **73%**

Matched peptides shown in **Bold Red**

```
  1 MGYTAKLKDE FEKRNVKAIA LSVDDVESHK GWINDINETQ NTTVNFPIIA
 51 DKDRKVSELY GFIHPNASET LTVRSLVIID PNKKVRLIIT YPASTGRNFN
101 EVLRVVDSLQ LTDKHKVATP ANWQQGEDVV IVPSLKDEEE IKQRFPKGYT
151 AVKPYLRLTP QPEQD
```

Start - End	Observed	Mr(expt)	Mr(calc)	ppm	Miss	Sequence
18 - 30	1383.6887	1382.6814	1382.7041	-16	0	K.AIALSVDDVESHK.G
31 - 54	2774.4403	2773.4330	2773.3671	24	1	K.GWINDINETQNTTVNFPIIADKDR.K
55 - 74	2261.1590	2260.1517	2260.1852	-15	1	R.KVSELYGFIHPNASETLTVR.S
56 - 74	2133.0464	2132.0391	2132.0902	-24	0	K.VSELYGFIHPNASETLTVR.S
75 - 84	1126.6869	1125.6796	1125.6757	3	1	R.SLVIIDPNKK.V
87 - 97	1191.6666	1190.6593	1190.6659	-6	0	R.LIITYPASTGR.N
87 - 104	2064.0868	2063.0795	2063.1163	-18	1	R.LIITYPASTGRNFNEVLR.V
98 - 104	891.4935	890.4862	890.4610	28	0	R.NFNEVLR.V
117 - 142	2894.5760	2893.5687	2893.4709	34	1	K.VATPANWQQGEDVVIVPSLKDEEEIK.Q
148 - 157	1167.6486	1166.6413	1166.6448	-3	0	K.GYTAVKPYLR.L

No match to: 1242.5758, 1299.7505, 1414.7543, 1546.7702

28.

Match to: **gi|126642864** Score: **262** Expect: **4.3e-20** **Decoy: 79**
outer membrane protein A [Acinetobacter baumannii ATCC 17978]

Nominal mass (M_r): **37342**; Calculated pI value: **5.13**
NCBI BLAST search of gi|126642864 against nr
Unformatted sequence string for pasting into other applications

Taxonomy: Acinetobacter baumannii ATCC 17978

Fixed modifications: Carbamidomethyl (C)
Variable modifications: Oxidation (M)
Cleavage by Trypsin: cuts C-term side of KR unless next residue is P
Number of mass values searched: **20**
Number of mass values matched: **17**
Sequence Coverage: **43%**

Matched peptides shown in **Bold Red**

```
  1 MLVAAPLAAA NAGVTVTPLL LGYTFQDSQH NNGGKDGNLT NGPELQDDLF
 51 VGAALGIELT PWLGFEAEYN QVKGDVDGAS AGAEYKQKQI NGNFYVTSDL
101 ITKNYDSKIK PYVLLGAGHY KYDFDGVNRG TRGTSEEGTL GNAGVGAFWR
151 LNDALSLRTE ARATYNADEE FWNYTALAGL NVVLGGHLKP AAPVVEVAPV
201 EPTPVTPQPQ ELTEDLNMEL RVFFDTNKSN IKDQYKPEIA KVAEKLSEYP
251 NATARIEGHT DNTGPRKLNE RLSLARANSV KSALVNEYNV DASRLSTQGF
301 AWDQPIADNK TKEGRAMNRR VFATITGSRT VVVQPGQEAA APAAAQ
```

Start - End	Observed	Mr(expt)	Mr(calc)	ppm	Miss	Sequence
89 - 103	1712.8559	1711.8486	1711.8781	-17	0	K.QINGNFYVTSDLITK.N
109 - 121	1458.8313	1457.8240	1457.8394	-11	0	K.IKPYVLLGAGHYK.Y
122 - 129	985.4552	984.4479	984.4301	18	0	K.YDFDGVNR.G
133 - 150	1808.8167	1807.8094	1807.8489	-22	0	R.GTSEEGTLGNAGVGAFWR.L
151 - 158	901.5404	900.5331	900.5029	34	0	R.LNDALSLR.T
222 - 228	870.4847	869.4774	869.4283	56	0	R.VFFDTNK.S
229 - 241	1533.8078	1532.8005	1532.8198	-13	1	K.SNIKDQYKPEIAK.V
233 - 241	1091.6190	1090.6117	1090.5658	42	0	K.DQYKPEIAK.V
246 - 255	1121.5657	1120.5584	1120.5513	6	0	K.LSEYPNATAR.I
246 - 266	2299.1718	2298.1645	2298.0989	29	1	K.LSEYPNATARIEGHTDNTGPR.K
256 - 266	1196.5657	1195.5584	1195.5582	0	0	R.IEGHTDNTGPR.K
267 - 271	659.4229	658.4156	658.3762	60	1	R.KLNER.L
282 - 294	1437.6716	1436.6643	1436.6895	-18	0	K.SALVNEYNVDASR.L
295 - 310	1790.8396	1789.8323	1789.8635	-17	0	R.LSTQGFAWDQPIADNK.T
316 - 320	663.3868	662.3795	662.3282	77	1	R.AMNRR.V Oxidation (M)
320 - 329	1107.6350	1106.6277	1106.6196	7	1	R.RVFATITGSR.T
321 - 329	951.5485	950.5412	950.5185	24	0	R.VFATITGSR.T

No match to: 1480.7873, 1824.8269, 2446.2634

29.

Match to: **gi|169796478** Score: **194** Expect: **2.7e-13 Decoy: 79**
LamB/YcsF family protein [Acinetobacter baumannii AYE]

Nominal mass (M_r): **27060**; Calculated pI value: **5.10**
NCBI BLAST search of gi|169796478 against nr
Unformatted sequence string for pasting into other applications

Taxonomy: Acinetobacter baumannii AYE
Links to retrieve other entries containing this sequence from NCBI Entrez:
gi|169149405 from Acinetobacter baumannii AYE
gi|213055569 from Acinetobacter baumannii AB0057
gi|213988226 from Acinetobacter baumannii AB307-0294

Fixed modifications: Carbamidomethyl (C)
Variable modifications: Oxidation (M)
Cleavage by Trypsin: cuts C-term side of KR unless next residue is P
Number of mass values searched: **18**
Number of mass values matched: **14**
Sequence Coverage: **54%**

Matched peptides shown in **Bold Red**

```
  1 MFVDLNSDLG ESFGSWKMGN DDQILPVVTS ANIACGFHAG DPLGILKTVR
 51 KAVELGVTIG AHVSYPDLVG FGRRNMDLSR DELIADVLYQ ISALDGLAKV
101 AGSKVQYVKP HGALYNTIAY DQVQAAAVID AIKMYNPELV LVALAGSNLV
151 EQARAAGLKV VSEAFADRAY NSDGSLVSRR LEGAVLHDSA FVASRVVSML
201 KNGGVESIDG VFTPIQADTI CLHGDTDGAL EMSAAIKAEL VKNNIEIRPF
251 VNKA
```

Start - End	Observed	Mr(expt)	Mr(calc)	ppm	Miss	Sequence
1 - 17	1931.8519	1930.8446	1930.8771	-17	0	-.MFVDLNSDLGESFGSWK.M
1 - 17	1947.8446	1946.8373	1946.8720	-18	0	-.MFVDLNSDLGESFGSWK.M Oxidation (M)
52 - 73	2257.1887	2256.1814	2256.1903	-4	0	K.AVELGVTIGAHVSYPDLVGFGR.R
75 - 99	2779.4893	2778.4820	2778.4109	26	1	R.NMDLSRDELIADVLYQISALDGLAK.V Oxidation (M)
81 - 99	2047.0620	2046.0547	2046.0885	-16	0	R.DELIADVLYQISALDGLAK.V
134 - 154	2287.2103	2286.2030	2286.2042	-0	0	K.MYNPELVLVALAGSNLVEQAR.A
134 - 154	2303.2058	2302.1985	2302.1991	-0	0	K.MYNPELVLVALAGSNLVEQAR.A Oxidation (M)
160 - 168	993.5228	992.5155	992.4927	23	0	K.VVSEAFADR.A
169 - 179	1168.5586	1167.5513	1167.5520	-1	0	R.AYNSDGSLVSR.R
180 - 195	1727.8832	1726.8759	1726.9115	-21	1	R.RLEGAVLHDSAFVASR.V
181 - 195	1571.7879	1570.7806	1570.8103	-19	0	R.LEGAVLHDSAFVASR.V
238 - 253	1883.8415	1882.8342	1883.0628	-121	1	K.AELVKNNIEIRPFVNK.A
243 - 253	1343.7301	1342.7228	1342.7357	-10	0	K.NNIEIRPFVNK.A
243 - 254	1414.7605	1413.7532	1413.7728	-14	1	K.NNIEIRPFVNKA.-

No match to: 1002.5573, 1100.6510, 1296.6799, 1711.8235

30.

Match to: **gi|169794650** Score: **315** Expect: **2.1e-25 Decoy: 74**
malate dehydrogenase [Acinetobacter baumannii AYE]

Nominal mass (M_r): **35358**; Calculated pI value: **5.20**
NCBI BLAST search of gi|169794650 against nr
Unformatted sequence string for pasting into other applications

Taxonomy: Acinetobacter baumannii AYE
Links to retrieve other entries containing this sequence from NCBI Entrez:
gi|260557083 from Acinetobacter baumannii ATCC 19606
gi|152032571 from Acinetobacter baumannii ATCC 17978

Fixed modifications: Carbamidomethyl (C)
Variable modifications: Oxidation (M)
Cleavage by Trypsin: cuts C-term side of KR unless next residue is P
Number of mass values searched: **30**
Number of mass values matched: **26**
Sequence Coverage: **64%**

Matched peptides shown in **Bold Red**

```
  1 MKQPVRVAVT GAAGQIGYSL LFRIASGEML GKDQPVILQL LEVPVEKAQQ
 51 ALKGVMMELD DCAFPLLAGM IGTDDPKVAF KDADYALLVG SRPRGPGMER
101 ADLLKVNGEI FIGQGQALNE VASRDVKVLV VGNPANTNAY IAMKSAPDLP
151 AKNFTAMLRL DHNRALTQVA QKAGVAVADI EKLTVWGNHS PTMYADYRFA
201 TANGESLKDK INDPAWNKDV FLPTVGKRGA AIIEARGLSS AASAANAAID
251 HMRDWALGTN GKWVTMGVPS DGSYGIPEGV MFGFPVTTEN GEYKIVQGLE
301 IDEFSRERIN FTLNELEEER AAIADMVK
```

Start - End	Observed	Mr(expt)	Mr(calc)	ppm	Miss	Sequence
1 - 6	758.4798	757.4725	757.4269	60	1	-.MKQPVR.V
1 - 6	774.4708	773.4635	773.4218	54	1	-.MKQPVR.V Oxidation (M)
7 - 23	1722.9204	1721.9131	1721.9465	-19	0	R.VAVTGAAGQIGYSLLFR.I
33 - 47	1719.9383	1718.9310	1718.9818	-30	0	K.DQPVILQLLEVPVEK.A
78 - 94	1877.9972	1876.9899	1877.0159	-14	1	K.VAFKDADYALLVGSRPR.G
82 - 94	1432.7388	1431.7315	1431.7470	-11	0	K.DADYALLVGSRPR.G
95 - 100	646.3379	645.3306	645.2904	62	0	R.GPGMER.A
95 - 100	662.3327	661.3254	661.2854	61	0	R.GPGMER.A Oxidation (M)
106 - 124	2002.0091	2001.0018	2001.0279	-13	0	K.VNGEIFIGQGQALNEVASR.D
128 - 144	1774.8865	1773.8792	1773.9447	-37	0	K.VLVVGNPANTNAYIAMK.S
153 - 159	852.4747	851.4674	851.4323	41	0	K.NFTAMLR.L
153 - 159	868.4746	867.4673	867.4273	46	0	K.NFTAMLR.L Oxidation (M)
160 - 164	654.3666	653.3593	653.3245	53	0	R.LDHNR.A
160 - 172	1493.7296	1492.7223	1492.8110	-59	1	R.LDHNRALTQVAQK.A

**183 - 198**	**1910.8491**	**1909.8418**	**1909.8781**	**-19**	**0**	**K.LTVWGNHSPTMYADYR.F**	
**183 - 198**	**1926.8506**	**1925.8433**	**1925.8730**	**-15**	**0**	**K.LTVWGNHSPTMYADYR.F**	Oxidation (M)
**199 - 210**	**1280.6576**	**1279.6503**	**1279.6408**	**7**	**1**	**R.FATANGESLKDK.I**	
**219 - 227**	**975.5865**	**974.5792**	**974.5437**	**36**	**0**	**K.DVFLPTVGK.R**	
**219 - 228**	**1131.6699**	**1130.6626**	**1130.6448**	**16**	**1**	**K.DVFLPTVGKR.G**	
**228 - 236**	**956.5964**	**955.5891**	**955.5563**	**34**	**1**	**K.RGAAIIEAR.G**	
**229 - 236**	**800.5021**	**799.4948**	**799.4552**	**50**	**0**	**R.GAAIIEAR.G**	
**237 - 253**	**1642.7779**	**1641.7706**	**1641.7893**	**-11**	**0**	**R.GLSSAASAANAAIDHMR.D**	
**237 - 253**	**1658.7903**	**1657.7830**	**1657.7842**	**-1**	**0**	**R.GLSSAASAANAAIDHMR.D**	Oxidation (M)
**237 - 262**	**2601.3426**	**2600.3353**	**2600.2401**	**37**	**1**	**R.GLSSAASAANAAIDHMRDWALGTNGK.W**	Oxidation (M)
**295 - 306**	**1405.7123**	**1404.7050**	**1404.7249**	**-14**	**0**	**K.IVQGLEIDEFSR.E**	
**309 - 320**	**1506.7211**	**1505.7138**	**1505.7361**	**-15**	**0**	**R.INFTLNELEEER.A**	

No match to: 1395.4930, 1417.5800, 1602.1736, 1984.9741

32.

Match to: **gi|162286753** Score: **335** Expect: **2.1e-27** **Decoy: 69**
elongation factor Tu [Acinetobacter baumannii ATCC 17978]

Nominal mass (M_r): **43150**; Calculated pI value: **5.21**
NCBI BLAST search of gi|162286753 against nr
Unformatted sequence string for pasting into other applications

Taxonomy: Acinetobacter baumannii ATCC 17978
Links to retrieve other entries containing this sequence from NCBI Entrez:
gi|169634056 from Acinetobacter baumannii SDF
gi|169797460 from Acinetobacter baumannii AYE

Fixed modifications: Carbamidomethyl (C)
Variable modifications: Oxidation (M)
Cleavage by Trypsin: cuts C-term side of KR unless next residue is P
Number of mass values searched: **30**
Number of mass values matched: **27**
Sequence Coverage: **64%**

Matched peptides shown in **Bold Red**

```
  1 MAKAKFERNK PHVNVGTIGH VDHGKTTLTA AIATICAKTY GGEAKDYSQI
 51 DSAPEEKARG ITINTSHVEY DSPTRHYAHV DCPGHADYVK NMITGAAQMD
101 GAILVCAATD GPMPQTREHI LLSRQVGVPY IIVFLNKCDL VDDEELLELV
151 EMEVRELLST YDFPGDDTPV IRGSALAALN GEAGPYGEES VLALVAALDS
201 YIPEPERAID KAFLMPIEDV FSISGRGTVV TGRVEAGIIK VGEEVEIVGI
251 KDTVKTTVTG VEMFRKLLDE GRAGENCGIL LRGTKREEVQ RGQVLAKPGT
301 IKPHTKFDAE VYVLSKEEGG RHTPFLNGYR PQFYFRTTDV TGAIQLKEGV
351 EMVMPGDNVE MSVELIHPIA MDPGLRFAIR EGGRTVGAGV VAKVTA
```

Start - End	Observed	Mr(expt)	Mr(calc)	ppm	Miss	Sequence
4 - 8	650.3752	649.3679	649.3547	20	1	K.AKFER.N
9 - 25	1808.9346	1807.9273	1807.9442	-9	0	R.NKPHVNVGTIGHVDHGK.T
26 - 38	1334.7143	1333.7070	1333.7275	-15	0	K.TTLTAAIATICAK.T
60 - 75	1789.8528	1788.8455	1788.8642	-10	0	R.GITINTSHVEYDSPTR.H
76 - 90	1768.7700	1767.7627	1767.7787	-9	0	R.HYAHVDCPGHADYVK.N
91 - 117	2822.4823	2821.4750	2821.2867	67	0	K.NMITGAAQMDGAILVCAATDGPMPQTR.E 2 Oxidation (M)
91 - 117	2838.4933	2837.4860	2837.2816	72	0	K.NMITGAAQMDGAILVCAATDGPMPQTR.E 3 Oxidation (M)
118 - 124	867.5204	866.5131	866.4974	18	0	R.EHILLSR.Q
125 - 137	1489.8802	1488.8729	1488.8704	2	0	R.QVGVPYIIVFLNK.C
138 - 155	2206.0505	2205.0432	2205.0181	11	0	K.CDLVDDEELLELVEMEVR.E
138 - 155	2222.0536	2221.0463	2221.0130	15	0	K.CDLVDDEELLELVEMEVR.E Oxidation (M)
156 - 172	1937.9444	1936.9371	1936.9418	-2	0	R.ELLSTYDFPGDDTPVIR.G
212 - 226	1681.8477	1680.8404	1680.8545	-8	0	K.AFLMPIEDVFSISGR.G
212 - 226	1697.8434	1696.8361	1696.8494	-8	0	K.AFLMPIEDVFSISGR.G Oxidation (M)
227 - 233	689.4100	688.4027	688.3868	23	0	R.GTVVTGR.V
241 - 255	1614.8743	1613.8670	1613.8876	-13	1	K.VGEEVEIVGIKDTVK.T
256 - 265	1140.5686	1139.5613	1139.5645	-3	0	K.TTVTGVEMFR.K
256 - 265	1156.5643	1155.5570	1155.5594	-2	0	K.TTVTGVEMFR.K Oxidation (M)
256 - 266	1268.6545	1267.6472	1267.6595	-10	1	K.TTVTGVEMFRK.L
256 - 266	1284.6482	1283.6409	1283.6544	-10	1	K.TTVTGVEMFRK.L Oxidation (M)
266 - 272	830.4857	829.4784	829.4657	15	1	R.KLLDEGR.A
267 - 272	702.3951	701.3878	701.3708	24	0	K.LLDEGR.A
273 - 282	1102.5694	1101.5621	1101.5601	2	0	R.AGENCGILLR.G
287 - 291	660.3425	659.3352	659.3238	17	0	R.EEVQR.G
292 - 306	1574.9112	1573.9039	1573.9304	-17	0	R.GQVLAKPGTIKPHTK.F
307 - 316	1170.6031	1169.5958	1169.5968	-1	0	K.FDAEVYVLSK.E
322 - 336	1942.9809	1941.9736	1941.9638	5	0	R.HTPFLNGYRPQFYFR.T

No match to: 1046.6202, 1475.7309, 1641.1866

33.

Match to: **gi|193077956** Score: **173** Expect: **4e-09** **Decoy: 62**
NAD-dependent aldehyde dehydrogenase [Acinetobacter baumannii ATCC 17978]

Nominal mass (M_r): **51319**; Calculated pI value: **5.29**
NCBI BLAST search of gi|193077956 against nr
Unformatted sequence string for pasting into other applications

Taxonomy: Acinetobacter baumannii ATCC 17978

Fixed modifications: Carbamidomethyl (C)
Variable modifications: Oxidation (M)
Cleavage by Trypsin: cuts C-term side of KR unless next residue is P
Number of mass values searched: **10**
Number of mass values matched: **10**
Sequence Coverage: **26%**

Matched peptides shown in **Bold Red**

```
  1 MQQANMLNEF KLIIGGQLCS GEQGELEIIN PATGLTAAHC AKASVAQVNQ
 51 AVSAAKQASK AWQLVSHDER KSILNKIADG IEKHAEMLAE LVVLEQGKPL
101 ALAQMEVQGA IGWTRYAASM DLPVEVIEDS ETKRIERHRQ PLGVVASITP
151 WNWPLMIAVW HIMPALRAGN VVISKPSEYT PLSTLRLCEI IQQEVPAGVI
201 SIVVGAGEIG EALSSHPDVQ KVVFTGSTRT GQHIMAGAAQ QLKHLTLELG
251 GNDAGIVLPD ANIDEIAAKI FNMAFLNAGQ TCAALKRLYV HESQYEALSQ
301 KLADIANAQV VGDGMASSTT FGPVQNQMQY NKVKALIAEA IAQGAKALSG
351 QQQLPEQGYF IAPTILTEVS DSCRVVQEEQ FGPVLPVLKY TDINDAIARA
401 NDSEFGLGGS IWSSDLKAAQ TYATQLQCGT VWINTHAEVL PHAPFGGWKM
451 SGLGAEFGLE GLLENTIGQT VHISKI
```

Start - End	Observed	Mr(expt)	Mr(calc)	ppm	Miss	Sequence	
61 - 70	1240.6113	1239.6040	1239.5996	4	0	K.AWQLVSHDER.K	
61 - 71	1368.6873	1367.6800	1367.6946	-11	1	K.AWQLVSHDERK.S	
116 - 134	2168.9359	2167.9286	2168.0307	-47	1	R.YAASMDLPVEVIEDSETKR.I	Oxidation (M)
168 - 186	2032.0631	2031.0558	2031.1000	-22	0	R.AGNVVISKPSEYTPLSTLR.L	
222 - 229	866.4648	865.4575	865.4658	-10	0	K.VVFTGSTR.T	
287 - 301	1850.9041	1849.8968	1849.9322	-19	1	K.RLYVHESQYEALSQK.L	
288 - 301	1694.8180	1693.8107	1693.8311	-12	0	R.LYVHESQYEALSQK.L	
347 - 374	3108.5952	3107.5879	3107.5234	21	0	K.ALSGQQQLPEQGYFIAPTILTEVSDSCR.V	
375 - 389	1681.8993	1680.8920	1680.9451	-32	0	R.VVQEEQFGPVLPVLK.Y	
390 - 399	1151.5867	1150.5794	1150.5618	15	0	K.YTDINDAIAR.A	

34.

Match to: **gi|193076354** Score: **317** Expect: **1.4e-25 Decoy: 88**
aconitate hydratase 1 [Acinetobacter baumannii ATCC 17978]

Nominal mass (M_r): **100488**; Calculated pI value: **5.25**
NCBI BLAST search of gi|193076354 against nr
Unformatted sequence string for pasting into other applications

Taxonomy: Acinetobacter baumannii ATCC 17978

Fixed modifications: Carbamidomethyl (C)
Variable modifications: Oxidation (M)
Cleavage by Trypsin: cuts C-term side of KR unless next residue is P
Number of mass values searched: **30**
Number of mass values matched: **28**
Sequence Coverage: **32%**

Matched peptides shown in **Bold Red**

```
  1 MAKYSNINSF NALQTLTVGS SNYQIFSLTQ AEKKLGDIAK LPKSLKVLLE
 51 NLLRFEDQHS VKTEHIYALA EWLKTRTSDQ EIQYRPARVL MQDFTGVPAV
101 VDLAAMRAAM AEAGGDPEKI NPLSPVDLVI DHSVMVDHFA DDQAFEENVQ
151 IEMQRNGERY QFLRWGQSAF NNFSVVPPGT GICHQVNLEY LAQAVWLGED
201 NGQTFAFPDT LVGTDSHTTM INGLGVLGWG VGGIEAEAAM LGQPISMLIP
251 EVIGFKLTGK LQEGITATDL VLTITQMLRQ KGVVGKFVEF YGDGLADLPL
301 ADRATIANMA PEYGATCGFF PIDEVTLGYL KLTGRQSDRI ALVEAYSKAQ
351 GLWRNPGDEP VFTDTLSLDM STVQASLAGP KRPQDRVLLS EVPKTFNALM
401 ELTLKPAKEA KERLENEGGG GTAVEATKAN IQHESPSCVI EGQEYPLNHG
451 DVVISAITSC TNTSNPSVML AAGLLAKKAI EKGLQRKPWV KSSLAPGSKV
501 VTDYLLAAGL TPYLDELGYN LVGYGCTTCI GNSGPLPEPI EDAIQCHDLN
551 VASVLSGNRN FEGRVHPLVK TNWLASPPLV VAYGLVGNIR TDLTTQPIGQ
601 GKDGQPVYLK DIWPSQAEID AVLQKVNTDM FHKEYAAVFD GDESWQAIQI
651 PISKTYEWAD DSTYIRHPPF FEGIGEPPKP IKNIEQARIL AVLGDSVTTD
701 HISPAGNIKK DSPAGRYLQE QGVEPKDFNS YGSRRGNHEV MMRGTFANIR
751 IKNEMLGGEE GGNTIHVPSG EKLAIYDAAM RYQQEHTPLV IIAGKEYGTG
801 SSRDWAAKGT NLLGVKAVIA ESFERIHRSN LVGMGVLPLQ FVDGQTRQSL
851 NLTGHEVISI RGLSDGIQPH EILEVDVKGP NGVASHFNVL CRIDTLNEVE
901 YFKAGGILHY VLRNLIAS
```

Start - End	Observed	Mr(expt)	Mr(calc)	ppm	Miss	Sequence	
35 - 40	616.4137	615.4064	615.3592	77	0	K.LGDIAK.L	
47 - 54	969.6404	968.6331	968.6018	32	0	K.VLLENLLR.F	
55 - 62	989.5230	988.5157	988.4614	55	0	R.FEDQHSVK.T	
77 - 88	1463.6929	1462.6856	1462.7164	-21	0	R.TSDQEIQYRPAR.V	
89 - 107	2064.9938	2063.9865	2064.0384	-25	0	R.VLMQDFTGVPAVVDLAAMR.A	2 Oxidation (M)
160 - 164	726.4486	725.4413	725.3860	76	0	R.YQFLR.W	
261 - 279	2132.1077	2131.1004	2131.1559	-26	0	K.LQEGITATDLVLTITQMLR.Q	Oxidation (M)

287 - 303	1897.8724	1896.8651	1896.9258	-32	0	K.FVEFYGDGLADLPLADR.A	
349 - 354	730.4517	729.4444	729.3922	72	0	K.AQGLWR.N	
382 - 386	671.4169	670.4096	670.3511	87	0	K.RPQDR.V	
560 - 564	622.3555	621.3482	621.2871	98	0	R.NFEGR.V	
655 - 666	1519.6324	1518.6251	1518.6627	-25	0	K.TYEWADDSTYIR.H	
667 - 688	2501.2420	2500.2347	2500.3226	-35	1	R.HPPFFEGIGEPPKPIKNIEQAR.I	
689 - 709	2121.0994	2120.0921	2120.1477	-26	0	R.ILAVLGDSVTTDHISPAGNIK.K	
727 - 734	945.4442	944.4369	944.3988	40	0	K.DFNSYGSR.R	
735 - 743	1145.5432	1144.5359	1144.5230	11	1	R.RGNHEVMMR.G	Oxidation (M)
735 - 743	1161.5341	1160.5268	1160.5179	8	1	R.RGNHEVMMR.G	2 Oxidation (M)
736 - 743	973.5651	972.5578	972.4269	135	0	R.GNHEVMMR.G	
744 - 750	778.4723	777.4650	777.4133	66	0	R.GTFANIR.I	
773 - 781	1039.5538	1038.5465	1038.5168	29	0	K.LAIYDAAMR.Y	Oxidation (M)
782 - 795	1596.8284	1595.8211	1595.8671	-29	0	R.YQQEHTPLVIIAGK.E	
817 - 825	1021.5581	1020.5508	1020.5240	26	0	K.AVIAESFER.I	
829 - 847	2031.0111	2030.0038	2030.0619	-29	0	R.SNLVGMGVLPLQFVDGQTR.Q	
829 - 847	2047.0073	2046.0000	2046.0568	-28	0	R.SNLVGMGVLPLQFVDGQTR.Q	Oxidation (M)
848 - 861	1566.8164	1565.8091	1565.8525	-28	0	R.QSLNLTGHEVISIR.G	
862 - 878	1848.9103	1847.9030	1847.9629	-32	0	R.GLSDGIQPHEILEVDVK.G	
879 - 892	1527.7059	1526.6986	1526.7412	-28	0	K.GPNGVASHFNVLCR.I	
904 - 913	1098.6531	1097.6458	1097.6346	10	0	K.AGGILHYVLR.N	

No match to: 1247.6923, 1475.7176

35.

Match to: **gi|169633288** Score: **232** Expect: **4.3e-17 Decoy: 75**
peptidyl-prolyl cis-trans isomerase precursor (PPIase) (rotamase) [Acinetobacter baumannii SDF]

Nominal mass (M_r): **18533**; Calculated pI value: **5.40**
NCBI BLAST search of gi|169633288 against nr
Unformatted sequence string for pasting into other applications

Taxonomy: Acinetobacter baumannii SDF
Links to retrieve other entries containing this sequence from NCBI Entrez:
gi|193077690 from Acinetobacter baumannii ATCC 17978
gi|213057651 from Acinetobacter baumannii AB0057
gi|213988989 from Acinetobacter baumannii AB307-0294

Fixed modifications: Carbamidomethyl (C)
Variable modifications: Oxidation (M)
Cleavage by Trypsin: cuts C-term side of KR unless next residue is P
Number of mass values searched: **18**
Number of mass values matched: **14**
Sequence Coverage: **90%**

Matched peptides shown in **Bold Red**

```
  1 MSFPQVELNT NKGRIVLELN TEKAPKTAAN FLEYVRDGFY DGVIFHRVID
 51 GFMIQGGGFD ENFKEKATRD AIENEADNGL SNDVGTIAMA RTQAPHSASA
101 QFFINVKNNS FLNHTSKTAQ GWGYAVFGKV VEGMDVVEAI KGVRTGNRGY
151 HADVPLENVV IESAKIISE
```

Start - End	Observed	Mr(expt)	Mr(calc)	ppm	Miss	Sequence
2 - 12	1276.6399	1275.6326	1275.6459	-10	0	M.SFPQVELNTNK.G
15 - 23	1058.6193	1057.6120	1057.6019	10	0	R.IVLELNTEK.A
27 - 36	1183.6022	1182.5949	1182.6033	-7	0	K.TAANFLEYVR.D
37 - 47	1325.6020	1324.5947	1324.6201	-19	0	R.DGFYDGVIFHR.V
48 - 64	1889.8063	1888.7990	1888.8666	-36	0	R.VIDGFMIQGGGFDENFK.E Oxidation (M)
48 - 66	2146.9577	2145.9504	2146.0041	-25	1	R.VIDGFMIQGGGFDENFKEK.A Oxidation (M)
70 - 91	2276.0216	2275.0143	2275.0386	-11	0	R.DAIENEADNGLSNDVGTIAMAR.T
70 - 91	2292.0168	2291.0095	2291.0335	-10	0	R.DAIENEADNGLSNDVGTIAMAR.T Oxidation (M)
92 - 107	1745.8381	1744.8308	1744.8897	-34	0	R.TQAPHSASAQFFINVK.N
108 - 117	1161.5620	1160.5547	1160.5574	-2	0	K.NNSFLNHTSK.T
118 - 129	1284.6156	1283.6083	1283.6299	-17	0	K.TAQGWGYAVFGK.V
130 - 141	1304.6523	1303.6450	1303.6694	-19	0	K.VVEGMDVVEAIK.G Oxidation (M)
149 - 165	1840.8812	1839.8739	1839.9367	-34	0	R.GYHADVPLENVVIESAK.I
149 - 169	2283.1309	2282.1236	2282.1794	-24	1	R.GYHADVPLENVVIESAKIISE.-

No match to: 1196.6001, 1205.5767, 1270.6557, 1297.7309

36.

Match to: **gi|169634426** Score: **146** Expect: **1.7e-08 Decoy: 76**
ATP-dependent Clp protease proteolytic subunit [Acinetobacter baumannii SDF]

Nominal mass (M_r): **22639**; Calculated pI value: **5.26**
NCBI BLAST search of gi|169634426 against nr
Unformatted sequence string for pasting into other applications

Taxonomy: Acinetobacter baumannii SDF
Links to retrieve other entries containing this sequence from NCBI Entrez:
gi|260556149 from Acinetobacter baumannii ATCC 19606
gi|193076289 from Acinetobacter baumannii ATCC 17978

Fixed modifications: Carbamidomethyl (C)
Variable modifications: Oxidation (M)
Cleavage by Trypsin: cuts C-term side of KR unless next residue is P
Number of mass values searched: **16**
Number of mass values matched: **12**
Sequence Coverage: **41%**

Matched peptides shown in **Bold Red**

```
  1 MYVPTIENAL VPVVVEQSSR GERSFDIYSR LLRERVIFLT GEVEDNMANL
 51 IVAQMLFLEA ENPDKDIHLY INSPGGSVTA GMAIYDTMQF IKPDVVTYCM
101 GQAASMGAFL LNAGAKGKRY CLENARVMIH QPLGGFRGQA SDIEIHAREI
151 LFIKERLNRL MAEHSGQDYD TIARDTDRDN FMTAQAAKEY GLVDQVLSKR
201 P
```

Start - End	Observed	Mr(expt)	Mr(calc)	ppm	Miss	Sequence
24 - 30	887.4632	886.4559	886.4185	42	0	R.SFDIYSR.L
119 - 126	1081.5380	1080.5307	1080.5134	16	1	K.RYCLENAR.V
120 - 126	925.4548	924.4475	924.4123	38	0	R.YCLENAR.V
127 - 137	1254.6757	1253.6684	1253.6703	-1	0	R.VMIHQPLGGFR.G
127 - 137	1270.6745	1269.6672	1269.6652	2	0	R.VMIHQPLGGFR.G Oxidation (M)
138 - 148	1196.6076	1195.6003	1195.5945	5	0	R.GQASDIEIHAR.E
149 - 154	762.5283	761.5210	761.4687	69	0	R.EILFIK.E
160 - 174	1706.7420	1705.7347	1705.7730	-22	0	R.LMAEHSGQDYDTIAR.D
160 - 174	1722.7406	1721.7333	1721.7679	-20	0	R.LMAEHSGQDYDTIAR.D Oxidation (M)
175 - 188	1583.6855	1582.6782	1582.7046	-17	1	R.DTDRDNFMTAQAAK.E
175 - 188	1599.6799	1598.6726	1598.6995	-17	1	R.DTDRDNFMTAQAAK.E Oxidation (M)
189 - 199	1250.6654	1249.6581	1249.6554	2	0	K.EYGLVDQVLSK.R

No match to: 1214.2403, 1286.6669, 1297.7442, 1666.0640

38.

Match to: **gi|169634408** Score: **138** Expect: **1.1e-07** **Decoy: 76**
nucleoside diphosphate kinase (NDK) (NDP kinase) (nucleoside-2-P kinase) [Acinetobacter baumannii SDF]

Nominal mass (M_r): **15510**; Calculated pI value: **5.52**
NCBI BLAST search of gi|169634408 against nr
Unformatted sequence string for pasting into other applications

Taxonomy: Acinetobacter baumannii SDF
Links to retrieve other entries containing this sequence from NCBI Entrez:
gi|193076306 from Acinetobacter baumannii ATCC 17978
gi|260410184 from Acinetobacter baumannii ATCC 19606
gi|292827524 from Acinetobacter sp. SH024

Fixed modifications: Carbamidomethyl (C)
Variable modifications: Oxidation (M)
Cleavage by Trypsin: cuts C-term side of KR unless next residue is P
Number of mass values searched: **9**
Number of mass values matched: **7**
Sequence Coverage: **62%**

Matched peptides shown in **Bold Red**

1 MAIER**TLSIV KPDAVSKNHI GEIFAR**FEKA GLKIVATKMK **HLSQADAEGF**
51 **YAEHK**ERGFF GDLVAFMTSG PVVVSVLEGE NAVLAHR**EIL GATNPKEAAP**
101 **GTIRADFAVS IDENAAHGSD SVASAEREIA YFFADNEICP R**TR

Start - End	Observed	Mr(expt)	Mr(calc)	ppm	Miss	Sequence
**6 - 17**	**1257.7118**	**1256.7045**	**1256.7340**	**-23**	**0**	**R.TLSIVKPDAVSK.N**
**18 - 26**	**1056.5789**	**1055.5716**	**1055.5512**	**19**	**0**	**K.NHIGEIFAR.F**
**41 - 55**	**1702.7346**	**1701.7273**	**1701.7747**	**-28**	**0**	**K.HLSQADAEGFYAEHK.E**
**88 - 96**	**942.5746**	**941.5673**	**941.5182**	**52**	**0**	**R.EILGATNPK.E**
**97 - 104**	**814.4878**	**813.4805**	**813.4344**	**57**	**0**	**K.EAAPGTIR.A**
**105 - 127**	**2319.0276**	**2318.0203**	**2318.0411**	**-9**	**0**	**R.ADFAVSIDENAAHGSDSVASAER.E**
**128 - 141**	**1744.7510**	**1743.7437**	**1743.7926**	**-28**	**0**	**R.EIAYFFADNEICPR.T**

No match to: 1012.5303, 1090.5447

40.

Match to: **gi|169634382** Score: **259** Expect: **8.5e-20**
ketol-acid reductoisomerase [Acinetobacter baumannii SDF]

Nominal mass (M_r): **37001**; Calculated pI value: **5.39**
NCBI BLAST search of gi|169634382 against nr
Unformatted sequence string for pasting into other applications

Taxonomy: Acinetobacter baumannii SDF
Links to retrieve other entries containing this sequence from NCBI Entrez:
gi|226709961 from Acinetobacter baumannii ATCC 17978
gi|260410158 from Acinetobacter baumannii ATCC 19606

Fixed modifications: Carbamidomethyl (C)
Variable modifications: Oxidation (M)
Cleavage by Trypsin: cuts C-term side of KR unless next residue is P
Number of mass values searched: **22**
Number of mass values matched: **18**
Sequence Coverage: **50%**

Matched peptides shown in **Bold Red**

```
  1 MQIFYDKDCD LSIIQSKKVA IIGYGSQGHA HALNLKDSGV DVTVGLRAGS
 51 ASWKKAENAG LKVAEVPAAV KQADLVMILT PDEFQSQLYR DVIEPNIKEG
101 ATLAFAHGFS VLYNQVVPRK DLDVIMVAPK APGHTVRSEF QRGSGVPDLI
151 AIHQDASGNA RNVALSYASG VGGGRTGIIE TSFREETETD LFGEQAVLCG
201 GAVELVKMGF ETLVEAGYAP EMAYFECLHE LKLIVDLMFE GGIADMNYSV
251 SNNAEYGEYV TGPEVINEQS REAMRNALKR IQSGEYAKMF IQEGALNYPS
301 MTARRRQNAA HGIEQTGAKL RAMMPWIQAN KIVDKEKN
```

Start - End	Observed	Mr(expt)	Mr(calc)	ppm	Miss	Sequence
18 - 36	1977.0203	1976.0130	1976.0956	-42	1	K.KVAIIGYGSQGHAHALNLK.D
37 - 47	1117.6022	1116.5949	1116.5775	16	0	K.DSGVDVTVGLR.A
72 - 90	2267.0735	2266.0662	2266.1304	-28	0	K.QADLVMILTPDEFQSQLYR.D
72 - 90	2283.1050	2282.0977	2282.1253	-12	0	K.QADLVMILTPDEFQSQLYR.D Oxidation (M)
99 - 119	2276.1423	2275.1350	2275.1750	-18	0	K.EGATLAFAHGFSVLYNQVVPR.K
131 - 137	737.4363	736.4290	736.3980	42	0	K.APGHTVR.S
138 - 142	666.3563	665.3490	665.3133	54	0	R.SEFQR.G
143 - 161	1877.8987	1876.8914	1876.9391	-25	0	R.GSGVPDLIAIHQDASGNAR.N
162 - 175	1307.6629	1306.6556	1306.6630	-6	0	R.NVALSYASGVGGGR.T
176 - 184	1023.5713	1022.5640	1022.5397	24	0	R.TGIIETSFR.E
272 - 279	948.4403	947.4330	947.4858	-56	1	R.EAMRNALK.R Oxidation (M)
276 - 280	601.4011	600.3938	600.3707	38	1	R.NALKR.I
280 - 288	1051.5689	1050.5616	1050.5458	15	1	K.RIQSGEYAK.M
281 - 288	895.4867	894.4794	894.4447	39	0	R.IQSGEYAK.M
281 - 304	2705.2000	2704.1927	2704.2989	-39	1	R.IQSGEYAKMFIQEGALNYPSMTAR.R
289 - 304	1860.8022	1859.7949	1859.8546	-32	0	K.MFIQEGALNYPSMTAR.R 2 Oxidation (M)

**306 - 319**	**1480.7246**	**1479.7173**	**1479.7542**	**-25**	**1**	**R.RQNAAHGIEQTGAK.L**
**307 - 319**	**1324.6614**	**1323.6541**	**1323.6531**	**1**	**0**	**R.QNAAHGIEQTGAK.L**

No match to: 1022.5777, 1180.7087, 1436.7167, 1473.7306

41.
Match to: **gi|162286736** Score: **275** Expect: **2.1e-21**
fructose-1,6-bisphosphate aldolase [Acinetobacter baumannii ATCC 17978]

Nominal mass (M_r): **37559**; Calculated pI value: **5.43**
NCBI BLAST search of gi|162286736 against nr
Unformatted sequence string for pasting into other applications

Taxonomy: Acinetobacter baumannii ATCC 17978
Links to retrieve other entries containing this sequence from NCBI Entrez:
gi|193077181 from Acinetobacter baumannii ATCC 17978
gi|260411751 from Acinetobacter baumannii ATCC 19606

Fixed modifications: Carbamidomethyl (C)
Variable modifications: Oxidation (M)
Cleavage by Trypsin: cuts C-term side of KR unless next residue is P
Number of mass values searched: **26**
Number of mass values matched: **22**
Sequence Coverage: **55%**

Matched peptides shown in **Bold Red**

```
  1 MALISMRQLL DHAAEHNYGV PAFNVNNLEQ MRAIMLAADA TNSPVIVQAS
 51 AGARKYAGAP FLRHLILAAI EEWPHIPVVM HQDHGTSPDV CQRSIQLGFS
101 SVMMDGSLGA DGKTPTTYDY NVDVTRQVVA MAHACGVSVE GEIGCLGSLE
151 TGMAGEEDGV GAEGVLDHSQ LLTSVEEAKQ FVADTNVDAL AIAVGTSHGA
201 YKFTRPPTGD ILAIDRIKEI HAALPNTHLV MHGSSSVPQE WLKVINEFGG
251 NIGETYGVPV EQLVEAIKHG VRKINIDTDL RLASTGAIRR FMAENPAEFD
301 PRKYFAKTVD SMKQICIDRY EAFGTAGNAD KIRPISLEKM VDRYK
```

Start - End	Observed	Mr(expt)	Mr(calc)	ppm	Miss	Sequence
**2 - 7**	**690.4437**	**689.4364**	**689.3894**	**68**	**0**	**M.ALISMR.Q**
**2 - 7**	**706.4421**	**705.4348**	**705.3843**	**72**	**0**	**M.ALISMR.Q** Oxidation (M)
**8 - 32**	**2880.4578**	**2879.4505**	**2879.3773**	**25**	**0**	**R.QLLDHAAEHNYGVPAFNVNNLEQMR.A**
**8 - 32**	**2896.4821**	**2895.4748**	**2895.3722**	**35**	**0**	**R.QLLDHAAEHNYGVPAFNVNNLEQMR.A** Oxidation (M)
**33 - 54**	**2127.0649**	**2126.0576**	**2126.1154**	**-27**	**0**	**R.AIMLAADATNSPVIVQASAGAR.K**
**33 - 54**	**2143.0627**	**2142.0554**	**2142.1103**	**-26**	**0**	**R.AIMLAADATNSPVIVQASAGAR.K** Oxidation (M)
**55 - 63**	**1022.5903**	**1021.5830**	**1021.5709**	**12**	**1**	**R.KYAGAPFLR.H**

56 - 63	894.5081	893.5008	893.4759	28	0	K.YAGAPFLR.H
114 - 126	1544.6787	1543.6714	1543.7155	-29	0	K.TPTTYDYNVDVTR.Q
180 - 202	2348.1469	2347.1396	2347.1808	-18	0	K.QFVADTNVDALAIAVGTSHGAYK.F
203 - 216	1571.8069	1570.7996	1570.8467	-30	0	K.FTRPPTGDILAIDR.I
244 - 268	2675.4136	2674.4063	2674.3854	8	0	K.VINEFGGNIGETYGVPVEQLVEAIK.H
273 - 281	1087.6191	1086.6118	1086.6033	8	1	R.KINIDTDLR.L
274 - 281	959.5392	958.5319	958.5083	25	0	K.INIDTDLR.L
282 - 289	788.4992	787.4919	787.4552	47	0	R.LASTGAIR.R
290 - 302	1595.6778	1594.6705	1594.7198	-31	1	R.RFMAENPAEFDPR.K Oxidation (M)
291 - 302	1423.5940	1422.5867	1422.6238	-26	0	R.FMAENPAEFDPR.K
291 - 302	1439.5908	1438.5835	1438.6187	-24	0	R.FMAENPAEFDPR.K Oxidation (M)
314 - 319	804.4434	803.4361	803.3960	50	0	K.QICIDR.Y
314 - 331	2028.8761	2027.8688	2027.9371	-34	1	K.QICIDRYEAFGTAGNADK.I
320 - 331	1243.5501	1242.5428	1242.5517	-7	0	R.YEAFGTAGNADK.I
332 - 339	955.6165	954.6092	954.5862	24	0	K.IRPISLEK.M

No match to: 1383.0479, 1555.7100, 1556.5848, 2086.2094

43.

Match to: **gi|193076292** Score: **289** Expect: **8.5e-23 Decoy: 77**
fumarate hydratase [Acinetobacter baumannii ATCC 17978]

Nominal mass (M_r): **55233**; Calculated pI value: **5.44**
NCBI BLAST search of gi|193076292 against nr
Unformatted sequence string for pasting into other applications

Taxonomy: Acinetobacter baumannii ATCC 17978

Fixed modifications: Carbamidomethyl (C)
Variable modifications: Oxidation (M)
Cleavage by Trypsin: cuts C-term side of KR unless next residue is P
Number of mass values searched: **27**
Number of mass values matched: **24**
Sequence Coverage: **52%**

Matched peptides shown in **Bold Red**

1 MTTIIKQDDL ITSIK**DALQF ISYYHPQDFI QAMSRAYDRE ENK**AAK**DAIA**
51 **QILINSR**MCA EGHRPICQDT GIVNVFLEVG LDVK**FDLTMS LDDAVNEGVR**
101 **QGYLENSNVL RASVLADPAF GRK**NTK**DNTP AVIHYK**LVPG NKVDITVAAK
151 GGGSENKSKL AMLNPSDSIV DWVLKTVPTM GAGWCPPGML GIGIGGTAEK
201 AMMLAK**EALM EEINMDELLR** RGPENKIEEL RIEIFEK**VNA LGIGAQGLGG**
251 **LTTVLDIK**IK DYPCHAAGKP VGMIPNCAAT R**HAHFQLDGS GVAHIQAPK**L
301 EDYPSVTWDA SQSKRVNLDT ITQEEMDSWK PGDTLLLSGT MYTGRDAAHK

```
351 RMVEMIDNGE ELPIDLKGKF IYYVGPVDPV RDEVVGPAGP TTATRMDKFT
401 RKVLEHTGLF GMIGKADRGP TAIEAIKDNK ATYLMAVGGA AYLVSKAVRE
451 AEVVAFADLG MEAIYKFVVE DMPVSVAVDV NGTSIHAVAP KIWQAKIGKI
501 PVIDAAAG
```

Start - End	Observed	Mr(expt)	Mr(calc)	ppm	Miss	Sequence
16 - 35	2446.2015	2445.1942	2445.1423	21	0	K.DALQFISYYHPQDFIQAMSR.A Oxidation (M)
36 - 43	1024.4964	1023.4891	1023.4621	26	1	R.AYDREENK.A
47 - 57	1213.6952	1212.6879	1212.6826	4	0	K.DAIAQILINSR.M
85 - 100	1781.7934	1780.7861	1780.8302	-25	0	K.FDLTMSLDDAVNEGVR.Q
85 - 100	1797.7908	1796.7835	1796.8251	-23	0	K.FDLTMSLDDAVNEGVR.Q Oxidation (M)
101 - 111	1292.6553	1291.6480	1291.6520	-3	0	R.QGYLENSNVLR.A
112 - 122	1103.5994	1102.5921	1102.5771	14	0	R.ASVLADPAFGR.K
112 - 123	1231.6764	1230.6691	1230.6721	-2	1	R.ASVLADPAFGRK.N
127 - 136	1157.6159	1156.6086	1156.5877	18	0	K.DNTPAVIHYK.L
207 - 220	1721.7759	1720.7686	1720.8011	-19	0	K.EALMEEINMDELLR.R Oxidation (M)
207 - 220	1737.7628	1736.7555	1736.7960	-23	0	K.EALMEEINMDELLR.R 2 Oxidation (M)
238 - 258	2010.1337	2009.1264	2009.1521	-13	0	K.VNALGIGAQGLGGLTTVLDIK.I
282 - 299	1912.9547	1911.9474	1911.9704	-12	0	R.HAHFQLDGSGVAHIQAPK.L
352 - 367	1877.8616	1876.8543	1876.8798	-14	0	R.MVEMIDNGEELPIDLK.G 2 Oxidation (M)
352 - 369	2031.0572	2030.0499	2030.0064	21	1	R.MVEMIDNGEELPIDLKGK.F
370 - 381	1424.7497	1423.7424	1423.7500	-5	0	K.FIYYVGPVDPVR.D
370 - 395	2776.5741	2775.5668	2775.4232	52	1	K.FIYYVGPVDPVRDEVVGPAGPTTATR.M
402 - 415	1529.8080	1528.8007	1528.8436	-28	1	R.KVLEHTGLFGMIGK.A
402 - 415	1545.8237	1544.8164	1544.8385	-14	1	R.KVLEHTGLFGMIGK.A Oxidation (M)
403 - 415	1417.7129	1416.7056	1416.7435	-27	0	K.VLEHTGLFGMIGK.A Oxidation (M)
416 - 427	1241.6685	1240.6612	1240.6775	-13	1	K.ADRGPTAIEAIK.D
431 - 446	1630.7992	1629.7919	1629.8436	-32	0	K.ATYLMAVGGAAYLVSK.A Oxidation (M)
450 - 466	1871.8725	1870.8652	1870.9022	-20	0	R.EAEVVAFADLGMEAIYK.F Oxidation (M)
467 - 491	2597.3904	2596.3831	2596.3207	24	0	K.FVVEDMPVSVAVDVNGTSIHAVAPK.I Oxidation (M)

No match to: 1275.6324, 1598.8233, 1853.9570

44.

Match to: **gi|126641383** Score: **200** Expect: **6.8e-14 Decoy: 63**
phenylacetate-CoA oxygenase subunit PaaB [Acinetobacter baumannii ATCC 17978]

Nominal mass (M_r): **11317**; Calculated pI value: **6.04**
NCBI BLAST search of gi|126641383 against nr
Unformatted sequence string for pasting into other applications

Taxonomy: Acinetobacter baumannii ATCC 17978
Links to retrieve other entries containing this sequence from NCBI Entrez:
gi|126387267 from Acinetobacter baumannii ATCC 17978
gi|183209251 from Acinetobacter baumannii ACICU

Fixed modifications: Carbamidomethyl (C)
Variable modifications: Oxidation (M)
Cleavage by Trypsin: cuts C-term side of KR unless next residue is P
Number of mass values searched: **11**
Number of mass values matched: **10**
Sequence Coverage: **82%**

Matched peptides shown in **Bold Red**

1 **MEDKNNWSLY EVFVR**SK**QGL SHRHVGSLRA PDDEIALQHA RDVYTRRNEG**
51 **ISIWVVR**SEL IKSSQPDEK**A EFFDPSLDK**V YR**HPTFYHIP DGIEHM**

Start - End	Observed	Mr(expt)	Mr(calc)	ppm	Miss	Sequence	
1 - 15	1945.8550	1944.8477	1944.9040	-29	1	-.MEDKNNWSLYEVFVR.S	Oxidation (M)
5 - 15	1426.6800	1425.6727	1425.7041	-22	0	K.NNWSLYEVFVR.S	
18 - 23	697.4300	696.4227	696.3667	80	0	K.QGLSHR.H	
24 - 29	668.4355	667.4282	667.3766	77	0	R.HVGSLR.A	
30 - 41	1335.6471	1334.6398	1334.6579	-14	0	R.APDDEIALQHAR.D	
42 - 46	653.3802	652.3729	652.3180	84	0	R.DVYTR.R	
47 - 57	1328.7231	1327.7158	1327.7361	-15	1	R.RNEGISIWVVR.S	
48 - 57	1172.6460	1171.6387	1171.6349	3	0	R.NEGISIWVVR.S	
70 - 79	1168.5601	1167.5528	1167.5448	7	0	K.AEFFDPSLDK.V	
83 - 96	1709.7201	1708.7128	1708.7668	-32	0	R.HPTFYHIPDGIEHM.-	Oxidation (M)

No match to: 1268.6160

46.

Match to: **gi|126642815** Score: **189** Expect: **8.5e-13** **Decoy: 82**
putative protease [Acinetobacter baumannii ATCC 17978]

Nominal mass (M_r): **19151**; Calculated pI value: **5.95**
NCBI BLAST search of gi|126642815 against nr
Unformatted sequence string for pasting into other applications

Taxonomy: Acinetobacter baumannii ATCC 17978

Fixed modifications: Carbamidomethyl (C)
Variable modifications: Oxidation (M)
Cleavage by Trypsin: cuts C-term side of KR unless next residue is P
Number of mass values searched: **12**
Number of mass values matched: **10**
Sequence Coverage: **56%**

Matched peptides shown in **Bold Red**

```
  1 MVPFQFLTGL GYTVHAVCPN KKNGEHIATA IHDFEGEQTY SEKRGHNFAI
 51 NYDFDAINTE DYVGLVIPGG RAPEYLRMNE RVVEIVREFD RVKKPIAAVC
101 HGAQLLAAAD VLKDRLCSAY PACAAEVKLA GGQYADIAVT EAVTDGHLVT
151 APAWPAHPAW LAQFVKVLGA KISI
```

Start - End	Observed	Mr(expt)	Mr(calc)	ppm	Miss	Sequence
23 - 43	2376.0523	2375.0450	2375.0666	-9	0	K.NGEHIATAIHDFEGEQTYSEK.R
23 - 44	2532.1899	2531.1826	2531.1677	6	1	K.NGEHIATAIHDFEGEQTYSEKR.G
45 - 71	2967.5541	2966.5468	2966.4199	43	0	R.GHNFAINYDFDAINTEDYVGLVIPGGR.A
72 - 77	748.4317	747.4244	747.3915	44	0	R.APEYLR.M
82 - 87	714.4836	713.4763	713.4436	46	0	R.VVEIVR.E
92 - 113	2273.2777	2272.2704	2272.3089	-17	1	R.VKKPIAAVCHGAQLLAAADVLK.D
94 - 113	2046.0940	2045.0867	2045.1455	-29	0	K.KPIAAVCHGAQLLAAADVLK.D
94 - 115	2317.2515	2316.2442	2316.2736	-13	1	K.KPIAAVCHGAQLLAAADVLKDR.L
114 - 128	1710.7356	1709.7283	1709.7865	-34	1	K.DRLCSAYPACAAEVK.L
116 - 128	1439.6206	1438.6133	1438.6584	-31	0	R.LCSAYPACAAEVK.L

No match to: 1436.7443, 1440.7824

47.

Match to: **gi|126640605** Score: **226** Expect: **1.7e-16** **Decoy: 69**
NADH-dependent enoyl-ACP reductase [Acinetobacter baumannii ATCC 17978]

Nominal mass (M_r): **28797**; Calculated pI value: **5.61**
NCBI BLAST search of gi|126640605 against nr
Unformatted sequence string for pasting into other applications

Taxonomy: Acinetobacter baumannii ATCC 17978

Fixed modifications: Carbamidomethyl (C)
Variable modifications: Oxidation (M)
Cleavage by Trypsin: cuts C-term side of KR unless next residue is P
Number of mass values searched: **19**
Number of mass values matched: **16**
Sequence Coverage: **47%**

Matched peptides shown in **Bold Red**

1 **MTQGLLAGKR FLIAGVASK**L SIAYGIAQAL HR**EGAELAFT YPNEK**LKK**RV**
51 **DEFAEQFGSK** LVFPCDVAVD AEIDNAFAEL AKHWDGVDGV VHSIGFAPAH
101 TLDGDFTEVT DRDGFK**IAHD ISAYSFVAMA RAAKPLLQAR QGCLLTLTYQ**
151 **GSER**VMPNYN VMGMAK**ASLE AGVRYLASSL GVDGIRVNAI SAGPIR**TLAA
201 SGIKSFR**KML DANEKVAPLK R**NVTIEEVGN AALFLCSPWA SGITGEILYV
251 DAGFNTVGMS QSMMDDE

Start - End	Observed	Mr(expt)	Mr(calc)	ppm	Miss	Sequence	
1 - 10	1074.5311	1073.5238	1073.6015	-72	1	-.MTQGLLAGKR.F	
1 - 10	1090.5321	1089.5248	1089.5965	-66	1	-.MTQGLLAGKR.F	Oxidation (M)
10 - 19	1061.6487	1060.6414	1060.6393	2	1	K.RFLIAGVASK.L	
11 - 19	905.5536	904.5463	904.5382	9	0	R.FLIAGVASK.L	
33 - 45	1468.6550	1467.6477	1467.6881	-28	0	R.EGAELAFTYPNEK.L	
49 - 60	1412.7345	1411.7272	1411.6732	38	1	K.RVDEFAEQFGSK.L	
50 - 60	1256.5713	1255.5640	1255.5721	-6	0	R.VDEFAEQFGSK.L	
117 - 131	1651.7726	1650.7653	1650.8188	-32	0	K.IAHDISAYSFVAMAR.A	
117 - 131	1667.7662	1666.7589	1666.8137	-33	0	K.IAHDISAYSFVAMAR.A	Oxidation (M)
132 - 140	967.6241	966.6168	966.5974	20	0	R.AAKPLLQAR.Q	
141 - 154	1625.7461	1624.7388	1624.7879	-30	0	R.QGCLLTLTYQGSER.V	
167 - 174	802.4673	801.4600	801.4344	32	0	K.ASLEAGVR.Y	
175 - 186	1250.6623	1249.6550	1249.6666	-9	0	R.YLASSLGVDGIR.V	
187 - 196	997.5942	996.5869	996.5716	15	0	R.VNAISAGPIR.T	
208 - 215	948.4488	947.4415	947.4746	-35	1	R.KMLDANEK.V	
216 - 221	683.4954	682.4881	682.4490	57	1	K.VAPLKR.N	

No match to: 1113.6275, 1295.5848, 1352.6844

48.

Match to: **gi|193077017** Score: **284** Expect: **2.7e-22** **Decoy: 68**
putative enoyl-CoA hydratase II [Acinetobacter baumannii ATCC 17978]

Nominal mass (M_r): **29163**; Calculated pI value: **5.76**
NCBI BLAST search of gi|193077017 against nr
Unformatted sequence string for pasting into other applications

Taxonomy: Acinetobacter baumannii ATCC 17978

Fixed modifications: Carbamidomethyl (C)
Variable modifications: Oxidation (M)
Cleavage by Trypsin: cuts C-term side of KR unless next residue is P
Number of mass values searched: **18**
Number of mass values matched: **17**
Sequence Coverage: **68%**

Matched peptides shown in **Bold Red**

```
  1 MDYQNIIAEE KNGVGYLTFN RPKALNSFNV DMHREVAEVL NQWTKNPEVR
 51 CVVISGEGRG FCAGQDLGDR VVDPNAEAPD LGYSIETYYN PLIKTIVNMP
101 KPVICAVNGV AAGAGANIAL ACDLVIAAKS ANFVQAFCRL GLVPDSAGTW
151 FLPRAVGHAR AMGLALLGDK LPAETAKEWG MIWDVVEDAE LKTKVTELAE
201 RLAKQPTFGL SLIKKAIHQS SNNTFDEQVL LERDLQRIAG RSEDYREGVQ
251 AFMNKREPNF KGR
```

Start - End	Observed	Mr(expt)	Mr(calc)	ppm	Miss	Sequence
12 - 23	1365.7034	1364.6961	1364.7201	-18	0	K.NGVGYLTFNRPK.A
24 - 34	1303.6023	1302.5950	1302.6139	-14	0	K.ALNSFNVDMHR.E
24 - 34	1319.5971	1318.5898	1318.6088	-14	0	K.ALNSFNVDMHR.E Oxidation (M)
46 - 50	614.3520	613.3447	613.3184	43	0	K.NPEVR.C
51 - 59	976.5037	975.4964	975.4808	16	0	R.CVVISGEGR.G
60 - 70	1195.5138	1194.5065	1194.5088	-2	0	R.GFCAGQDLGDR.V
71 - 94	2681.4129	2680.4056	2680.3272	29	0	R.VVDPNAEAPDLGYSIETYYNPLIK.T
130 - 139	1199.5532	1198.5459	1198.5553	-8	0	K.SANFVQAFCR.L
140 - 154	1628.8351	1627.8278	1627.8722	-27	0	R.LGLVPDSAGTWFLPR.A
161 - 177	1714.8935	1713.8862	1713.9335	-28	1	R.AMGLALLGDKLPAETAK.E Oxidation (M)
171 - 192	2546.3983	2545.3910	2545.2410	59	1	K.LPAETAKEWGMIWDVVEDAELK.T Oxidation (M)
178 - 192	1835.8084	1834.8011	1834.8447	-24	0	K.EWGMIWDVVEDAELK.T Oxidation (M)
195 - 201	817.4560	816.4487	816.4341	18	0	K.VTELAER.L
205 - 214	1103.6443	1102.6370	1102.6386	-1	0	K.QPTFGLSLIK.K
215 - 233	2229.1163	2228.1090	2228.1185	-4	1	K.KAIHQSSNNTFDEQVLLER.D
216 - 233	2101.0036	2099.9963	2100.0236	-13	0	K.AIHQSSNNTFDEQVLLER.D
242 - 255	1689.7171	1688.7098	1688.7464	-22	1	R.SEDYREGVQAFMNK.R Oxidation (M)

No match to: 1412.7697

49.

Match to: **gi|169632628** Score: **211** Expect: **9.5e-15 Decoy: 78**
succinyl-CoA synthetase subunit alpha [Acinetobacter baumannii SDF]

Nominal mass (M_r): **30845**; Calculated pI value: **5.60**
NCBI BLAST search of gi|169632628 against nr
Unformatted sequence string for pasting into other applications

Taxonomy: Acinetobacter baumannii SDF
Links to retrieve other entries containing this sequence from NCBI Entrez:
gi|169794950 from Acinetobacter baumannii AYE
gi|193078185 from Acinetobacter baumannii ATCC 17978
gi|260409423 from Acinetobacter baumannii ATCC 19606

Fixed modifications: Carbamidomethyl (C)
Variable modifications: Oxidation (M)
Cleavage by Trypsin: cuts C-term side of KR unless next residue is P
Number of mass values searched: **23**
Number of mass values matched: **18**
Sequence Coverage: **53%**

Matched peptides shown in **Bold Red**

```
  1 MSVLINKDTK VLVQGFTGKN GTFHSAQALD YGTKVVGGVT PGKGGTTHLD
 51 LPVFNTMKEA VRETNADASV IYVPAPFVLD SIVEAVDSGV GLIVVITEGV
101 PTIDMLKAKR YLETNGNGTR LVGPNCPGVI TPGECKIGIM PGHIHQPGRI
151 GIISRSGTLT YEAVAQTTKL GLGQSTCIGI GGDPIPGMNQ IEALQLFQDD
201 PDTDAIIMIG EIGGTAEEEA AEFIKSNVTK PVVGYIAGVT APKGKRMGHA
251 GAIISGGQGT AEEKFAAFEK AGMAYTRSPA ELGSTMLQVL KEKGLA
```

Start - End	Observed	Mr(expt)	Mr(calc)	ppm	Miss	Sequence	
11 - 19	948.6029	947.5956	947.5440	54	0	K.VLVQGFTGK.N	
20 - 34	1609.7204	1608.7131	1608.7532	-25	0	K.NGTFHSAQALDYGTK.V	
44 - 58	1630.7581	1629.7508	1629.8185	-42	0	K.GGTTHLDLPVFNTMK.E	
44 - 58	1646.7827	1645.7754	1645.8134	-23	0	K.GGTTHLDLPVFNTMK.E	Oxidation (M)
110 - 120	1280.6144	1279.6071	1279.6269	-15	1	K.RYLETNGNGTR.L	
111 - 120	1124.5296	1123.5223	1123.5258	-3	0	R.YLETNGNGTR.L	
121 - 136	1697.7950	1696.7877	1696.8277	-24	0	R.LVGPNCPGVITPGECK.I	
137 - 149	1412.7279	1411.7206	1411.7507	-21	0	K.IGIMPGHIHQPGR.I	
137 - 149	1428.7332	1427.7259	1427.7456	-14	0	K.IGIMPGHIHQPGR.I	Oxidation (M)
150 - 155	658.4582	657.4509	657.4173	51	0	R.IGIISR.S	
156 - 169	1469.7258	1468.7185	1468.7409	-15	0	R.SGTLTYEAVAQTTK.L	
226 - 243	1800.9803	1799.9730	1800.0145	-23	0	K.SNVTKPVVGYIAGVTAPK.G	
246 - 264	1885.8716	1884.8643	1884.9112	-25	1	K.RMGHAGAIISGGQGTAEEK.F	Oxidation (M)
247 - 264	1713.7849	1712.7776	1712.8152	-22	0	R.MGHAGAIISGGQGTAEEK.F	

247 - 264	1729.7762	1728.7689	1728.8101	-24	0	R.MGHAGAIISGGQGTAEEK.F Oxidation (M)
271 - 277	769.3961	768.3888	768.3588	39	0	K.AGMAYTR.S
271 - 277	785.3920	784.3847	784.3538	39	0	K.AGMAYTR.S Oxidation (M)
278 - 291	1489.7629	1488.7556	1488.7858	-20	0	R.SPAELGSTMLQVLK.E Oxidation (M)

No match to: 1364.7318, 1372.1976, 1444.7207, 1490.6559, 1822.9546

50.

Match to: **gi|169794791** Score: **174** Expect: **4.8e-11** **Decoy: 83**
acetylCoA carboxylase, beta subunit [Acinetobacter baumannii AYE]

Nominal mass (M_r): **33293**; Calculated pI value: **5.85**
NCBI BLAST search of gi|169794791 against nr
Unformatted sequence string for pasting into other applications

Taxonomy: Acinetobacter baumannii AYE
Links to retrieve other entries containing this sequence from NCBI Entrez:
gi|184159436 from Acinetobacter baumannii ACICU
gi|281312110 from Acinetobacter baumannii ATCC 17978
gi|260409262 from Acinetobacter baumannii ATCC 19606

Fixed modifications: Carbamidomethyl (C)
Variable modifications: Oxidation (M)
Cleavage by Trypsin: cuts C-term side of KR unless next residue is P
Number of mass values searched: **16**
Number of mass values matched: **14**
Sequence Coverage: **44%**

Matched peptides shown in **Bold Red**

```
  1 MNQEVKSGKV LSPSTPWTQR PVPGIEVADE QQTLKATFTE PTIECPECHA
 51 LVTRTAISFN AYVCPQCDEH LRMKARDRLN WFFDNVVAEL GQEFSAKDPL
101 KFVDSKPYPD RMREAQTKTG ETEALIAMQG NLNGVDMIAC AFEFDFMGGS
151 MGTVVGDRFV KAAELAIEKR QPLICFAASG GARMQEGMLS LMQMARTSAA
201 IQKLKDAGLP YIVVLTHPVY GGVTASLAML GDIHIAEPKA MIGFAGKRVI
251 EQTVRETLEE PFQRAEYLLD HGVVDQIVHR HALRDTVSRL VSKLMNLP
```

Start - End	Observed	Mr(expt)	Mr(calc)	ppm	Miss	Sequence
10 - 35	2876.6555	2875.6482	2875.5080	49	0	K.VLSPSTPWTQRPVPGIEVADEQQTLK.A
36 - 54	2232.0481	2231.0408	2231.0351	3	0	K.ATFTEPTIECPECHALVTR.T
55 - 72	2180.9773	2179.9700	2179.9779	-4	0	R.TAISFNAYVCPQCDEHLR.M
102 - 111	1223.6271	1222.6198	1222.5982	18	0	K.FVDSKPYPDR.M
170 - 183	1503.7784	1502.7711	1502.7776	-4	1	K.RQPLICFAASGGAR.M
171 - 183	1347.6878	1346.6805	1346.6765	3	0	R.QPLICFAASGGAR.M

**184 - 196**	**1525.7525**	**1524.7452**	**1524.6921**	**35**	**0**	**R.MQEGMLSLMQMAR.T**	
**184 - 196**	**1541.7303**	**1540.7230**	**1540.6870**	**23**	**0**	**R.MQEGMLSLMQMAR.T**	Oxidation (M)
**184 - 196**	**1573.6820**	**1572.6747**	**1572.6768**	**-1**	**0**	**R.MQEGMLSLMQMAR.T**	3 Oxidation (M)
**184 - 196**	**1589.6716**	**1588.6643**	**1588.6717**	**-5**	**0**	**R.MQEGMLSLMQMAR.T**	4 Oxidation (M)
**248 - 255**	**1000.6357**	**999.6284**	**999.5825**	**46**	**1**	**K.RVIEQTVR.E**	
**249 - 255**	**844.5359**	**843.5286**	**843.4814**	**56**	**0**	**R.VIEQTVR.E**	
**256 - 264**	**1148.5822**	**1147.5749**	**1147.5509**	**21**	**0**	**R.ETLEEPFQR.A**	
**265 - 280**	**1863.9534**	**1862.9461**	**1862.9639**	**-10**	**0**	**R.AEYLLDHGVVDQIVHR.H**	

No match to: 1256.6973, 1412.7700

51.

Match to: **gi|169796334** Score: **254** Expect: **4.8e-19 Decoy: 70**
putative Acyl-CoA dehydrogenase [Acinetobacter baumannii AYE]

Nominal mass (M_r): **42531**; Calculated pI value: **5.81**
NCBI BLAST search of gi|169796334 against nr
Unformatted sequence string for pasting into other applications

Taxonomy: Acinetobacter baumannii AYE
Links to retrieve other entries containing this sequence from NCBI Entrez:
gi|183209325 from Acinetobacter baumannii ACICU
gi|193077045 from Acinetobacter baumannii ATCC 17978

Fixed modifications: Carbamidomethyl (C)
Variable modifications: Oxidation (M)
Cleavage by Trypsin: cuts C-term side of KR unless next residue is P
Number of mass values searched: **25**
Number of mass values matched: **20**
Sequence Coverage: **61%**

Matched peptides shown in **Bold Red**

1 MNLRSLNFGL DETLIALQDS VAAFCAK**EIA** **PIAQQVDQDN** **KFPAHLWK**KF
51 GDMGLLGMTV SEEYGGANMG YLAHIIAMQE ISR**ASAAIGL** **SYGAHSNLCV**
101 **NQINR**NGNEQ QKQKYLPK**LI** **SGEYVGALAM** **SEPNAGSDVV** **SMK**LR**AEQKG**
151 **DHFVLNGSKM** **WITNGGDADV** **LVVYAK**TDPQ AGPKGMTAFL IEKGMK**GFSH**
201 **GNHLDKLGMR** **GSNTYPLFFD** **NVEVPAENVL** **GGVGNGVKVL** **MSGLDYER**AV
251 LSAGPLGIMD ACLDVVIPYL HQR**EQFGQAL** **GEFQLMQGKL** **ADMYSTWLAC**
301 **KALVYAVGAA** **CDK**ADHDRSL RK**DAASAILY** **AAEKATWMAG** **EAIQTLGGNG**
351 **YINEFPAGR**L WRDAK**LYEIG** **AGTSEIRR**ML IGRELFNETK

Start - End	Observed	Mr(expt)	Mr(calc)	ppm	Miss	Sequence
**28 - 48**	**2448.3142**	**2447.3069**	**2447.2597**	**19**	**1**	**K.EIAPIAQQVDQDNKFPAHLWK.K**
**42 - 48**	**898.5430**	**897.5357**	**897.4861**	**55**	**0**	**K.FPAHLWK.K**

84 - 105	2316.1771	2315.1698	2315.1440	11	0	R.ASAAIGLSYGAHSNLCVNQINR.N	
119 - 143	2557.2756	2556.2683	2556.2087	23	0	K.LISGEYVGALAMSEPNAGSDVVSMK.L	2 Oxidation (M)
146 - 159	1529.7420	1528.7347	1528.7634	-19	1	R.AEQKGDHFVLNGSK.M	
150 - 159	1073.6121	1072.6048	1072.5302	70	0	K.GDHFVLNGSK.M	
160 - 176	1867.9127	1866.9054	1866.9186	-7	0	K.MWITNGGDADVLVVYAK.T	Oxidation (M)
197 - 206	1111.5559	1110.5486	1110.5207	25	0	K.GFSHGNHLDK.L	
197 - 210	1568.7717	1567.7644	1567.7678	-2	1	K.GFSHGNHLDKLGMR.G	
211 - 238	2893.5803	2892.5730	2892.4294	50	0	R.GSNTYPLFFDNVEVPAENVLGGVGNGVK.V	
239 - 248	1182.6034	1181.5961	1181.5750	18	0	K.VLMSGLDYER.A	
239 - 248	1198.5987	1197.5914	1197.5700	18	0	K.VLMSGLDYER.A	Oxidation (M)
274 - 289	1826.8604	1825.8531	1825.8669	-8	0	R.EQFGQALGEFQLMQGK.L	Oxidation (M)
290 - 301	1474.6693	1473.6620	1473.6632	-1	0	K.LADMYSTWLACK.A	Oxidation (M)
302 - 313	1237.6283	1236.6210	1236.6172	3	0	K.ALVYAVGAACDK.A	
323 - 334	1222.6404	1221.6331	1221.6241	7	0	K.DAASAILYAAEK.A	
335 - 359	2624.2957	2623.2884	2623.2489	15	0	K.ATWMAGEAIQTLGGNGYINEFPAGR.L	
335 - 359	2640.3355	2639.3282	2639.2438	32	0	K.ATWMAGEAIQTLGGNGYINEFPAGR.L	Oxidation (M)
366 - 377	1308.6934	1307.6861	1307.6721	11	0	K.LYEIGAGTSEIR.R	
366 - 378	1464.7788	1463.7715	1463.7732	-1	1	K.LYEIGAGTSEIRR.M	

No match to: 1191.6574, 1243.5842, 1271.7274, 1606.7676, 2127.1335

52.
Match to: **gi|294859738** Score: **326** Expect: **1.7e-26**
urocanate hydratase [Acinetobacter sp. 6013150]

Nominal mass (M_r): **61260**; Calculated pI value: **5.64**
NCBI BLAST search of gi|294859738 against nr
Unformatted sequence string for pasting into other applications

Taxonomy: Acinetobacter baumannii 6013150
Links to retrieve other entries containing this sequence from NCBI Entrez:
gi|301346124 from Acinetobacter baumannii AB056
gi|301513253 from Acinetobacter baumannii AB058
gi|301598029 from Acinetobacter baumannii AB059

Fixed modifications: Carbamidomethyl (C)
Variable modifications: Oxidation (M)
Cleavage by Trypsin: cuts C-term side of KR unless next residue is P
Number of mass values searched: **28**
Number of mass values matched: **26**
Sequence Coverage: **46%**

Matched peptides shown in **Bold Red**

1 MTTK**FRDVEI R**APRGTELTA K**SWLTEAPLR MLMNNLDPDV AENPKELVVY**

```
 51 GGIGRAARNW ECFDKIVDTL KNLETDETLL VQSGKPVGVF KTHKDAPRVL
101 IANSNLVPHW ANWEHFNELD AKALAMYGQM TAGSWIYIGS QGIVQGTYET
151 FVEAGRQHYN GDLKGRWVLT AGLGGMGGAQ PLAATLAGAC SLNIECQQAS
201 IDFRLRTRYV DEQATDLDDA LARIDRYTKE GKAISIALHG NAAEILPELV
251 RRGVRPDMVT DQTSAHDPLN GYLPVGWTWD EYRERAKKEP EAVVKAAKQS
301 MAKHVQAMLD FQKMGVPTFD YGNNIRQMAK EEGVANAFDF PGFVPAYIRP
351 LFCRGIGPFR WAALSGDPED IYKTDAKVKE LIPDDEHLHH WLDMARERIS
401 FQGLPARICW VGLGLRAKLG LAFNEMVRSG ELSAPIVIGR DHLDSGSVAS
451 PNRETEAMQD GSDAVSDWPL LNALLNTAGG ATWVSLHHGG GVGMGFSQHS
501 GVVIVCDGTD EAAARIARVL TNDPATGVMR HADAGYEIAI NCAKEQGLHL
551 PMITQ
```

Start - End	Observed	Mr(expt)	Mr(calc)	ppm	Miss	Sequence
5 - 11	934.5545	933.5472	933.5032	47	1	K.FRDVEIR.A
22 - 30	1072.6105	1071.6032	1071.5713	30	0	K.SWLTEAPLR.M
31 - 45	1716.8202	1715.8129	1715.7858	16	0	R.MLMNNLDPDVAENPK.E Oxidation (M)
46 - 55	1062.6224	1061.6151	1061.5869	27	0	K.ELVVYGGIGR.A
59 - 65	998.4576	997.4503	997.3964	54	0	R.NWECFDK.I
72 - 91	2174.1477	2173.1404	2173.1631	-10	0	K.NLETDETLLVQSGKPVGVFK.T
92 - 98	824.4989	823.4916	823.4300	75	1	K.THKDAPR.V
207 - 223	1951.8994	1950.8921	1950.9283	-19	1	R.TRYVDEQATDLDDALAR.I
209 - 223	1694.7607	1693.7534	1693.7795	-15	0	R.YVDEQATDLDDALAR.I
233 - 251	1987.1021	1986.0948	1986.1262	-16	0	K.AISIALHGNAAEILPELVR.R
304 - 313	1232.6174	1231.6101	1231.6019	7	0	K.HVQAMLDFQK.M Oxidation (M)
314 - 326	1483.6801	1482.6728	1482.6926	-13	0	K.MGVPTFDYGNNIR.Q
314 - 326	1499.6832	1498.6759	1498.6875	-8	0	K.MGVPTFDYGNNIR.Q Oxidation (M)
355 - 360	646.4256	645.4183	645.3598	91	0	R.GIGPFR.W
361 - 373	1464.7164	1463.7091	1463.6932	11	0	R.WAALSGDPEDIYK.T
380 - 396	2142.9559	2141.9486	2141.9953	-22	0	K.ELIPDDEHLHHWLDMAR.E Oxidation (M)
399 - 407	988.5953	987.5880	987.5502	38	0	R.ISFQGLPAR.I
408 - 416	1073.6233	1072.6160	1072.5852	29	0	R.ICWVGLGLR.A
419 - 428	1149.6272	1148.6199	1148.6012	16	0	K.LGLAFNEMVR.S
419 - 428	1165.6225	1164.6152	1164.5961	16	0	K.LGLAFNEMVR.S Oxidation (M)
429 - 440	1198.6936	1197.6863	1197.6717	12	0	R.SGELSAPIVIGR.D
441 - 453	1354.6334	1353.6261	1353.6273	-1	0	R.DHLDSGSVASPNR.E
519 - 530	1273.6592	1272.6519	1272.6496	2	0	R.VLTNDPATGVMR.H
519 - 530	1289.6576	1288.6503	1288.6445	4	0	R.VLTNDPATGVMR.H Oxidation (M)
531 - 544	1532.7025	1531.6952	1531.7089	-9	0	R.HADAGYEIAINCAK.E
545 - 555	1282.6492	1281.6419	1281.6387	3	0	K.EQGLHLPMITQ.- Oxidation (M)

53.
Match to: **gi|126642744** Score: **401** Expect: **5.4e-34** **Decoy: 68**
succinate dehydrogenase flavoprotein subunit [Acinetobacter baumannii ATCC 17978]

Nominal mass (M_r): **64505**; Calculated pI value: **6.07**
NCBI BLAST search of gi|126642744 against nr
Unformatted sequence string for pasting into other applications

Taxonomy: Acinetobacter baumannii ATCC 17978

Fixed modifications: Carbamidomethyl (C)
Variable modifications: Oxidation (M)
Cleavage by Trypsin: cuts C-term side of KR unless next residue is P
Number of mass values searched: **39**
Number of mass values matched: **34**
Sequence Coverage: **62%**

Matched peptides shown in **Bold Red**

```
  1 MRASYQLAQA GLKVAVLTKV FPTRSHTVAA QGGIGASLGN MQEDNWHFHF
 51 YDTVKGSDWL GDQDAIEFMC REAPKVVYEL EHLGMPFDRN ADGTIYQRPF
101 GGHSANYGDK PVPRACAAAD RTGHALLHTL YQSNVKMGTQ FFVEWIALDL
151 IRNEAGDVLG VTAIDQETGN IAVFQAKATL FATGGAGRVY RASTNAYINT
201 GDGLGMAARA GIPLQDMEFW QFHPTGVAGA GVLLTEGCRG EGAILRNKDG
251 EPFMERYAPT LKDLAPRDFV SRSMDQEIKE GRGCGPKGDY ILLDMTHLGA
301 DTIMKRLPSV FEIGKKFANV DITKEPIPVV PTIHYQMGGI PTNMHGQVCL
351 PEPGTDNYTK PVKGFYAIGE CSCVSVHGAN RLGTNSLLDL VVFGKAAGEH
401 IIDYVTKHHG DEYAPLPTNV LEQTLARVRK LDESTSGENA QEVADAIRDI
451 VQDHAGVFRT QALLDKGVKE ILALEPRVRN IHLKDKSKVF NTARVEALEV
501 ENLYEVAKAT LISAAARKEC RGAHTVVDYE LPADHPTYSY GRRDDEWMKH
551 TLWYSSDNRL EYKPVRFKPL TVDPIPPAPR TF
```

Start - End	Observed	Mr(expt)	Mr(calc)	ppm	Miss	Sequence
3 - 13	1149.6295	1148.6222	1148.6189	3	0	R.ASYQLAQAGLK.V
20 - 24	619.4053	618.3980	618.3490	79	0	K.VFPTR.S
56 - 71	1899.7515	1898.7442	1898.7927	-26	0	K.GSDWLGDQDAIEFMCR.E
56 - 71	1915.7494	1914.7421	1914.7877	-24	0	K.GSDWLGDQDAIEFMCR.E Oxidation (M)
76 - 89	1704.8084	1703.8011	1703.8341	-19	0	K.VVYELEHLGMPFDR.N
76 - 89	1720.7939	1719.7866	1719.8290	-25	0	K.VVYELEHLGMPFDR.N Oxidation (M)
90 - 114	2717.3769	2716.3696	2716.3106	22	0	R.NADGTIYQRPFGGHSANYGDKPVPR.A
122 - 136	1681.8607	1680.8534	1680.8947	-25	0	R.TGHALLHTLYQSNVK.M
153 - 177	2560.3031	2559.2958	2559.2817	6	0	R.NEAGDVLGVTAIDQETGNIAVFQAK.A
178 - 188	1021.5629	1020.5556	1020.5352	20	0	K.ATLFATGGAGR.V
192 - 209	1782.7956	1781.7883	1781.8366	-27	0	R.ASTNAYINTGDGLGMAAR.A
192 - 209	1798.7955	1797.7882	1797.8315	-24	0	R.ASTNAYINTGDGLGMAAR.A Oxidation (M)
247 - 256	1222.5512	1221.5439	1221.5448	-1	1	R.NKDGEPFMER.Y
247 - 256	1238.5446	1237.5373	1237.5397	-2	1	R.NKDGEPFMER.Y Oxidation (M)

268 - 279	1454.6847	1453.6774	1453.6871	-7	1	R.DFVSRSMDQEIK.E
306 - 315	1145.6747	1144.6674	1144.6604	6	1	K.RLPSVFEIGK.K
307 - 316	1117.6729	1116.6656	1116.6543	10	1	R.LPSVFEIGKK.F
364 - 381	1983.8376	1982.8303	1982.8727	-21	0	K.GFYAIGECSCVSVHGANR.L
382 - 395	1475.7634	1474.7561	1474.8395	-57	0	R.LGTNSLLDLVVFGK.A
396 - 407	1316.6759	1315.6686	1315.6772	-7	0	K.AAGEHIIDYVTK.H
408 - 427	2261.1039	2260.0966	2260.1236	-12	0	K.HHGDEYAPLPTNVLEQTLAR.V
408 - 429	2516.1824	2515.1751	2515.2932	-47	1	K.HHGDEYAPLPTNVLEQTLARVR.K
430 - 448	2032.9370	2031.9297	2031.9709	-20	1	R.KLDESTSGENAQEVADAIR.D
431 - 448	1904.8394	1903.8321	1903.8759	-23	0	K.LDESTSGENAQEVADAIR.D
449 - 459	1256.6337	1255.6264	1255.6310	-4	0	R.DIVQDHAGVFR.T
470 - 477	940.5785	939.5712	939.5389	34	0	K.EILALEPR.V
489 - 494	707.4308	706.4235	706.3762	67	0	K.VFNTAR.V
495 - 508	1605.7987	1604.7914	1604.8297	-24	0	R.VEALEVENLYEVAK.A
509 - 517	873.5467	872.5394	872.5079	36	0	K.ATLISAAAR.K
522 - 542	2348.0814	2347.0741	2347.0869	-5	0	R.GAHTVVDYELPADHPTYSYGR.R
543 - 549	995.4465	994.4392	994.4178	22	1	R.RDDEWMK.H Oxidation (M)
550 - 559	1278.5772	1277.5699	1277.5789	-7	0	K.HTLWYSSDNR.L
560 - 566	904.5584	903.5511	903.5178	37	0	R.LEYKPVR.F
567 - 580	1547.8604	1546.8531	1546.8871	-22	0	R.FKPLTVDPIPPAPR.T

No match to: 1273.7561, 1532.6875, 1664.1339, 1698.7901, 2500.1895

55.

Match to: **gi|169632218** Score: **299** Expect: **8.5e-24** **Decoy: 81**
osmolarity response regulator [Acinetobacter baumannii SDF]

Nominal mass (M_r): **28854**; Calculated pI value: **5.91**
NCBI BLAST search of gi|169632218 against nr
Unformatted sequence string for pasting into other applications

Taxonomy: Acinetobacter baumannii SDF
Links to retrieve other entries containing this sequence from NCBI Entrez:
gi|169794452 from Acinetobacter baumannii AYE
gi|193078590 from Acinetobacter baumannii ATCC 17978
gi|260409491 from Acinetobacter baumannii ATCC 19606

Fixed modifications: Carbamidomethyl (C)
Variable modifications: Oxidation (M)
Cleavage by Trypsin: cuts C-term side of KR unless next residue is P
Number of mass values searched: **26**
Number of mass values matched: **22**
Sequence Coverage: **70%**

Matched peptides shown in **Bold Red**

```
      1 MSLVVPAEHP ETVHNETDRV ERILVVDDDV RLRTLLQRFL EDKGFVVKTA
     51 HDASQMDRLL QRELFSLIVL DFMLPVEDGL SICRRLRQSN IDTPIIMLTA
    101 RGSDSDRIAG LEAGADDYLP KPFNPNELLA RIRAVLRRQV REVPGAPSQQ
    151 VEVVSFGPWS LDLSTRTLTR EGQIVTLTTG EFAVLKALVQ HPREPLTRDK
    201 LMNLARGREW GAMERSIDVQ VSRLRRLIED NPARARYIQT VWGVGYVFVP
    251 DGAE
```

Start - End	Observed	Mr(expt)	Mr(calc)	ppm	Miss	Sequence
2 - 19	2029.9564	2028.9491	2028.9865	-18	0	M.SLVVPAEHPETVHNETDR.V
2 - 22	2414.1984	2413.1911	2413.1986	-3	1	M.SLVVPAEHPETVHNETDRVER.I
23 - 31	1043.5911	1042.5838	1042.5659	17	0	R.ILVVDDDVR.L
34 - 38	630.4360	629.4287	629.3860	68	0	R.TLLQR.F
49 - 58	1131.4859	1130.4786	1130.4775	1	0	K.TAHDASQMDR.L
49 - 58	1147.4953	1146.4880	1146.4724	14	0	K.TAHDASQMDR.L Oxidation (M)
49 - 62	1657.7486	1656.7413	1656.8002	-36	1	K.TAHDASQMDRLLQR.E Oxidation (M)
88 - 101	1572.8043	1571.7970	1571.8341	-24	0	R.QSNIDTPIIMLTAR.G
88 - 101	1588.8077	1587.8004	1587.8290	-18	0	R.QSNIDTPIIMLTAR.G Oxidation (M)
108 - 131	2584.3638	2583.3565	2583.3333	9	0	R.IAGLEAGADDYLPKPFNPNELLAR.I
134 - 138	614.4455	613.4382	613.4024	58	1	R.AVLRR.Q
142 - 166	2685.3923	2684.3850	2684.3446	15	0	R.EVPGAPSQQVEVVSFGPWSLDLSTR.T
171 - 186	1705.8982	1704.8909	1704.9298	-23	0	R.EGQIVTLTTGEFAVLK.A
187 - 193	820.5195	819.5122	819.4715	50	0	K.ALVQHPR.E
194 - 198	615.3877	614.3804	614.3388	68	0	R.EPLTR.D
199 - 206	976.5532	975.5459	975.5171	30	1	R.DKLMNLAR.G Oxidation (M)
207 - 215	1091.5216	1090.5143	1090.4978	15	1	R.GREWGAMER.S
207 - 215	1107.5182	1106.5109	1106.4927	16	1	R.GREWGAMER.S Oxidation (M)
209 - 215	894.4201	893.4128	893.3701	48	0	R.EWGAMER.S Oxidation (M)
216 - 223	903.5196	902.5123	902.4821	33	0	R.SIDVQVSR.L
226 - 234	1083.6064	1082.5991	1082.5832	15	1	R.RLIEDNPAR.A
227 - 234	927.5228	926.5155	926.4821	36	0	R.LIEDNPAR.A

No match to: 1123.5116, 1170.6309, 1532.1928, 1727.8710

56.
Match to: **gi|193077019** Score: **336** Expect: **1.7e-27 Decoy: 69**
Thiolase [Acinetobacter baumannii ATCC 17978]

Nominal mass (M_r): **43561**; Calculated pI value: **6.08**
NCBI BLAST search of gi|193077019 against nr
Unformatted sequence string for pasting into other applications

Taxonomy: Acinetobacter baumannii ATCC 17978

Fixed modifications: Carbamidomethyl (C)
Variable modifications: Oxidation (M)
Cleavage by Trypsin: cuts C-term side of KR unless next residue is P
Number of mass values searched: **25**
Number of mass values matched: **23**
Sequence Coverage: **58%**

Matched peptides shown in **Bold Red**

1 **MEDVLICDFI RTPIGRYAGA LSAVR**ADDLA ALPIK**YLKDK** HPNLPWNTVD
51 EVFLGCANQA GEDNRNVAR**M ATLLAGLPDT VPAMTVNRLC ASGLDAVGLA**
101 **AR**SIK**AGEAQ FVLAGGVESM SRAPFVQAKP TEAFSRTPEI YDTTIGWR**FV
151 NKQLKAQYGT DSMPETAENV AEKYQISR**ED QDAFALR**SQQ K**TAAAQQNGF**
201 **FNDEILPVEI TDRK**K**NVVVV NRDEHPR**ETT LEALAK**LKAP FK**K**EGGSVTA**
251 **GNASGVNDGA ACVLITNREF ANTYGLKPLA RVIGIASVGV EPKYMGIGPV**
301 **PAVQK**ILKQT GLTLDQMDVI ELNEAFAAQS LACMR**ELGLK DDDER**VNPNG
351 GAIALGHPLG MSGTR**LVITA TR**ELKKRGGR YALCTMCVGV GQGVALILEN
401 LN

Start - End	Observed	Mr(expt)	Mr(calc)	ppm	Miss	Sequence
1 - 11	1410.6627	1409.6554	1409.6683	-9	0	-.MEDVLICDFIR.T
1 - 11	1426.6393	1425.6320	1425.6632	-22	0	-.MEDVLICDFIR.T Oxidation (M)
1 - 16	1934.9241	1933.9168	1933.9754	-30	1	-.MEDVLICDFIRTPIGR.Y
17 - 25	907.5274	906.5201	906.4923	31	0	R.YAGALSAVR.A
36 - 40	666.3970	665.3897	665.3748	22	1	K.YLKDK.H
70 - 88	2002.9666	2001.9593	2002.0227	-32	0	R.MATLLAGLPDTVPAMTVNR.L 2 Oxidation (M)
89 - 102	1373.6927	1372.6854	1372.7133	-20	0	R.LCASGLDAVGLAAR.S
106 - 122	1724.7720	1723.7647	1723.8199	-32	0	K.AGEAQFVLAGGVESMSR.A Oxidation (M)
123 - 136	1548.7733	1547.7660	1547.8096	-28	0	R.APFVQAKPTEAFSR.T
137 - 148	1451.6828	1450.6755	1450.7092	-23	0	R.TPEIYDTTIGWR.F
179 - 187	1064.5085	1063.5012	1063.4934	7	0	R.EDQDAFALR.S
192 - 213	2449.1791	2448.1718	2448.1921	-8	0	K.TAAAQQNGFFNDEILPVEITDR.K
192 - 214	2577.2917	2576.2844	2576.2871	-1	1	K.TAAAQQNGFFNDEILPVEITDRK.K
216 - 222	799.5124	798.5051	798.4712	42	0	K.NVVVVNR.D
216 - 227	1433.7307	1432.7234	1432.7535	-21	1	K.NVVVVNRDEHPR.E
223 - 227	653.3440	652.3367	652.2929	67	0	R.DEHPR.E
237 - 242	703.4113	702.4040	702.4428	-55	1	K.LKAPFK.K
244 - 268	2390.1078	2389.1005	2389.1292	-12	0	K.EGGSVTAGNASGVNDGAACVLITNR.E
269 - 281	1479.7593	1478.7520	1478.7881	-24	0	R.EFANTYGLKPLAR.V
282 - 293	1168.6891	1167.6818	1167.6863	-4	0	R.VIGIASVGVEPK.Y
294 - 305	1275.6663	1274.6590	1274.6693	-8	0	K.YMGIGPVPAVQK.I Oxidation (M)
336 - 345	1189.5594	1188.5521	1188.5622	-8	1	R.ELGLKDDDER.V
366 - 372	773.5276	772.5203	772.4807	51	0	R.LVITATR.E

No match to: 1467.6751, 1668.1084
